# Bridging the gap between research, policy, and practice: Lessons learned from academic–public partnerships in the CTSA network

**DOI:** 10.1017/cts.2020.23

**Published:** 2020-03-10

**Authors:** Amytis Towfighi, Allison Zumberge Orechwa, Tomás J. Aragón, Marc Atkins, Arleen F. Brown, Jen Brown, Olveen Carrasquillo, Savanna Carson, Paula Fleisher, Erika Gustafson, Deborah K. Herman, Moira Inkelas, Wylie Liu, Daniella Meeker, Tara Mehta, Doriane C. Miller, Rachelle Paul-Brutus, Michael B. Potter, Sarah S. Ritner, Brendaly Rodriguez, Dana Rusch, Anne Skinner, Hal F. Yee

**Affiliations:** 1 Southern California Clinical and Translational Sciences Institute, University of Southern California, Los Angeles, CA, USA; 2 Los Angeles County Department of Health Services, Los Angeles, CA, USA; 3 San Francisco Department of Public Health, San Francisco, CA, USA; 4 Center for Clinical Translational Science, University of Illinois at Chicago, Chicago, IL, USA; 5 University of California Los Angeles Clinical and Translational Science Institute, Los Angeles, CA, USA; 6 Northwestern University Clinical and Translational Sciences Institute, Chicago, IL, USA; 7 University of Miami Clinical and Translational Sciences Institute, Miami, FL, USA; 8 University of California San Francisco Clinical and Translational Science Institute, San Francisco, CA, USA; 9 Institute for Translational Medicine, University of Chicago, Chicago, IL, USA; 10 Chicago Department of Public Health, Chicago, IL, USA; 11 Alliance Chicago, Chicago, IL, USA

**Keywords:** Translational research, policy-relevant research, implementation science, community engagement, public health

## Abstract

A primary barrier to translation of clinical research discoveries into care delivery and population health is the lack of sustainable infrastructure bringing researchers, policymakers, practitioners, and communities together to reduce silos in knowledge and action. As National Institutes of Healthʼs (NIH) mechanism to advance translational research, Clinical and Translational Science Award (CTSA) awardees are uniquely positioned to bridge this gap. Delivering on this promise requires sustained collaboration and alignment between research institutions and public health and healthcare programs and services. We describe the collaboration of seven CTSA hubs with city, county, and state healthcare and public health organizations striving to realize this vision together. Partnership representatives convened monthly to identify key components, common and unique themes, and barriers in academic–public collaborations. All partnerships aligned the activities of the CTSA programs with the needs of the city/county/state partners, by sharing resources, responding to real-time policy questions and training needs, promoting best practices, and advancing community-engaged research, and dissemination and implementation science to narrow the knowledge-to-practice gap. Barriers included competing priorities, differing timelines, bureaucratic hurdles, and unstable funding. Academic–public health/health system partnerships represent a unique and underutilized model with potential to enhance community and population health.

## Introduction

The translation of research discoveries from “bench to bedside” and into improved health is slow and inefficient [1]. The attempt to bridge science, policy, and practice has been described as a “valley of death,” reflecting few successful enduring outcomes [2]. Federal investment in basic science and efficacy research dwarfs the investment in health quality, dissemination, and outcomes research [3]. Although social determinants of health account for approximately 60% of health outcomes [4], the United States spends a significantly lower percentage of its gross domestic product (GDP) on social services as compared to similar countries with better health outcomes [5], and only 5% of U.S. national health expenditures are allocated to population-wide approaches to health promotion [6]. Widespread adoption of evidence into policy and practice is hampered when academic institutions undertake science in controlled settings and conditions. Additionally, evidence-based practices resulting from academic studies often result in limited dissemination even within academic circles. Yet, public agencies, including safety-net healthcare systems and departments of public health, must respond to and implement evidence-based policies and health promotion services for populations facing higher burdens of health and healthcare disparities.

More researchers are turning to dissemination and implementation science (D&I) methods to more effectively bridge the research-to-practice gap [7]. Yet, despite a century of empirical research to advance the translation of research to practice, considerable barriers remain, especially for advancing public health policy and practice [8].

A primary challenge to addressing the research-policy-practice gap is the lack of sustainable infrastructure bringing researchers, policymakers, practitioners, and communities together to: (1) align the research enterprise with public and population health priorities; (2) bridge healthcare, public health, mental health, and related sectors; (3) engage health systems in research; and (4) develop innovative solutions for health systems. Without a formal mechanism to effectively engage the community, academicians, and public health and healthcare agencies, research fails to address the need among most public health and healthcare agencies to increase the quality of services with existing resources.

Institutions with Clinical and Translational Science Awards (CTSAs) are uniquely positioned to bridge this gap and contribute to care delivery, translation of research into interventions that improve the health of communities, and public health innovation. In 2006, National Institutes of Health (NIH) launched the CTSA Program to support a national network of medical research institutions, or “hubs,” that provide infrastructure at their local universities and other affiliated academic partners to advance clinical and translational research and population health. Hubs support research across disciplines and promote team-based science closely integrated with patients and communities. Their education and training programs aim to create the next generation of translational scientists who are “boundary crossers” and “systems thinkers” [9]. Through their collaboration with communities, hubs are uniquely situated to identify local health priorities, as well as the resources and expertise to catalyze research in those areas. The CTSA network holds great promise for bridging the research-policy-practice gap.

A number of CTSA hubs have a major emphasis on partnering with city, county, and state health organizations to drive innovations in clinical care and translate research into practical interventions that improve community and population health. We will describe examples from seven CTSA hubs in four cities – Los Angeles, Chicago, Miami, and San Francisco – that have activated their resources toward research, effective service delivery, policy development, implementation, and program evaluation.

## Synergy Paper Collaboration

With support from the CTSA Coordinating Center through a “Synergy paper” mechanism, representatives from the seven CTSA hubs and public health/health system partners participated in monthly teleconferences to collaborate on developing a manuscript on this shared topic. The earlier teleconferences included a brief overview by participants of their existing academic–public health/health system partnerships and discussions on shared experiences, lessons learned, and future directions. This led to more in-depth conversations, addressing common themes and both mutual and unique barriers to achieving goals. As the linkage to respective health systems was crucial to this evaluation, authors from each CTSA hub collaborated closely with key public health and health system representatives and received written comments and feedback to integrate into the manuscript. After the elicitation and information sharing processes, members categorized critical factors, challenges, and opportunities for improvement and strategized on recommendations. As a group, members summarized activities and assessed similarities.

## CTSA-Public Health System and Health Department Partnerships

The areas of focus spanned the translational spectrum from creation of evidence-based guidelines (T2) to translation to communities (T4) (Table 1). Focus areas included direct research support, program evaluation, implementation research, infrastructure and expertise in data sharing, analytics, and health information technology, community needs assessments, educating or conducting interventions with community health workers (CHWs), community professional development, dissemination science, and policy setting.

**Table 1.** Partnership activities by city and translational stage

T Stage	Activity	Chicago	Los Angeles	Miami	San Francisco
T2, T3, T4	Direct research support	X	X	X	x
T2, T3, T4	Health IT, Data, Analytics	X	X	X	X
T3	Program evaluation	X	X		x
T3, T4	Implementation research	X	X	X	x
T3, T4	Dissemination	X	X		X
T4	Community needs assessments	X	X	X	X
T4	Community health workers	X	X	X	X
T4	Community professional development	X	X	X	X
T4	Policy setting	X	X	X	X

### Chicago

Chicago is the third largest city in the United States, with a population of 2.7 million. Approximately 50% of the population is non-white, with one in five people born outside of the United States and 36% speaking a language other than English at home. Twenty percent of the people in Chicago are living in poverty, which includes one in three children. The three CTSA programs in Chicago, at Northwestern University, the University of Chicago, and the University of Illinois at Chicago, formed a formal collaboration over a decade ago to advance community-engaged research across the Chicagoland region. This collaboration, *the Chicago Consortium for Community Engagement (C3*), is composed of representatives from the community engagement teams of each CTSA, the Chicago Department of Public Health (CDPH), and AllianceChicago, a nonprofit that provides research support to over 60 Federally Qualified Health Centers throughout Chicago and nationally.

In the city of Chicago, there is up to a 17-year gap in life expectancy between community areas that is closely correlated with economic status and race. The CDPH joined C3 in 2016 concurrent with the release of *Healthy Chicago 2.0*, a citywide, 4-year strategic plan to promote health equity for Chicagoʼs over 2.5 million diverse residents (CDPH HC 2.0) [10]. The report is a blueprint for establishing and implementing policies and services that prioritize residents and communities with the greatest need. CDPH and the C3 recognized that the success of Healthy Chicago 2.0 would depend, in part, on strengthening the relationship between communities and academic institutions to advance a public health research agenda.

Activities of the C3 include (1) facilitating and supporting university-based research and evaluation of CDPH-sponsored and community-based programs (four to date); (2) jointly developing mechanisms to facilitate dissemination of research opportunities and findings to community audiences; (3) aligning Clinical and Translational Science Institute (CTSI) seed funding opportunities with Healthy Chicago 2.0 priority areas; (4) facilitating collaborations with community-based organizations and community health centers; (5) collaboratively developing and delivering capacity-building workshops on community-engaged research and dissemination strategies; and (6) improving community partner and member understanding of and interest in research. Most notably, the partnership resulted in a new CDPH Office of Research and Evaluation whose lead staff position is jointly funded by the three Chicago CTSA hubs. She is currently serving on 11 CTSI research projects and center advisory boards.

The C3 meetings allow for discussion of data analytics related to The Chicago Health Atlas (ChicagoHealthAtlas) that provides public health data for the city of Chicago and aggregated community area data based on Healthy Chicago 2.0 indicators. This provides a unique opportunity to consider social determinants of health by, for example, promoting research examining medical center electronic medical record data in relation to Chicago community-level data at each CTSA program. Moreover, ongoing involvement of CDPH leadership in these discussions provides an opportunity to promote research that will inform Healthy Chicago 2025, the blueprint for Chicago healthcare policy and practices (HealthyChicago2025). Examples include recent discussions with CPDH epidemiologists to add questions regarding attitudes about research participation to the Chicago Health Survey; plans for the Chicago-based roll out of the NIH *All of Us* research initiative, and local efforts by the three Chicago CTSA programs to drive broad participation in a new local multi-institutional research portal. Lastly, the collaboration includes discussions with representatives from the Alliance for Health Equity (AllianceforHealthEquity), a collaborative of over 30 nonprofit hospitals, health departments, and community organizations, that completed a collaborative Community Health Needs Assessment for Chicago and Suburban Cook County to allow partners to collectively identify strategic priorities.

### Los Angeles

Los Angeles is the most populous county in the nation, with 10 million residents, and more people live in Los Angeles County (LAC) than in 42 states. Three quarters of the countyʼs residents are non-white, more than 30% of residents were born outside the United States, nearly one in five is below the federal poverty line, approximately one in 5 lack health insurance, and many speak a language other than English at home. The Los Angeles County Department of Health Services (LAC-DHS), the second-largest municipal health system in the United States, provides care to 700,000 patients annually through 4 hospitals, 19 comprehensive ambulatory care centers, and a network of community clinics. Many physicians serving the DHS facilities are also faculty members at the University of Southern California (USC) and University of California Los Angeles (UCLA), and DHS hospitals are training sites for physicians at USC and UCLA. The leadership of both the UCLA and USC CTSA hubs work in tandem with the DHS Chief Medical Officer to identify areas of intersection between academic research and the health system.

The parties invest resources in pilot funding for these areas of mutual interest and into two DHS-wide service cores – implementation science and clinical research informatics. Working closely with the DHS Research Oversight Board on policy and procedure development, the DHS Informatics and Analytics Core established new research informatics infrastructure, serving a county-wide clinical data warehouse and supporting 23 research pilot projects to date. The Innovation and Implementation Core facilitates multidisciplinary team science, deploys research methods that are feasible and acceptable in a safety-net health system, supports bidirectional mentoring and training, and develops new academic and public health leaders who can leverage the strengths of both systems. To date, the 18 projects supported by the Innovation and Implementation Core have affected the care provided by over 270 clinicians and outcomes of over 80,000 patients. An exemplary project supported by both cores is a teleretinal screening program that increased diabetic retinopathy screening rates from 41% to 60% and decreased ophthalmology visit wait times from 158 to 17 days [11]. To incubate and advance such multidisciplinary projects, the USC/UCLA/DHS partnership has created an intramural pilot funding program for projects that test interventions to enhance quality, efficiency, and patient-centeredness of care provided by LAC-DHS. Proposals are evaluated on these criteria, as well as promise for addressing translational gaps in healthcare delivery and health disparities, alignment with delivery system goals, and system-wide scalability. Six pilot grants have been awarded since 2016, addressing topics such as substance use disorders in the county jail, antimicrobial prophylaxis after surgery, and occupational therapy interventions for diabetes.

The Healthy Aging Initiative is an example of a collaborative effort between the UCLA and SC CTSAs, LAC-DHS, LAC Department of Public Health (LAC-DPH), the City of Los Angeles Department on Aging, California State University, and diverse community stakeholders. The initiative aims to support sustainable change in communities to allow middle-aged and older adults to stay healthy, live independently and safely, with timely, appropriate access to quality health care, social support, and services.

In addition, the Community Engagement cores at both Los Angeles (LA) hubs partner with DHS, DPH, and other LA County health departments in broad-ranging community-facing activities, including community health worker training and outreach, research education workshops based on community priorities, and peer navigation interventions.

### Miami

Home to over 6 million people, the South Florida region is the largest major metropolitan area in the State of Florida. Miami-Dade County is unique in that 69% of the county is Hispanic, 20% of persons lack health insurance, and 53% were born outside the US [12]. Since 2012, the Miami CTSI – comprising University of Miami, Jackson Memorial Health System, and Miami VA Healthcare System – has partnered with the Florida Department of Health (FLDOH) to educate and mobilize at-risk communities via the capacity building of culturally and linguistically diverse CHWs. Recognizing that CHWs serve a vital role in bridging at-risk communities and formal healthcare, in 2010, FLDOH established a Community Health Workers Taskforce (now called the Florida Community Health Worker Coalition (FLCHWC) and incorporated as a nonprofit in 2015). By 2015, the Coalition had developed a formal credentialing pathway for CHWs in the state. As a key member of the task force, the Miami CTSI provided considerable and essential input into that process. Since then, the Miami CTSI has helped develop CHW educational programs that meet training requirements on core competencies and electives for CHW certification or renewal. These programs developed in partnership with training centers, clinics, local health planning agencies, and the FLDOH are aimed at expanding the local CHW healthcare workforceʼs capacity to address health conditions related to health disparities (e.g., social determinants of health, communication skills, motivational interviewing, and oral and mental health awareness among others).

The Miami CTSI has been partnering with the FLDOH to develop condition-specific or disease-specific training in response to emergent public health concerns of local county and state health departments. In 2016, when the Zika epidemic in Latin America arrived in Florida, the Miami CTSI developed a Zika/vector-borne disease prevention training module for CHWs that were delivered in both English and Spanish across Miami/Dade County in a short timeframe. That partnership also facilitated a Zika Research Grant Initiative that awarded 12 Florida Department of Health (DOH) grants to University of Miami investigators. Totaling over $13M, the grants focused on vaccine development, new diagnostic testing or therapeutics, and dynamic change team science. Another example was in 2018 when the Miami CTSI also worked with the FLCHWC and the FLDOH in developing opioid epidemic awareness modules for CHWs. The Miami CTSI has also worked with the FLDOH around HIV workforce development. The training modules that the Miami CTSI helped develop are now offered by the FLDOH. In turn, various University of Miami CTSI sponsored research projects now have their CHWs undergo the FLDOH HIV training, which the Miami CTSI initially helped develop.

The Miami CTSI also partners with the FLDOH and the Health Council of South Florida to perform community health needs assessments and shares data with the One Florida Clinical Research Consortium (spearheaded by the University of Florida CTSA). The FLDOH is a critical stakeholder in this consortium.

### San Francisco

San Francisco is a county and city under unitary governance, with an ethnically diverse population of about 850,000 residents. It has many health sector assets, including a local public health department, a health sciences university (University of California, San Francisco [UCSF]), hospitals and health systems, and robust community-based organizations. Nonetheless, San Francisco has prominent health disparities. For example, relative to whites, hospitalization rates for diabetes are seven times higher among African Americans and twice as high among Latinos [13]. The vision of the San Francisco CTSI Community Engagement and Health Policy Program is to use an innovative Systems Based Participatory Research model which integrates community-based, practice-based, and policy research methods to advance health equity in the San Francisco Bay Area. This program strengthens the ability of academicians, the community, and Department of Public Health to conduct stakeholder engaged research through several strategies. First, the San Francisco Health Improvement Partnership (SFHIP) is a collaboration between academic, public, and community health organizations of San Francisco, an ethnically diverse city with 850,000 residents. It was formed in 2010 “to promote health equity using a novel collective impact model blending community engagement with policy change” [13]. Three backbone organizations – the San Francisco Department of Public Health, the University of California San Francisco CTSI, and the San Francisco Hospital Council – engage ethnic-based community health coalitions, schools, faith communities, and other sectors on public health initiatives. Using small seed grants from the UCSF CTSI, working groups with diverse membership develop feasible, scalable, sustainable evidence-based interventions, especially policy, and structural interventions that promote improving longer-term health outcomes. The partnership also includes community health needs assessments and a comprehensive, online data repository of local population health indicators. Results of past initiatives have been powerful. For example, the development of policy and educational interventions to reduce consumption of sugar-sweetened beverages led to new policies and legislation. These included warning labels on advertisements, a new “soda tax,” new filtered tap water stations at parks and other venues in low-income neighborhoods, and movement toward healthy beverage policies at UCSF, Kaiser Permanente, and other large hospitals. They also developed environmental solutions for reducing disparities in alcohol-related health and safety problems. As a result, they developed an alcohol outlet mapping tool that powers health research, routine blood alcohol testing in a trauma center, and influenced a new state ban on the sale of powdered alcohol, to name a few outcomes. This initiative was spearheaded by community members in neighborhoods affected by high rates of alcohol-related violence, health problems, and public nuisance activities, in collaboration with the San Francisco Police Department and other stakeholders. Using the SFHIP model, UCSF CTSI supported the development of the San Francisco Cancer Initiative, which provided science that has been used to support major community-based policy initiatives such as the banning of menthol cigarettes in San Francisco and more targeted clinical initiatives such as an effort to increase colorectal cancer screening and follow-up activities in local community health centers [14]. UCSF CTSI also has supported the San Francisco Department of Public Health in the development of its Healthy Cities Initiative, funded by Bloomberg Philanthropies, which seeks to link geocoded electronic health records data across multiple health systems with other neighborhood data to identify community-based strategies to address population health challenges across the city.

## Critical Factors and Facilitators

The participating hubs share some foundational similarities and facilitators, although their specific goals and activities are diverse. Across multiple cities, numerous factors were commonly recognized as critical to the success of the partnerships (Table 2). First and foremost, in all locales, the needs of the departments of public health and health services shaped the activities of the CTSA hubs. All partnerships were driven by the priorities of the front-line care providers, patients, and/or the public at large, reflecting the specific goals of each health department. Projects originated with problems as identified by healthcare system leaders and clinicians, public health officials, and/or community members. For example, the USC and UCLA CTSAs in Los Angeles collaborated with the LAC-DHS to use implementation science methods to develop, implement, and evaluate sustainable solutions to health system priorities. In San Francisco, the UCSF CTSI initiated the SFHIP program, but leadership and funding responsibilities were turned over to the San Francisco Department of Public Health to ensure that community stakeholders drove the agenda. The CTSAs provided value to the public health/health systems by serving as conveners; offering expertise in informatics, community health needs assessments, implementation, evaluation, and dissemination; providing education and technical support; collaborating on policy development (whether organizational or governmental policy); and leveraging relationships with community organizations.

**Table 2.** Key critical factors, facilitators, and barriers

Key Critical Factors and Facilitators
Focus on public partners’ needs and priorities Engagement by public partner champions and CTSA hub leadership Capacity for rapid response to changing health priorities Insight into community perspectives Common commitment to addressing local health disparities Seed funding aligned with public partner needs Long-lasting, trusting relationship
Key Barriers
Competing priorities Differing timelines Bureaucratic hurdles Unstable funding Political landscape Identifying appropriate academic tools for implementation

CTSA, Clinical and Translational Science Award.

Since the partnerships developed in response to the public health/health systems’ needs, their goals and activities varied. While the Miami partnership focused on developing workforce capacity, the San Francisco partnership collaborated on policy changes, and the Chicago and Los Angeles partnerships concentrated on building research infrastructure and fostering collaborative research opportunities aligned with public health and health system priorities. By using the academic tools of community-engaged research, healthcare delivery science, implementation, and dissemination research in real-world settings, the partnerships are primed for disruptive innovations in healthcare.

Second, each health department had at least one designated “champion” that helped prioritize partnership activities and advocated for the partnerships to promote tangible and immediate real-life impact. For example, the LAC-DHS Chief Medical Officer has been an enthusiastic champion for the Los Angeles partnership. He co-wrote the pilot funding opportunity request for application (RFA) and offered detailed feedback to each applicant. He was instrumental in establishing and facilitating operations and policy development for the two service cores. His perspective and influence have been critical for initiating the program, refining the program each year, and promoting the research resources available to DHS clinicians. In addition, the UCLA hub created a population health program that is co-led by the Director of Chronic Disease and Injury Prevention within the LAC Department of Public Health. In Chicago, the ongoing involvement of health department leadership with the three CTSA programs through their C3 collaboration promoted a substantial shift in C3 priorities and activities to align more closely with health department programs and practices. This ultimately led to an agreement for the CTSA programs to jointly fund a new position at the health agency to serve as a liaison between the health department and the CTSA programs, despite a city-wide hiring freeze due to statewide budget constraints. In Florida, a Centers for Disease Control and Prevention Policy, Systems and Environment Change grant to the DOH Comprehensive Cancer Control Program created a staff position that was critical to establishing consistent community engagement in developing the capacity of the Florida CHW Coalition to create a credentialing program, on-going statewide involvement in promoting CHWs, and elevate the entire south Florida regionʼs effort to incorporate CHWs in prevention practice and access to care. The rest of the state learned from Miamiʼs efforts, and Miami was strengthened with the support of the statewide coalition. The staff member was able to devote three-quarters of her time to Coalition development, which unfortunately did not continue once the grant ended.

On the academic side, CTSA principal investigators and senior administrators also dedicated significant time and effort to the initiatives beyond monetary resources. CTSA leadership collaborated with the public health/health system champions to set the vision for the initiative, viewed the partnership as a priority for their hub, and exerted the influence needed to drive initiatives forward.

Third, the CTSAs needed the capacity to respond rapidly to key stakeholders and requests. The partnerships have been particularly effective when they have been nimble and responsive to the evolving needs of the local health departments, health systems, and communities. For example, in Miami, the CTSA core trained CHWs and was primed to respond with additional disease-specific training in the setting of the Zika outbreak. The UCLA CTSA offered scientific expertise to the Department of Public Health regarding vaping and e-cigarettes.

Fourth, partnerships can ensure that the communityʼs voice is heard. By leveraging CTSAs’ Community Engagement Cores, and the longstanding partnerships between public health/healthcare systems and community organizations, the communityʼs priorities and concerns can be brought to light. In another example, the UCSF CTSA leveraged long-term trusting relationships with community groups to engage in reducing disparities in alcohol-related harms. Similarly, in Chicago, the Department of Public Health provided the CTSA representatives with an early view of a new citywide health initiative, Healthy Chicago 2025, to initiate ongoing CTSA involvement in planning and implementation. By being responsive to initiatives and priorities, CTSA goals can be harmonized with partners’ operational objectives.

Fifth, as the healthcare landscape in the United States evolves, these partnerships offer opportunities to enhance translation of evidence to practice, study the effects of various payment models, and inform policy.

Other critical factors and facilitators included a common commitment among all parties to address local health disparities; funding in the form of pilot grants tailored to the needs of the public partners, which several CTSA hubs offered, and maturity of the partnership. In Los Angeles, responsiveness to the pilot funding opportunity improved with each iteration of the funding cycle. In all cities, longer relationships increased trust among the partners.

## Lessons Learned, Barriers, Gaps, and Challenges

Numerous barriers have become evident in the infancy of these academic/public health/health system partnerships (Table 2). When evaluating the programs’ experiences, several themes emerged around challenges and the solutions employed to overcome them.

First, there are often competing priorities between the public health/health system and academic partners. All partnerships addressed this by finding areas where the public partners’ priorities aligned with academic expertise. In Miami, they developed disease-specific training in response to emergent public health concerns of local county and state health departments. In Los Angeles, the CTSA pilot funding criteria and prioritization topics were co-developed with DHS. In Chicago, seed funding projects required alignment with C3/CDPH priorities.

Second, partners’ timelines often differ substantially. The public health/healthcare system cannot adjust the pace to accommodate traditional academic endeavors. Individuals making operational decisions typically do not have the luxury of time to collect pilot data and study intervention implementation and outcomes using conventional research timelines. They are given directives to implement changes broadly and swiftly. Nevertheless, integration with academic endeavors can be achieved. One example is emphasizing underutilized research methods in implementation and improvement designed to generate both locally applicable and generalizable knowledge. Another example is embedding academicians in the public health or healthcare system, to ensure that they are involved in the design, planning, implementation, evaluation, and dissemination of initiatives. Academicians may be frustrated by hasty implementation and limitations in evaluation of outcomes, yet public health and health systems do want to base their decisions on good science. Funding cycles and grant review criteria are not consistent with business timelines and priority setting and often do not value the emerging scientific methods that are designed for learning in systems (e.g., implementation science, improvement science, design science). It is possible to undertake rigorous science that balances the competing operational needs and culture between health departments and universities when these partners focus on appropriate methods and problem-solving. In addition, researchers may have difficulty maintaining their academic credentials, gauged by grant portfolios and publication records. This is an important issue for CTSA program leadership locally and nationally, to advance changes in university tenure policies to encourage and promote health services, community-based, and community-engaged research [15]. To that end, sustained and systematic collaboration with local health departments can alleviate logistical barriers to community-engaged research to fulfill the CTSA mandate to promote research that informs policy and practice.

Third, it is critical to skillfully navigate bureaucratic hurdles when working with government entities. Several CTSAs have found it particularly effective to appoint a liaison to the public health/healthcare system. Liaisons acted as bridges between partners, drawing on expertise in multiple areas and access to resources across the partnershipʼs sites. As employees of health departments, often with dual appointments at the partnering university, liaisons understand the needs of health departments on an intimate level. With their connections and operational experience, they can act as navigators and advisors to academicians. For example, in Chicago, the new lead of Research and Evaluation at CDPH and co-chair of C3 helps researchers identify funding opportunities, disseminate research findings, and broker relationships. In addition, she serves on the CTSI community governance bodies for all three Chicago CTSIs. In Los Angeles, each of the CTSAs (UCLA and USC) appointed as their liaison an academician who practices in the DHS system. Moreover, the DHS Chief Medical Officer not only served as a supporter and champion internally but was also on the advisory committees for both USC and UCLA CTSA hubs, supporting a bidirectional strategic relationship. This is reflected in infrastructure for data services and provider workgroups promoting institutionally tailored evidence-based practices and tools [16,17]. In Miami, a trusted staff member served as the primary and long-term point of contact for communication channels and helped train a larger workforce of CHWs as an extension of the liaison model. UCSF explored creating a joint position and subsequently developed “Navigator” roles.

Agreements that make programs sustainable often have to be approved by politicians and health department leaders, and the process for obtaining approval may be complex and time consuming. A strategy for addressing the bureaucratic hurdles is to leverage the tools developed in other partnerships. We have compiled resources, including a Request for Proposals and a position description, that may be helpful to others developing similar collaborations (see Supplementary Materials). In cases where longstanding educational partnerships and agreements are in place, agreements and policies devoted to supporting translational research may build upon relationships and roles that establish faculty in leadership positions that advance research.

Fourth, unstable funding threatens the success of these partnerships. Funding is a critical factor in developing informatics and research infrastructure, workforce development, and research and evaluation. Key positions such as the liaison between the CTSI and the public health/health system should be prioritized to ensure the success of these partnerships. Strategies to address this barrier include leveraging existing resources, applying for funding from diverse sources, and being creative with resource utilization. On the other hand, mechanisms and policies for accepting funding from grants into operating budgets can also prove challenging. Three of the four LAC-DHS hospitals have an established research foundation to administer grant funding for clinician-researchers; however, these entities do not have contact with the healthcare budgeting organizations that would support resources for information technology, space, or support staff. The unpredictability of research funding is reflected in the absence of investment or awareness of procedures for accepting relatively small funds for investigator-initiated awards.

Fifth, for CTSIs collaborating with public entities, navigating a political landscape represents unique challenges. Examples include policy initiatives that could threaten corporations and well-funded industries; projects that span various public entities’ purviews (e.g., Public Health vs. Health Services vs. Mental Health); responding to politicians’ priorities; and shifting gears when administrations change. Partnerships that rely heavily on a single influential champion without associated agreements, policies, and procedures are vulnerable to leadership changes. Strong stakeholder engagement and a well-developed infrastructure are critical to ensuring the success of navigating the political sphere and sustainability.

Finally, academicians’ tools may not be well-suited to the public health systems’ needs. For example, in our Los Angeles partnership, although the UCLA and USC CTSIs had knowledge and expertise in implementation science, LAC-DHS was more interested in health delivery science, execution, operationalization, and evaluation. Rather than detailed evaluation of facilitators and barriers of implementation, they desired broad and swift implementation of interventions that reduced resource utilization while improving quality of care. Academicians have typically used an incremental approach, which often requires additional resources; whereas, LAC-DHS was more interested in disruptive approaches. We found that the best way to address the lack of alignment between the needs and the academic tools was to connect researchers with leaders in the public healthcare system early in the process of proposal development and to connect researchers with methodologists who focus on applied science in public delivery systems. Other potential solutions include expanding educational offerings for academicians, providing mentored hands-on experience, embedding researchers in public health/healthcare settings, training health department leaders in research, training community members in results dissemination, and offering incentives for cost-saving.

## Summary

Unique CTSA hub collaborations with city, county, and state health organizations are driving innovations in health service delivery and population health in four urban cities. A common element among all partnerships was the CTSA hubs’ alignment of activities with the needs of the city/county partners. Other critical factors included having designated “champions” in health departments, CTSAs’ ability to respond quickly to evolving needs, and a common commitment to addressing local health disparities. Most programs encountered similar barriers, including competing priorities, different timelines, bureaucratic hurdles, and unstable funding. The academic–public partnerships have explored numerous strategies to addressing these barriers. These partnerships offer a model for innovatively disrupting healthcare and enhancing population health.

Finding areas of common ground is key. While universities and public health/healthcare systems differ in their priorities, timelines, and *modus operandi,* successful partnerships are poised to answer some of the critical questions in health policy, including how to deliver critical services to populations in a cost-effective manner and how to address the needs of the public. Many of these challenges are not unique to partnerships between academic centers and public systems. Some of the experiences apply equally to academic medical centers that are increasingly acquiring large private healthcare organizations without an established culture of education and research. If CTSA programs are to have a substantive impact on population health, significant expansion beyond academic medical centers is needed to address the full range of social determinants of health (e.g., housing instability, concentrated poverty, chronic unemployment). Public health departments are ideal partners to consider the bidirectional relation of social determinants and health disparities [18].

## Limitations

First, this manuscript focused on partnerships between CTSAs and public entities such as Departments of Public Health or Departments of Health Services. Yet, CTSAs also have broad-ranging activities engaging communities. Second, public health and health systems have extensive collaborations with researchers and local, national, and international foundations, beyond the CTSAs. PCORnet, for example, has funded nine Clinical Research Networks; several include collaborations between universities and public health systems. Although these partnerships have been impactful, they are beyond the scope of this paper. Third, we have detailed the experiences of seven CTSAs in four large metropolitan areas. These findings and experiences may not be generalizable to other settings, particularly nonurban areas. Fourth, while we provided the experience of seven CTSAs, other CTSAs may have partnerships with their local city/county/state health departments. Rather than providing a comprehensive review of all CTSA/public health/health system partnerships, our hope was to stimulate more discussion around these partnerships.

## Future Directions

There are several ways in which collaborations among CTSA programs and public sector health departments can be optimized. First, CTSA programs can prioritize opportunities for workforce development on policy-relevant research through sponsored internships and practica for graduate students and faculty. For students, these training opportunities could be aligned with core program goals across CTSA-affiliate programs in health-related fields (e.g., medicine, public health, psychology, dentistry) to provide a community perspective and promote an awareness of public sector needs early in training. For faculty, innovative funding opportunities could be modeled on sabbatical leave of absences perhaps aligned with pilot seed funding for promising research proposals.

In addition, formal lines of communication between health departments and CTSA program leadership could be encouraged by National Center for Advancing Translational Sciences (NCATS) in RFA announcements and program reviews. Encouraging each CTSA program to have at least one public sector representative on external advisory boards could also expedite cross-channel communication. Prioritizing rapid and consistent communication could help to bridge the gap between biomedical researchers and public health/health system leadership. This is especially important for early-stage research to encourage an appreciation for community resources and needs and to anticipate common barriers to implementation research [19]. In addition, ongoing feedback across CTSA and health department leadership could provide new opportunities for bi-directional exchanges that can lead to new research opportunities as well as adaptations in ongoing research to improve community-level outcomes.

A related challenge to sustaining changes is the paucity of focus on execution and operationalization. Historically, a missing link has been failure to acknowledge and address the challenges lying between an idea or proven intervention and its implementation. Randomized trials in controlled academic settings can, at best, be considered proofs-of-concept in other settings. In addition to implementation science, a key focus should be on improvements in effective operational management and culture change. The DHS-USC-UCLA partnership has worked to close this gap by hiring, coaching, and empowering multiple academically trained physicians from both the UCLA and USC CTSI hubs by the DHS. These academically trained health services researchers have become key DHS leaders and operational managers within the clinical care delivery system. Second, the partnership has used behavioral economics as an efficient and effective culture change tool in healthcare delivery. The sustainability and retention of these types of programs and partnerships may be less financial and more cultural—a “tipping point” may require organizational dissemination and incentive alignment from the top down to cultivate operational mechanisms and durable pathways to success.

Overall, the goal is to promote research that informs health policy and to encourage health policy that is informed by research. These collaborations show that this goal is best accomplished by a strategic alliance of CTSA programs and health departments. As is evident from the examples of these four cities, new opportunities for shared data and resources emerge from ongoing discussions of shared priorities. The health departments benefit by allocation of CTSA program trainees and funding, and the CTSA programs gain valuable insight and access into community health and health system needs and resources. Ultimately, the alliances promote the overall goal of translational science to inform and improve population health.

## Supplementary material

For supplementary material accompanying this paper visit http://dx.doi.org/10.1017/cts.2020.23. click here to view supplementary material
