# Bridging the gap between research, policy, and practice: Lessons learned from academic-public partnerships in the CTSA network – CORRIGENDUM

**DOI:** 10.1017/cts.2020.495

**Published:** 2020-07-06

**Authors:** Amytis Towfighi, Allison Zumberge Orechwa, Tomás J. Aragón, Marc Atkins, Arleen F. Brown, Jen Brown, Olveen Carrasquillo, Savanna Carson, Paula Fleisher, Erika Gustafson, Deborah K. Herman, Moira Inkelas, Wylie Liu, Daniella Meeker, Tara Mehta, Doriane C. Miller, Rachelle Paul-Brutus, Michael B. Potter, Sarah S. Rittner, Brendaly Rodriguez, Dana Rusch, Anne Skinner, Hal F. Yee

In the above article by Towfighi et al. [1],Sarah S. Rittner’s name was spelled incorrectly.

Additionally, a note stating the role of the first and second authors has been added.

The original article has been updated online to reflects these changes.
