# Supplementary material for: Bridging the gap between research, policy, and practice: Lessons learned from academic–public partnerships in the CTSA network
Source: J Clin Transl Sci. 2020 Mar 10;4(3):201–8. doi: 10.1017/cts.2020.23 (PMC7348007; doi:10.1017/cts.2020.23)
Supplement: Supplementary file 1 [file S2059866120000230sup001.pdf]

Family: Planning and Urban Development
Service: Health and Welfare
Group: Medical and Social Service
Series: Human Relations

TITLE: Coordinator of Research and Evaluation

POSITION DESCRIPTION

The Coordinator of Research and Evaluation position at the Chicago Department of Public Health (CDPH) will lead a range of initiatives that support strong collaboration between academic researchers and institutions and community stakeholders to improve the health of the city of Chicago. This approach will accelerate the impact of research on the health and healthcare of communities, by cultivating diverse partnerships to integrate complementary perspectives, skills, and resources.

Working directly with CDPH and Chicago Consortium for Community Engagement (C3), this position will help engage and serve as a liaison between CDPH and key C3 constituencies to enhance city-wide collaboration and participation in research; develop and implement innovative approaches for assessing and aligning the specific research interests and resources of academic researchers and community stakeholders with Healthy Chicago 2.0 priorities; facilitate effective widespread dissemination of research findings from across Chicago to support evidence-based policymaking and practice; and leverage appropriate services provided across C3 members and partners to support their collective success. This position will build new and reinforce existing relationships with a diverse group of investigators, collaborators, CDPH and community partners. This position will also help develop a system to continuously evaluate and report progress.

ESSENTIAL DUTIES

- In collaboration with academic and community stakeholders, leads the establishment of a new CDPH Office of Research that will ensure equitable design, conduct and use of research to further health equity in Chicago.
- Develops and sustains relationships and communication with and among local Clinical and Translational Science Institutes (CTSI), CDPH, community, and other stakeholder partners. Includes online/written communication, convening and attending meetings and conferences.
- Reviews, tracks and coordinates Chicago health research, and facilitates evidence-based research findings into policy and practice
- Provides development and oversight in disseminating analytic research findings to community partners.
- Leads the development of a city-wide public health research agenda
- Directs the preparation of programmatic reports, plans, procedures and protocols based on the analysis of data collected
- Directs the identification of potential new funding sources and the completion of applications to obtain same
- Works with supervisor in establishing operating policies and procedures for the office
- Prepares reports on section’s work accomplishments
- Provide information to CTSI and CDPH senior staff for inclusion in research and grant proposals.

***NOTE**: The list of essential duties is not intended to be inclusive; there may be other duties that are essential to particular positions within the class.*

Implementation Science to Improve Patient Care

Solicitation of Letters of Intent for FY2019

Purpose: The UCLA Clinical and Translational Science Institute, the Southern California Clinical and Translational Science Institute, and the Los Angeles County Department of Health Services (LAC DHS) are partnering to develop and test interventions to enhance quality, efficiency and patient-centeredness of care provided by the LAC DHS. This solicitation requests letters of intent that describe ideas for such programs from which formal applications for funding will be selected.

Description of Pilot Grant Program: The participating CTSIs and the DHS intend to issue two pilot grants of up to $75,000 each for up to one year to support the design and small scale implementation of interventions within DHS that will achieve the goals of quality, efficiency and patient centered care. The DHS will provide additional resources and operational support for the selected projects. When completed, successful pilot projects will serve as a base for sustaining extramural funding to implement and formally evaluate the intervention(s) on a larger scale and longer timeframe within DHS. Acceptable funding mechanisms include but are not limited to NIH R01, R21, R03; PCORI, AHRQ, CDC, and CMS Center for Innovation.
<u>We encourage proposals related to productivity, implementation and improvement, and patient experience</u>. The program is designed to support projects that:

- ✓ Test a solution for a *bona fide* problem within healthcare;
- ✓ Test solution(s) that is/are aligned with the DHS's specific infrastructure, business approach and operations;
- ✓ Involve broad participation across sites and disciplines; and
- ✓ Are focused on effectiveness (i.e., whether the intervention works in real-life), as opposed to efficacy.

This program does NOT support projects that:

- × Seek to only measure or understand a problem or solution.
- × Are proposed as a theoretically good idea.
- × Involve a single discipline or single hospital or clinic.
- × Focus on efficacy (whether the intervention works under controlled conditions).

Eligibility: Awards will be made to research teams consisting of investigators from UCLA and/or USC working closely with investigators and staff members from DHS to design and conduct the pilot project. Multisite collaboration is strongly encouraged. All USC and UCLA faculty members in any series (tenured/non-tenured) including adjunct and professional research series may apply.

Workshop Opportunity: We are pleased to offer an invitation-only networking workshop and feedback session, which will be open only to select investigators who have submitted an LOI. This planning workshop will be held on **October 4, 2018 from 9am to 12pm** on the UCLA campus (exact meeting location to follow). Content experts from the funding partners at UCLA CTSI, SC CTSI and LAC DHS will be available to provide additional guidance and consultations on project ideas, and assessment of each proposed project's adherence to the Implementation Science program's aims. The workshop agenda will also include brief remarks by the sponsors and an opportunity for networking with other investigators and DHS staff. All project team members and collaborators are encouraged to attend. While attendance at the planning workshop is strongly encouraged, attendance is not a prerequisite to proceeding with submitting a full application, if invited.

Letter of Intent (LOI): Applicants should submit a brief LOI that describes:

1] The challenge in DHS health care that they propose to address [100 words max]
2] The intervention or approach that they propose to develop and pilot-test [100 words max]
3] The expertise proposed for the project, including the names and expertise of the PI and known co-investigators as well as any additional areas of expertise that will be required [no limit]
4] The expected impact of the intervention on quality, efficiency and patient centeredness of care within DHS [100 words max]

Submission: LOIs must be submitted by 5pm PST on September 21st, 2018 through this website. Questions can be addressed to Research Development (rd@sc-ctsi.org) at USC or to Deborah Herman (dkherman@mednet.ucla.edu) at UCLA.

Review: LOIs will be reviewed by representatives of the participating CTSIs and the DHS for potential to improve quality, efficiency and patient centeredness of care within the LAC DHS delivery system. Applicants should describe how the proposed research contributes to DHS delivery system goals, and its potential for/relevance to large-scale implementation within DHS and dissemination of findings more broadly. Projects that adhere to the criteria outlined above will be most strongly considered to proceed with a full proposal.

Next Steps: LOIs will be chosen for further consideration within one week of LOI submission. At that time, selected applicants will receive information on the October 4th planning workshop. Representatives from the participating CTSIs and the DHS will be available to work with applicants to assemble research teams and identify enabling resources (e.g., access to EHR) that will be needed for their proposed projects.

Summary of Timeline

- Receipt Deadline for Letters of Intent: September 21, 2018
- Planning Workshop: October 4, 2018
- Invitation for Full Proposals: November 9, 2018
- Receipt Deadline for Full Proposals: December 21, 2018
- Notification of Awardees: Early 2019

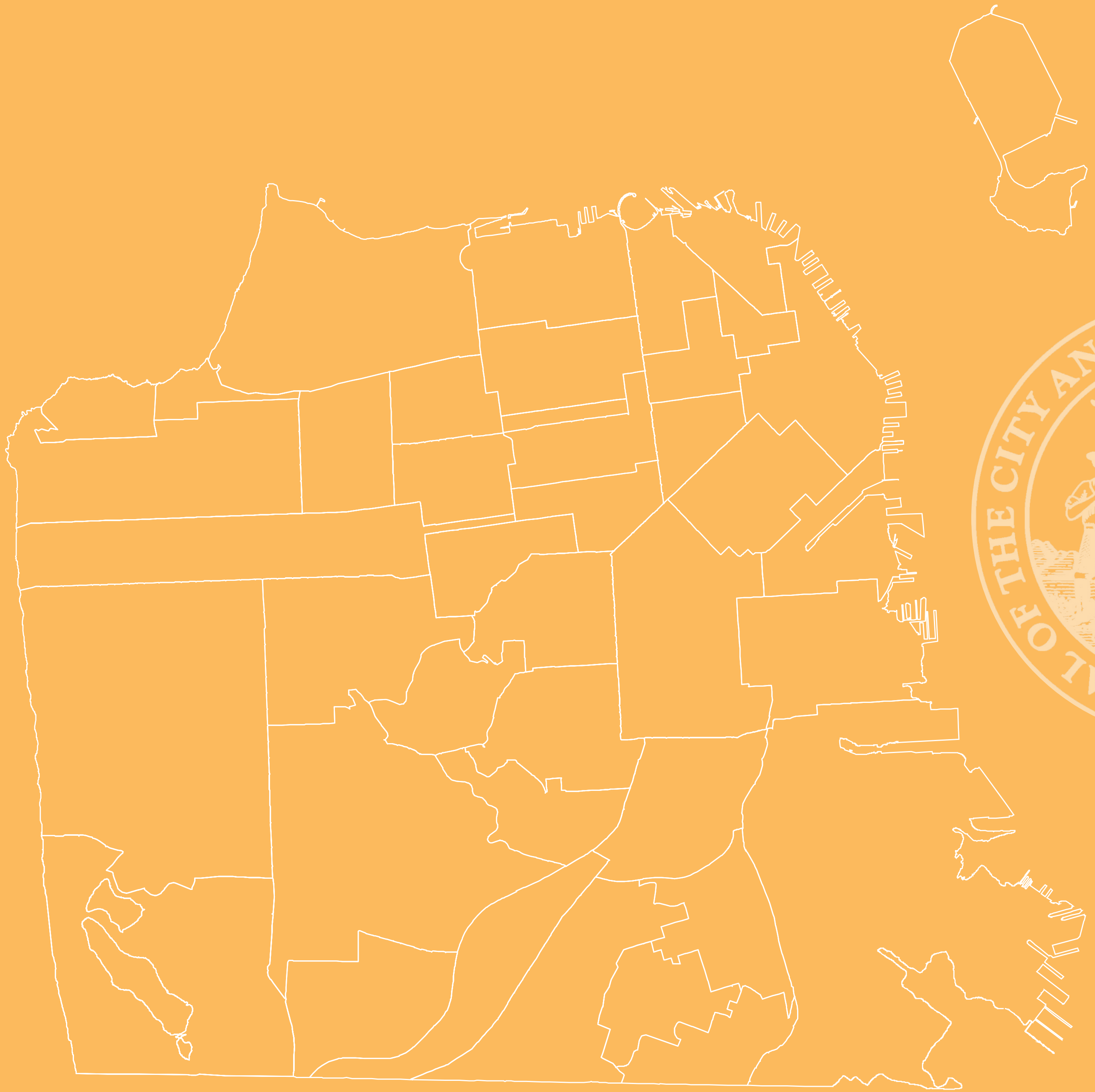

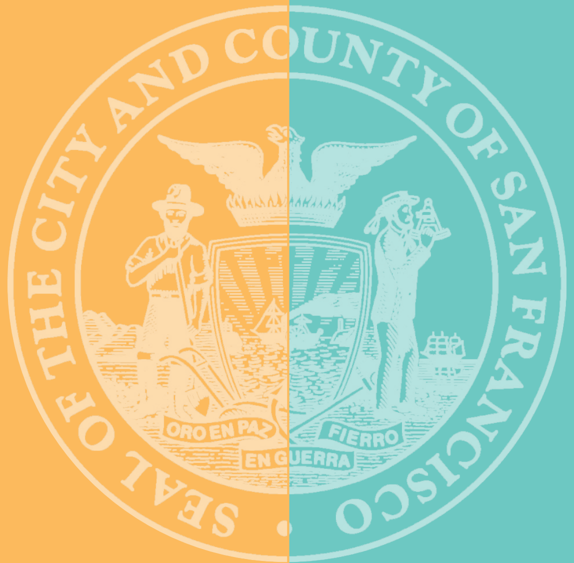

San Francisco Community Health Needs Assessment

2019

sfhip

Table of Contents

A Message from SFHIP 3

A Message from the Director of Health for the City and County of San Francisco 4

Acknowledgments 5

Executive Summary 6

THE 2019 COMMUNITY HEALTH NEEDS ASSESSMENT 8

Purpose and Collaborators 9

Approach 10

- Community Health Status Assessment 10
- Assessment of Prior Assessments 11
- Community Engagement 11
- Health Need Identification 12

San Francisco Snapshot 13

Major Findings 15

Foundational Issues 16

- Poverty 16
- Racial Health Inequities 17

Health Needs 20

- Access to Coordinated, Culturally, and Linguistically Appropriate Care and Services 20
- Food Security, Healthy Eating, and Active Living 21
- Housing Security and an end to Homelessness 24
- Safety from Violence and Trauma 26
- Social, Emotional, and Behavioral Health 30

References 35

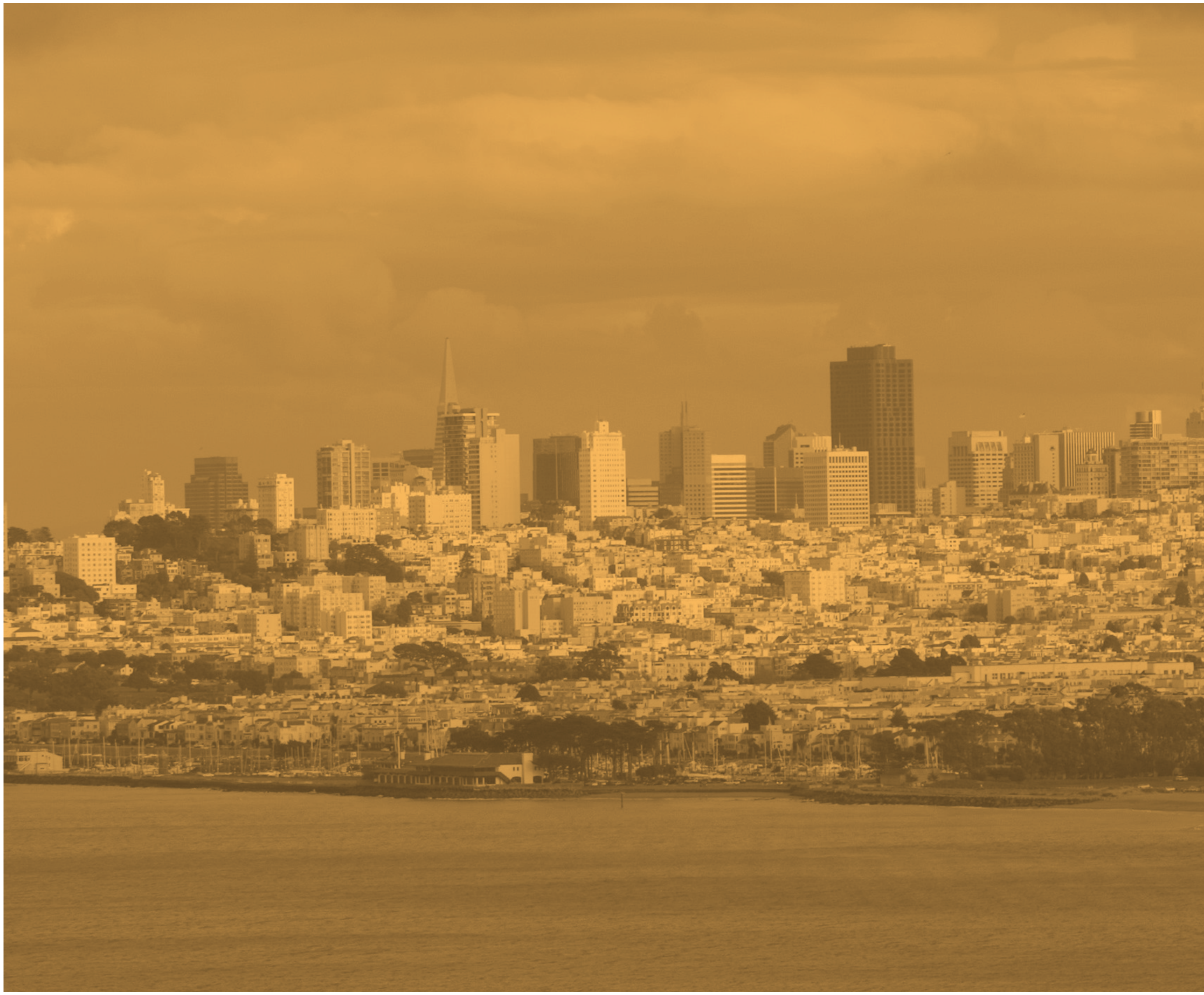

PHOTOGRAPH: PHOTOEVERYWHERE / STOCKARCH.COM

A Message from SFHIP

It is our pleasure to share with you the *2019 San Francisco Community Health Needs Assessment*. On behalf of the members of San Francisco Health Improvement Partnership (SFHIP), we hope you find this information useful in planning and responding to the needs of our community.

We would like to thank the many individuals including community residents, community-based organizations, and health care partners that contributed to this assessment. A special thank you goes out to the Community Health Needs Assessment and Impact Unit of the San Francisco Department of Public Health for their work on the data analysis and overall project management, and to the Backbone of SFHIP, staffed by the Department of Public Health, the Hospital Council, and the University of California at San Francisco, for their support for the project.

This Community Health Needs Assessment (CHNA) is part of an ongoing community health improvement process. The CHNA provides data enabling identification of priority issues affecting health and is the foundation for citywide health planning processes including the Community Health Improvement Plan, the San Francisco's Health Care Services Master Plan, the San Francisco Department of Public Health's Population Health Division's Strategic Plan, and each San Francisco non-profit hospital's Community Health Needs Assessment and Implementation Strategy.

A Community Health Improvement Plan (CHIP) is being developed as a companion to this document and will detail goals, objectives and action plans for each of the focus areas identified.

Many health needs were identified through this assessment including: access to coordinated, culturally and linguistically appropriate care and services; food security, healthy eating and active living; housing security and an end to homelessness; safety from violence and trauma; and social, emotional, and behavioral health. Additionally, poverty and racial health inequities were identified as structural and overarching issues which must be addressed to ensure a healthy San Francisco for all.

SFHIP recognizes that all San Franciscans do not have equal opportunity for good health, and we are committed to eliminating health disparities and inequities by working together across sectors to achieve health equity for all. We hope you find this assessment useful and we welcome any suggestions you may have for assisting us in improving the health of San Francisco.

SFHIP Co-Chairs
Jim Illig,
Kaiser Permanente San Francisco
Amor Santiago,
Asian and Pacific Islander Health Parity Coalition

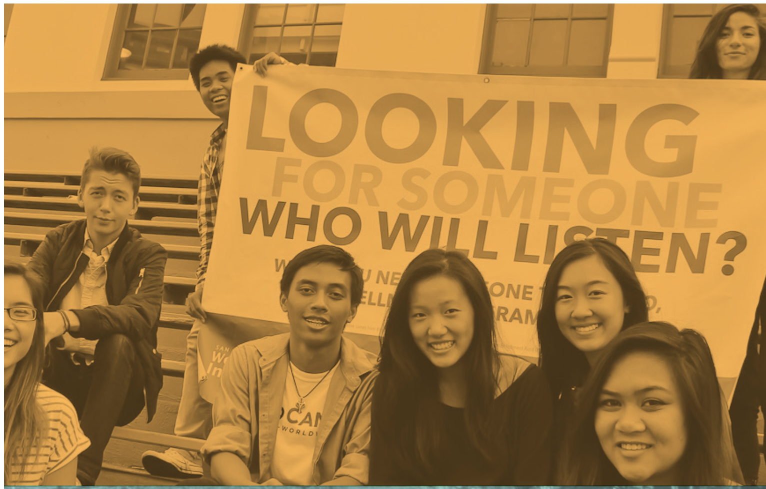

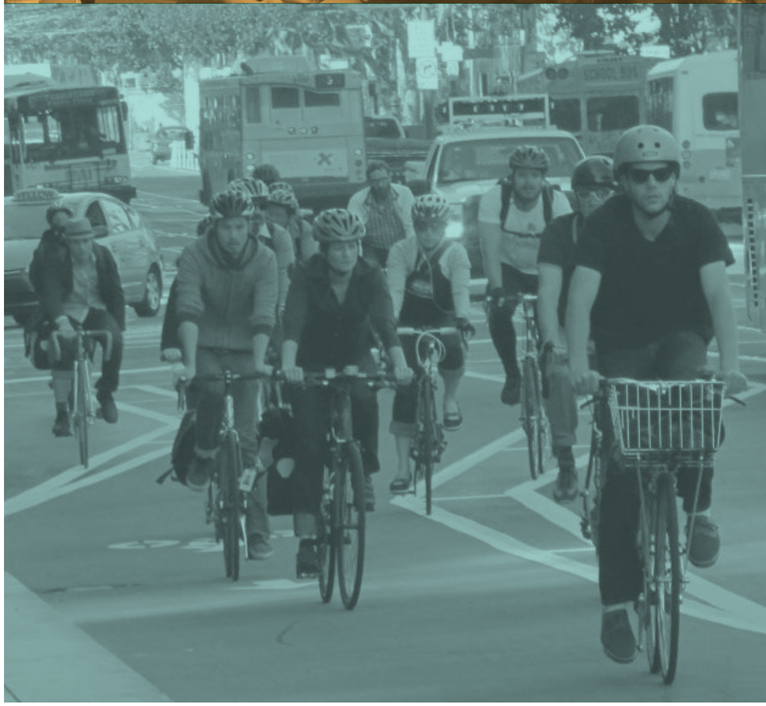

A Message from the Director of Health

I am pleased to present the 2019 Community Health Needs Assessment (CHNA) for San Francisco

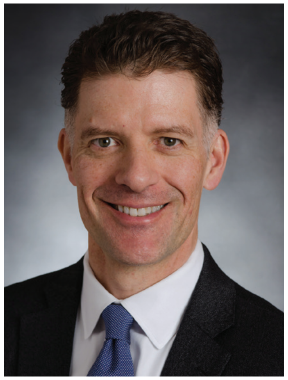

In the following pages you will find a very informative, data-rich roadmap for the continued improvement of the health of San Francisco.

The assessment takes a comprehensive look at the health of San Franciscans, through a combination of studying the social determinants of health, as well as specific health outcomes of individuals, neighborhoods and populations.

The CHNA is completed once every three years and is an important tool for informing the community about San Franciscans' health, identifying key priorities for the city and county, and gaining a better understanding of health inequities. This year, we expanded our work to provide more insights regarding homelessness, trauma and violence.

The report paints a compelling and broad picture of health and the challenges to health in San Francisco – from life expectancy, to differences in health status by neighborhoods, and racial and ethnic groups, to the renewed threat of nicotine addiction presented by e-cigarettes. Just to name a few.

The CHNA is also a key part of DPH achieving and maintaining national Public Health Accreditation, which we earned in 2017. Accreditation means that the department is meeting national standards for ensuring essential public health services and improving and protecting the health of the community.

With the CHNA, we demonstrate our ongoing collaboration with the San Francisco Health Improvement Partnership (SFHIP) that includes San Francisco hospitals, San Francisco Unified School District, University of California, San Francisco, Asian and Pacific Islander Health Parity Coalition, Chicano/Latino/Indigena Health Equity Coalition, African American Community Health Equity Council and other community members.

I commend the DPH team for this outstanding report, and extend my gratitude to the numerous community members and SFHIP partners who also contributed. Our enduring efforts are essential to fulfill our mission to protect and promote the health and well-being for all in San Francisco.

Best regards,

Grant Colfax, MD
Director of Health
San Francisco Department of Public Health
City and County of San Francisco

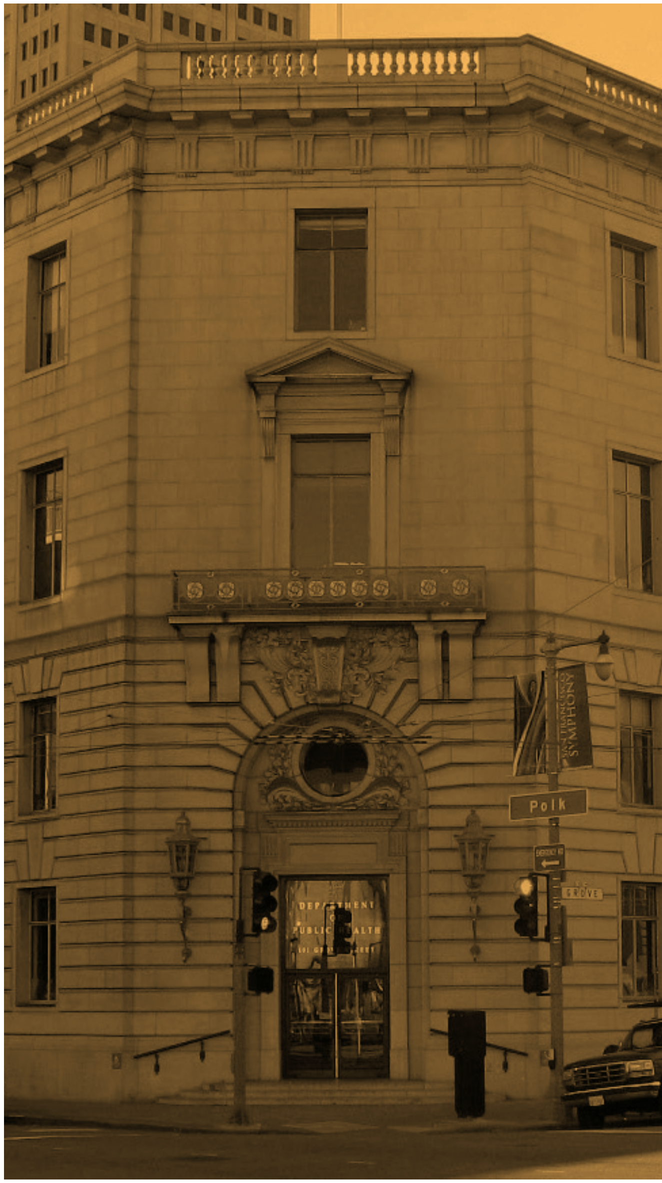

PHOTOGRAPH: MIKE HOFFMAN

Acknowledgments

San Francisco Health Improvement Partnership Steering Committee

AMOR SANTIAGO
Asian and Pacific Islander Health Parity Coalition

ANGELA SUN
Chinese Hospital

KATE WEILAND
Sutter Health California Pacific Medical Center

DEENA LAHN
San Francisco Community Clinic Consortium

ESTELA R. GARCIA
Chicano/Latino/Indigena Health Equity Coalition

FLOYD TRAMMELL
SF Interfaith Council

JENNIFER VARANO
Saint Francis Memorial Hospital

JIM ILLIG
Kaiser Permanente San Francisco

KEVIN GRUMBACH
Clinical & Translational Science Institute's Community Engagement & Health Policy Program, UCSF

KIM SHINE
San Francisco Health Services Network

ALEXANDER MITRA
St. Mary's Medical Center

MONIQUE LESARRE
African American Community Health Equity Council

SAEEDA HAFIZ
San Francisco Unified School District

SHALINI IYER
Metta Fund

TOMAS ARAGON
Population Health Division, San Francisco Dept of Public Health

Community Health Needs Assessment Leadership and Major Contributors

Ameerah Thomas, San Francisco Department of Public Health

Ayanna Bennett, San Francisco Department of Public Health

Jason Xu, San Francisco Department of Public Health

Jodi Stookey, San Francisco Department of Public Health

Lauren Swain, University of San Francisco

Matt Wolff, San Francisco Department of Public Health

Max Gara, San Francisco Department of Public Health

Meg Wall-Shui, San Francisco Department of Public Health

Michelle Kirian, San Francisco Department of Public Health

Paula Fleisher, Department University of California at San Francisco

Paula Jones, San Francisco Department of Public Health

Priscilla Chu, San Francisco Department of Public Health

Victor Kong, San Francisco Department of Public Health

Wylie Liu, University of California at San Francisco

Hospital Council of Northern & Central California

David Serrano Sewell
Laura Goria

University of California at San Francisco

Kaya Balke
Mollie Belinski
Nicholas Evans
Laura Fejerman
Stan Glanz
Robert Hiatte
Carmela Lamonaco
James Rouse Iñiguez
Alex Rutherford
Ma Somsouk
Roberto Vargas
Priyanica Vyas
Susan Wang
Erica Wong
Aileen Xu

San Francisco Department of Public Health

Nora Anderson
Laura Braining-Rodriguez
Katie Burk
Curtis Chan
Carol Chapman
Shrimati Data
Derek Smith
Cristy Dieterich
Patricia Erwin
Margaret Fisher
Patrick Fosdahl
Joanna Fraguli
Jenna Gaarde
Dale Gluth
Christina Goette
Mary Hansel
Ling Hsu
Brandon Ivory
Karen Kohn
Mia Lei
Dedriana Lomaz
Zea Malawa
Devan Morris
Shivaun Nestor
Rita Nguyen
Trang Nguyen
Israel Nieves
Amy Nishimura
Melissa Ongpin
Prasanthi Patel
Sneha Patil
Susan Philip
Sharon Pipkin
Uzziel Prado
Priti Rane
Chris Rowe
Veronica Shepard
Maryna Spiegel
Marianna Szeto
Mimi Tam
Ana Validzic
Megan Wier
Tiffany Yim
Janine Young

Community Partners

Mory Chhom, Vietnamese Youth Development Center

Joanne Compean, Homeless Prenatal Program

Dara Geckler, Facente Consulting

Debbie Lerman, San Francisco Human Services Network

Ellen Moffatt, San Francisco Office of the Chief Medical Examiner

Stefan Nilsen, University of San Francisco

Martha Ryan, Homeless Prenatal Program

Karma Smart, Rafiki Coalition for Health and Wellness

Pedro Vidal Torres, Center for Open Recovery

Karen Zeigler, San Francisco Office of the Chief Medical Examiner

Executive Summary

Welcome to the **Community Health Needs Assessment** (CHNA). The CHNA takes a broad view of health conditions and status in San Francisco. In addition to providing local disease and death rates, this CHNA also provides data and information on social determinants of health—social structures and economic systems which include the social environment, physical environment, health services, and structural and societal factors.

The CHNA involves four steps:

- Community health status assessment
- Assessment of prior assessments
- Community engagement
- Health need identification and prioritization

The CHNA is the foundation for each San Francisco non-profit hospital's Community Health Needs Assessment and is one of the requirements for Public Health Accreditation. While the CHNA informs large-scale city planning processes such as San Francisco's Health Care Services Master Plan, the intent of this document is to inform the work of all organizations, teams and projects that impact the people of San Francisco. Gaining an understanding of why health outcomes exist here in San Francisco can help gear our efforts towards addressing root causes and developing better interventions, policies and infrastructure. SFDPH's mission is to protect and promote the health of all San Franciscans, and we all have a contribution to achieving this goal, no matter the scale or scope of our work.

Overall, the CHNA finds that health has improved in San Francisco:

- More San Franciscans have access to health care.
- The estimated rate of new HIV infection in San Francisco continues to decrease.
- Life expectancy increased for all San Franciscans with the biggest gains seen by Black/African Americans.
- Mortality rates due to lung, colon, and breast cancers and influenza and pneumonia continue to decline.
- The availability of tobacco products has decreased. At 11%, rates of smoking are lower than the Healthy People 2020 goal of 12%.
- 2017 had the lowest number of traffic-related fatalities since record keeping began in 1915.

The CHNA identifies two foundational issues contributing to local health needs:

- Racial health inequities
- Poverty

The CHNA identifies five health needs that heavily impact disease and death in San Francisco:

- Access to coordinated, culturally and linguistically appropriate care and services
- Food security, healthy eating and active living
- Housing security and an end to homelessness
- Safety from violence and trauma
- Social, emotional, and behavioral health

Foundational Issues

Racial Health Inequities

Health inequities are avoidable differences in health outcomes between population groups. Health inequities result from both the actions of individuals (health behaviors, biased treatment by health professionals), and from the structural and institutional behaviors that confer health opportunities or burdens based on status. For example, the uneven distribution of wealth and resources determines the level of health those getting the least of these resources can achieve. Pages 17–19 include data on a few improvements to health and determinants of health and point to where more work needs to be done to address the structural and institutional racism in San Francisco. Additional data on health inequities are found throughout the Community Health Data pages.

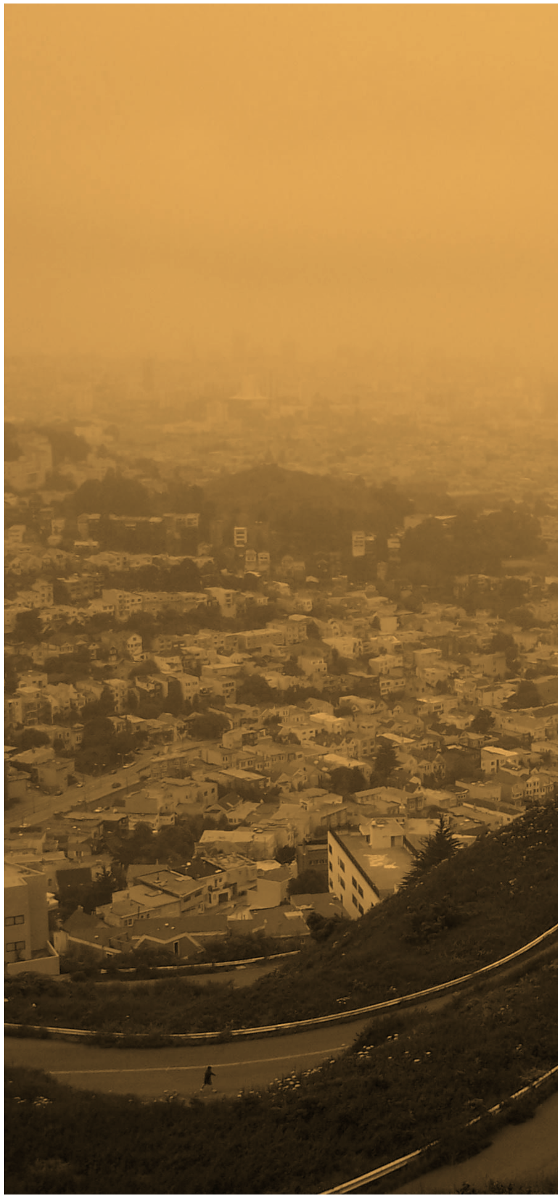

Executive Summary

Poverty

Enough income generally confers access to resources that promote health — like good schools, health care, healthy food, safe neighborhoods, and time for self-care — and the ability to avoid health hazards — like air pollution and poor quality housing conditions. Page 16 focuses on the economic barriers to health that many San Franciscans face. Find additional data on economics and health in the Economic Environment data page.

Health Needs

Access to Coordinated, Culturally and Linguistically Appropriate Care and Services

San Francisco continued to see gains in access to health care with 10,000 fewer residents uninsured in 2017 than in 2015. Of the estimated 31,500 uninsured residents, 15,373 have health care access through Healthy San Francisco or Healthy Kids. Approximately 2% of residents remain without access. Having insurance or an access program is only the first step; however, as true access to services is influenced by location, affordability, hours of operation, and cultural and linguistic appropriateness of health care services. Page 20 presents San Francisco statistics on health care use, barriers to use, and consequences of not having access to quality care. Additional information on health care quality and access is located in the Health Care Access and Quality data page.

Food Security, Healthy Eating and Active Living

Inadequate nutrition and a lack of physical activity contribute to 9 of the leading 15 causes of premature death in San Francisco — heart failure, stroke, hypertension, diabetes, prostate cancer, colon cancer, Alzheimer's, breast cancer, and lung cancer. Studies have shown that just 2.5 hours of moderate intensity physical activity each week is associated with a gain of approximately three years of life. Data on physical activity and healthy eating and barriers to each are presented on pages 21–23. Additional data are available in the Physical Activity, Transportation, Crime and Safety, Overweight and Obesity, and Nutrition data pages.

Housing Security and an End to Homelessness

Housing is a key social determinant of health.[1] Housing stability, quality, safety, and affordability all have very direct and significant impacts on individual and community health. Much of California, and especially the Bay Area, is currently experiencing an acute shortage in housing, leading to unaffordable housing costs, overcrowding, homelessness and other associated negative health impacts. Between 2011 and 2015, the Bay Area added 501,000 new jobs — but only 65,000 new homes. An estimated 24,000 people in San Francisco live in crowded conditions and about 7,500 homeless persons were counted in San Francisco. Pages 24 – 25 provide an overview of the housing stressors in San Francisco. Additional information on housing and health is found in the Housing data page.

Safety from Violence and Trauma

Violence not only leads to serious mental, physical and emotional injuries and, potentially, death for the victim, but also negatively impacts the family and friends of the victim and their community. Persons of color are more likely to be victims of violence, to live in neighborhoods not perceived to be safe and to receive inequitable treatment through the criminal justice system. Pages 26 – 29 focus on violence and trauma, their determinants and health impacts in San Francisco. Additional data on violence and trauma in the City are presented in the Crime and Safety data page.

Social, Emotional, and Behavioral Health

Mental health is an important part of community health. In San Francisco the number of hospitalizations among adults due to major depression exceed that of asthma or hypertension. Presence of mental illness can adversely impact the ability to perform across various facets of life — work, home, social settings. It also impacts the families, caregivers, and communities of those affected. Substance abuse including drugs, alcohol and tobacco, contributes to 14 of the top causes of premature death in the City — lung cancer, Chronic Obstructuve Pulmonary Disease, HIV, drug overdose, assault, suicide, breast cancer, heart failure, stroke, hypertensive heart disease, colon cancer, liver cancer, prostate cancer, and Alzheimer's. Pages 30 – 34 focus on psychological distress, major depression, and substance abuse in San Francisco. Find additional data on social, emotional and behavioral health in the City in the Mental Health, Substance Abuse, and Tobacco Use and Exposure pages.

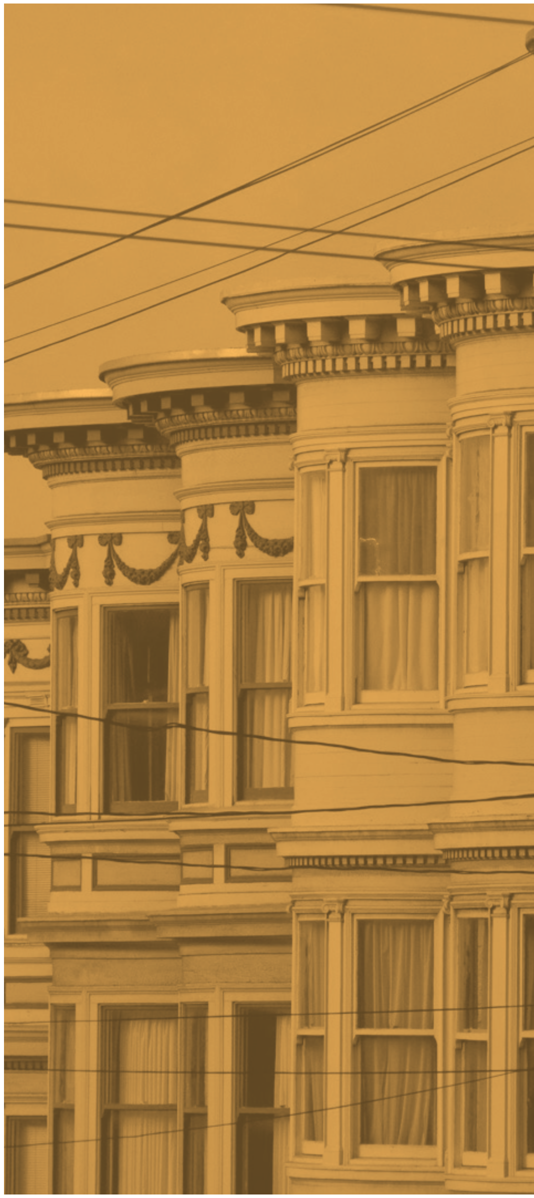

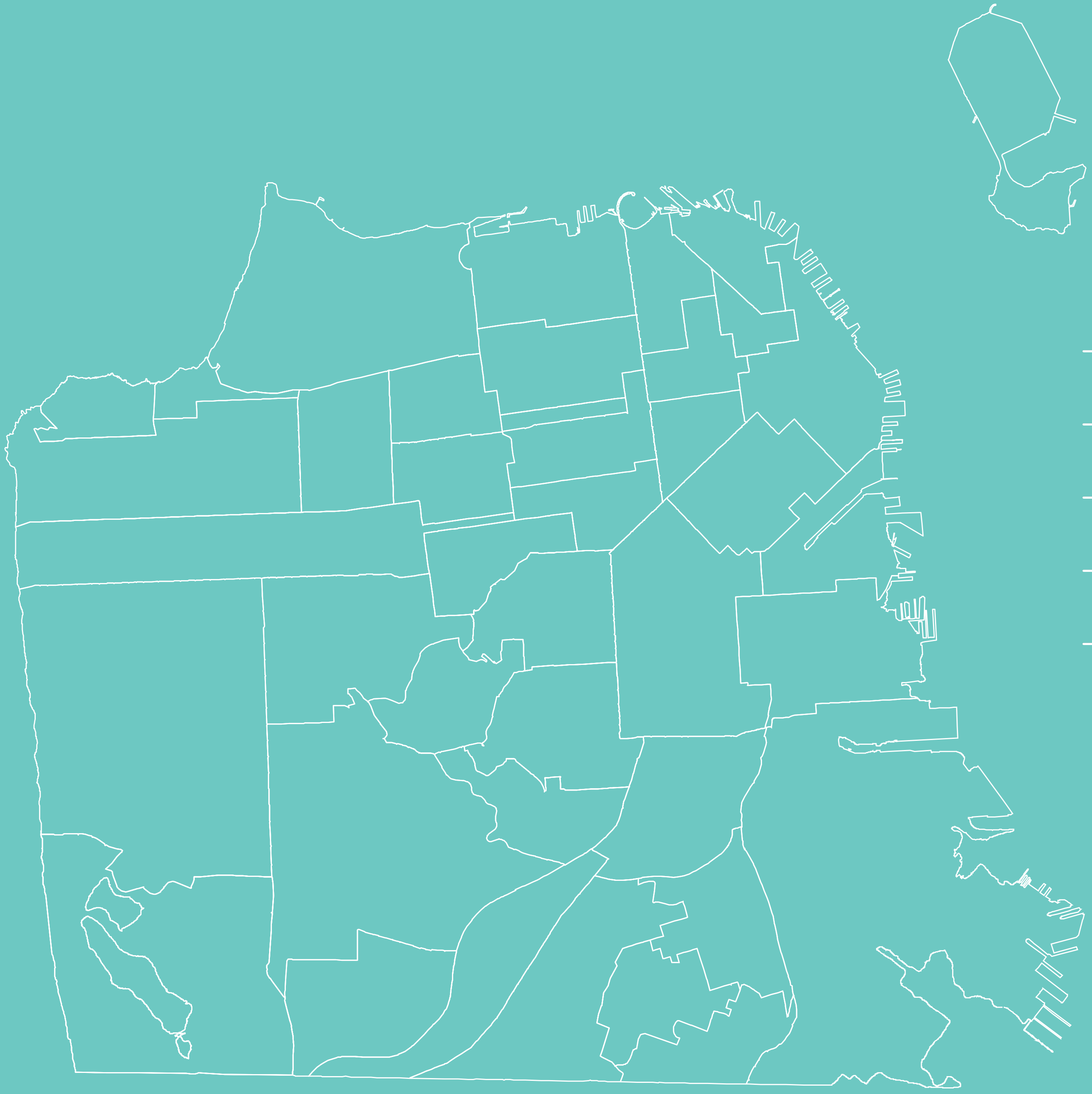

The 2019 Community Health Needs Assessment

Purpose and Collaborators 9

Approach 10

San Francisco Snapshot 13

Major Findings 15

References 35

Purpose & Collaborators

The **2019 Community Health Needs Assessment** (CHNA) takes a comprehensive look at the health of San Francisco residents by presenting data on demographics, socioeconomic characteristics, quality of life, behavioral factors, the built environment, morbidity and mortality, and other determinants of health status.

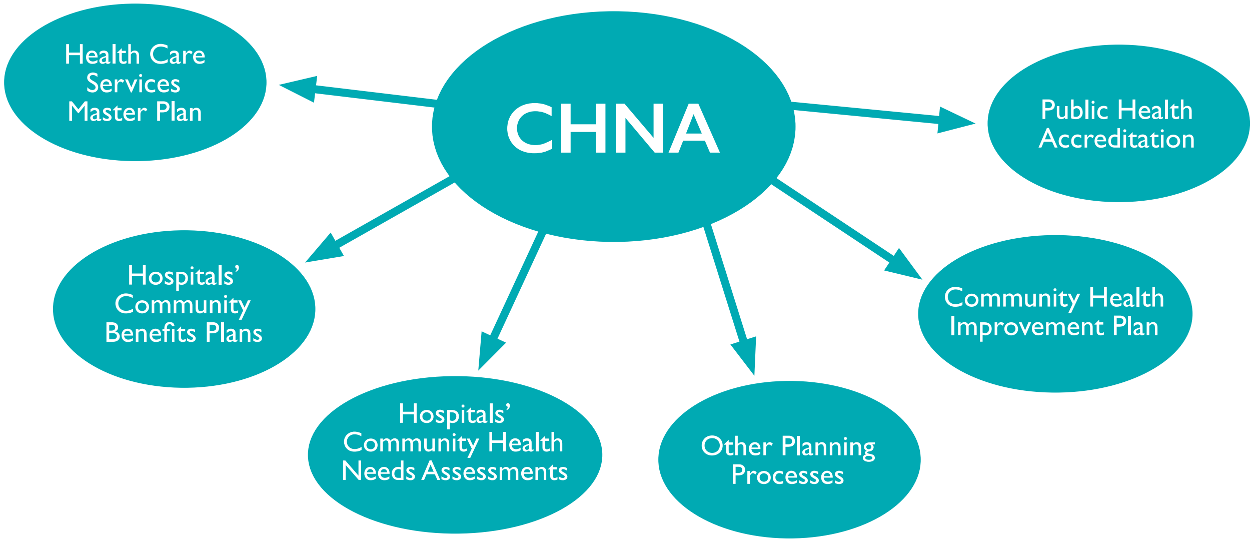

The CHNA is the foundation for each of San Francisco's non-profit hospitals' Community Health Needs Assessments and is one of the requirements for Public Health Accreditation, which includes: a CHNA, a community health improvement plan, and a strategic plan for population health. The CHNA also informs city planning processes such as San Francisco's Health Care Services Master Plan.

While the CHNA informs large-scale city planning processes, the intent of this document is to inform the work of all organizations, teams and projects that impact the people of San Francisco. Gaining an understanding of why health outcomes exist here in San Francisco can help gear our efforts towards addressing root causes and developing better interventions, policies and infrastructure.

The San Francisco Health Improvement Partnership (SFHIP) guided CHNA development. SFHIP is a collaborative body whose mission is to embrace collective impact and to improve community health and wellness in San Francisco. Membership in SFHIP includes the San Francisco Department of Public Health, San Francisco's non-profit hospitals, the Clinical and Translational Science Institute's Community Engagement and Health Policy Program at UCSF, the San Francisco Unified School District, The Office of the Mayor, community representatives from the Asian and Pacific Islander Health Parity Coalition, Human Service Network, Chicano/Latino/Indigena Health Equity Coalition, and African American Community Health Council, Community Clinic Consortium, Faith based and philanthropic partners. SFHIP completes a CHNA once every three years.

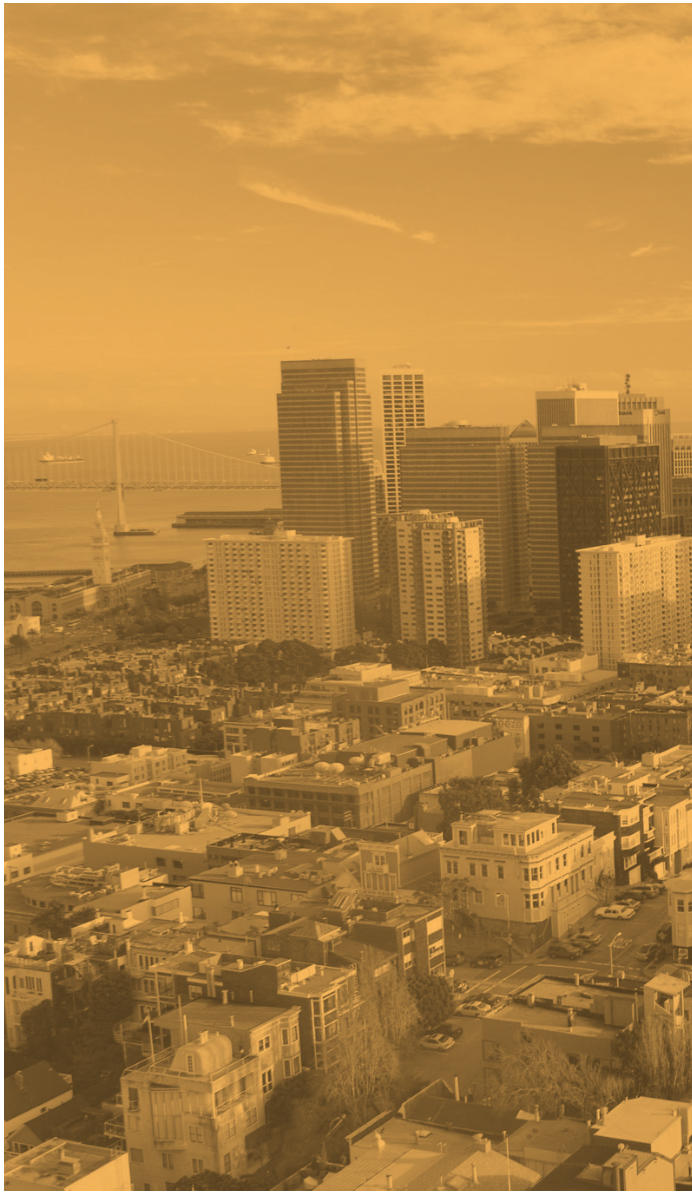

Approach

The Community Health Needs Assessment takes a life course approach when exploring and presenting the health needs of San Franciscans.

A life course approach considers one's lived experience and health throughout the lifespan, within the context of their history, environment, family, community, society, and culture. Certain events and exposures (i.e. trauma, racism, poverty, environmental factors, etc.) during sensitive time periods in early life can have long-term impacts on development and health.[1]

In addition to impacting one's own future health status, early life experiences can have intergenerational health outcomes. One's wellness during the prenatal or pregnancy periods impacts the health of one's children. Investing in pregnancy, early childhood, and family wellbeing through policies, interventions and systems can support our society and address the root causes of health inequities.

Data Collection

The CHNA collected information on the health of San Franciscans via three methods:

- Community Health Status Assessment
- Assessment of Prior Assessments, and
- Community Engagement.

Through review of the information provided by these sources, SFHIP identified San Francisco's health needs. Additionally, following the health needs assessment a Community Asset Assessment was completed.

Community Health Status Assessment

Health is a state of complete physical, mental, and social well-being and not merely the absence of disease or infirmity.[2] While biology, genetics, and access to medical services are largely understood to play an important role in health, social-economic and physical environmental conditions are now known to be major, if not primary, drivers of health.[2-4] These conditions are known as the Social Determinants of Health and are shaped by the distribution of money, power, and resources throughout local communities, nations, and the world.[5]

SAN FRANCISCO FRAMEWORK FOR ASSESSING POPULATION HEALTH AND EQUITY

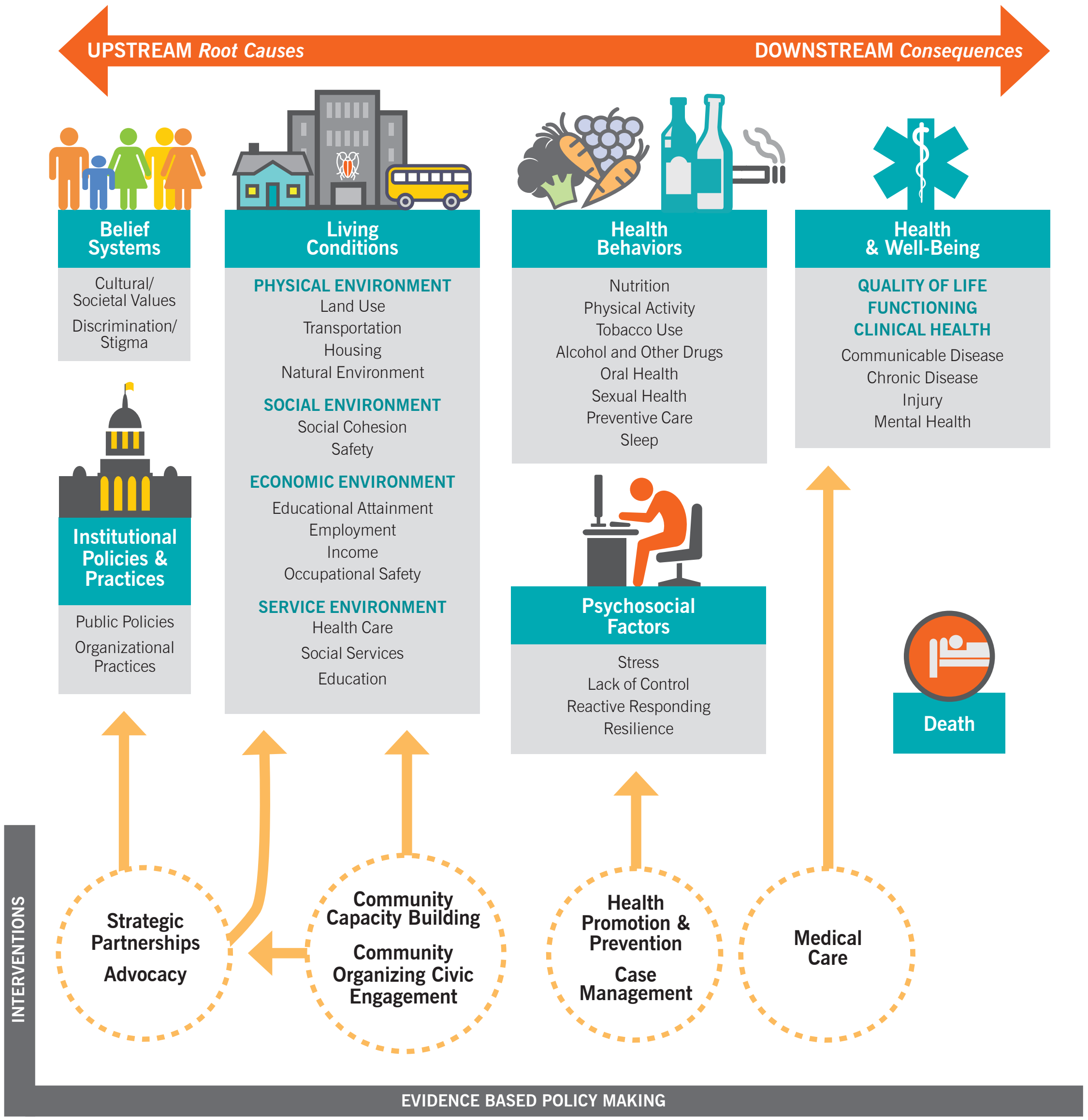

Approach

Recognizing the essential role social determinants of health play in the health of San Franciscans, the Community Health Status Assessment examined population level health determinant and outcome variables. We used the **San Francisco Framework for Assessing Population Health and Equity**, which is a modified version of the Public Health Framework for Reducing Health Inequities published by the Bay Area Regional Health Inequities Initiative to guide variable selection.[3] We ranked and selected available variables based on the Results Based Accountability criteria for indicator selection — communication power (ability to communicate to broad and diverse audiences), proxy power (says something of central significance), and data power (available regularly and reliably), as well as the ability to examine health inequities and current use by stakeholders. Furthermore, we hosted meetings throughout 2017 to gather feedback on indicators from experts and community representatives. In all, 171 variables were analyzed. We present the results from all analyses in 30 Community Health Data pages.

To reveal health disparities, the Community Health Status Assessment analyzed data by age, race/ethnicity, poverty, place, and more. However, available data do not permit analyses for all groups which are known to experience health inequities including Native Americans, people who identify as LGBTQ, transgender persons and persons with disabilities.

Assessment of Prior Assessments

San Francisco's community-based organizations, healthcare service providers, public agencies and task forces conduct health needs assessments and publish reports of their activities for planning and evaluation purposes and to be accountable to those they serve. Our aim in conducting an assessment of these assessments and reports is to augment what we know from routinely collected secondary health data and primary data collection through CHNA community engagement activities. We hope thereby to gain a better understanding of which communities/populations in San Francisco have been engaged in health needs assessment activities; what topics are of concern and interest to these communities/populations; and learn about promising and effective approaches to eliciting and addressing these concerns. We included both needs assessments and service reports in our definition of "assessments" for this assessment.

Beginning in January 2017, CHNA administrative leads from the SF Department of Public Health and UCSF and a small Working Group consisting of members of San Francisco's three health equity/parity coalitions, UCSF health professions students, and UCSF Clinical and Translational Research staff began conducting online searches for published assessment reports for the 2019 CHNA.

For this assessment, the San Francisco Framework for Assessing Population Health and Equity was used to define "Root Causes" that reflect social determinants. Additionally, the Working Group decided to add incarceration, experience with law enforcement, and community development/ investment to the framework.

Further details on methods used and findings are presented in the Assessment of Prior Assessments page.

Community Engagement

The goals of the community engagement component of the CHNA are to:

- Identify San Franciscan's health priorities, especially those of vulnerable populations
- Obtain data on populations and issues for which we have little quantitative data
- Build relationships between the community and SFHIP
- Meet the regulatory requirements including the IRS rules for Charitable 501c3 Charitable Hospitals, Public Health Accreditation Board requirements for the San Francisco Health Department, and the San Francisco's Planning Code requirements for a Health Care Service Master Plan

The 2019 CHNA includes **4 categories of focus groups**: SFHIP key informant group interview, Equity Coalition focus groups, food insecure pregnant women focus groups, and Kaiser focus groups.

SFHIP Key Informant Group Interview

One focus group was comprised of SFHIP members who are all subject matter experts. Two series of questions were asked, "What are the healthiest characteristics of this community? What supports people to live healthier lives?" and "What are the biggest health issues and/or conditions your community struggles with? What do you think creates those issues?".

Equity Coalition focus groups

Three focus groups were conducted with each of the three health equity coalitions in San Francisco: The Chicano / Latino / Indigena Health Equity Coalition, The Asian Pacific Islander Healthy Parity Coalition, and The African American Health Equity Coalition. Using the Technology of Participation (ToP) Consensus Method, the question posed to each focus group was, "What actions can we take to improve health?"

Food Insecure Pregnant Women focus groups

The Homeless Prenatal Program held four focus groups with women who experienced food insecurity while pregnant. Each focus group focused on a different group of women: Spanish, Chinese, multi-ethnic English speakers, and African American. The question to respond to was, "What actions can we take to improve your food needs?"

Kaiser led focus groups

Kaiser conducted four focus groups, one each with Kaiser Permanente leadership, Kaiser Permanente staff, Spanish-speaking parents on youth healthy eating and active living, and homeless and/or HIV positive youth.

Further details on the methods and findings are available in the Community Engagement page.

Approach

Health Need Identification

To identify the most significant health needs in San Francisco the SFHIP steering committee met on October 18th, 2018. Participants identified health needs through a multistep process. First participants reviewed data and information from the Community Health Status Assessment, the Assessment of Prior Assessments, and the Community Engagement, as well as the health priorities from the 2016 Community Health Improvement Plan. Then, using the Technology of Participation approach to consensus development, participants engaged in a focused discussion about the data. Finally, participants developed consensus on the health needs. (**Figure A**) Throughout the process needs were screened using pre-established criteria (**Figure B**). This process yielded two foundational issues and five health needs.

Foundational issues are needs which affect health at every level and must be addressed to improve health in San Francisco.

The two foundational issues identified were:

- Poverty
- Racial health inequities

The five health needs identified were:

- Access to coordinated, culturally and linguistically appropriate care and services
- Food security, healthy eating, and active living
- Housing security and an end to homelessness
- Safety from violence and trauma
- Social, emotional, and behavioral health

Data describing part of each of the foundational issues and health needs are located in the Major Findings pages and in the various Community Health Data pages.

Figure A: Consensus development steps

1	Individually listing of top health needs
2	Small group discussions on the top health needs to identify similarities and differences
3	Sharing all the health needs identified by the individuals
4	Clustering the similar health needs into themes
5	Determining a name for the theme, which is the health need
6	Comparing and discussing new needs with those from 2012 Community Health Improvement Plan

Figure B: Health need screening criteria

Health need is confirmed by more than one indicator and/or data source
Need performs poorly against a defined benchmark(s)

Health needs include health outcomes of morbidity and mortality as well as behavioral, environmental, clinical care, social and economic factors that impact health and well-being.

Community Assets Assessment

To identify the community's resources available to address identified health needs, the San Francisco Department of Public Health reviewed data collected during the Community Engagement activities described above.

Questions asked of the participants relevant to the Community Asset Assessment included, "What are the strengths, resources, and assets of your community?", "What are the barriers that contribute to health issues for your community?", "What are the strengths and resources you and your family have to support your food needs?", and "What makes it hard to address you and your family's food needs?"

Further details on the methods and findings are available in the Community Assets Assessment and Community Engagement pages.

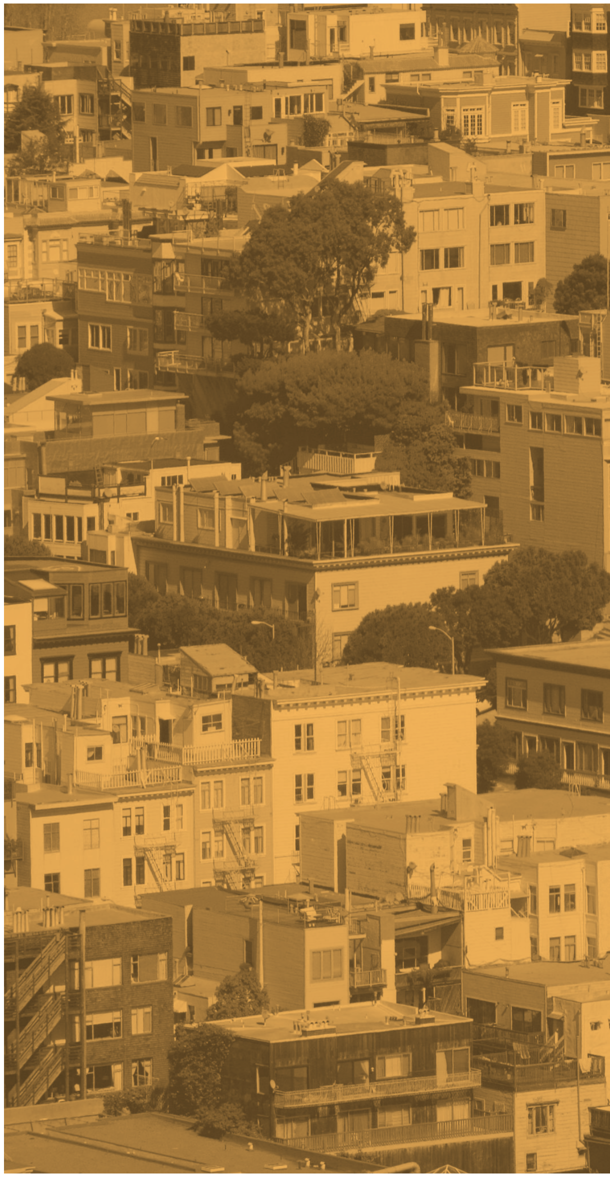

PHOTOGRAPHY: PHOTOEVERYWHERE / STOCKARCH.COM

San Francisco Snapshot

Population Growth

San Francisco is the cultural and commercial center of the Bay Area and is the only consolidated city and county jurisdiction in California. At roughly 47 square miles, it is the smallest county in the state, but is the most densely populated large city in California (with a population density of 17,352 residents per square mile) and the second most densely populated major city in the US, after New York City.[1]

Between 2011 and 2018 the population in San Francisco grew by almost 8 percent to 888,817 outpacing population growth in California (6 percent).[2] By 2030, San Francisco's population is expected to total more than 980,000.

Population by age group as a percentage of the total population projections, SF, 2010–30

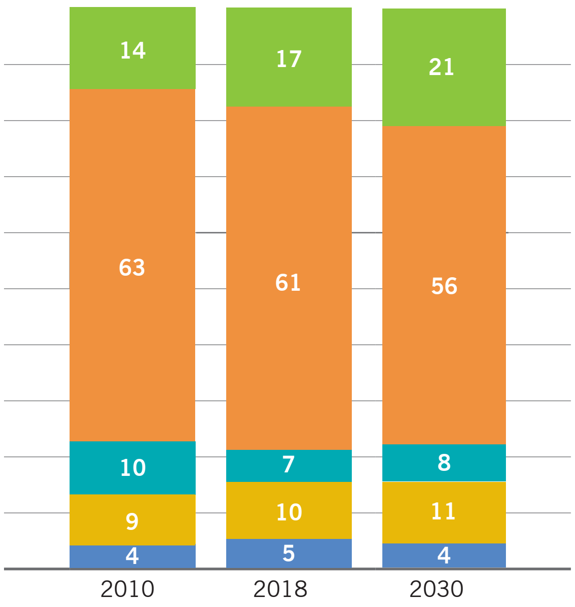

Groups by age range in years: Seniors (65+), Working Adults (25–64), College Age (18–25), School Age (5–17), Preschool Age (0–4).

An Aging Population

The proportion of San Francisco's population that is 65 years and older is expected to increase from 17 percent in 2018 to 21% in 2030; persons 75 and over will make up about 11% of the population.[2] At the same time, it is estimated that the proportion of working age residents (25 to 64 years old) will decrease from 61 percent in 2018 to 56 percent in 2030. This shift could have implications for the provision of social services.

Ethnic Shifts

Population growth is expected for all races and ethnicities except for Black/African Americans who are projected to drop from 4.9 percent of the population in 2018 to 4 percent in 2030.[3] Asians and Whites will remain the most populous groups and will grow as a percentage of the overall population. Population growth is expected to be lower for Latinx and Pacific Islanders and Latinx are expected to drop from 15.1 to 14.8 percent of the population.

Currently, 35 percent of San Francisco's population is foreign born and 20 percent of residents speak a language other than English at home and speak English less than "very well."[1,4] The majority of the foreign born population comes from Asia (65 percent), while 18 percent were born in Latin America, making Chinese (Mandarin, Cantonese, and other) (43 percent) and Spanish (26 percent) the most common non-English languages spoken in the City.[4]

Families and Children

Although San Francisco has a relatively small proportion of households with children (19 percent) compared to the state overall (34 percent), the number of school-aged children is projected to rise.[2,5] As of 2017, San Francisco is home to 67,740 families with children, 26 percent of which are headed by single parents.[5] There are approximately 132,330 children under the age of 18.[2] The number of school-aged children is projected to rise by 24 percent by 2030.[2] The neighborhoods with the greatest proportion of households with children are: Seacliff, Bayview Hunters Point, Visitacion Valley, Outer Mission, Excelsior, Treasure Island, and Portola (all over 30%).[1]

Ethnic composition by percentage of population, SF, 2010–30

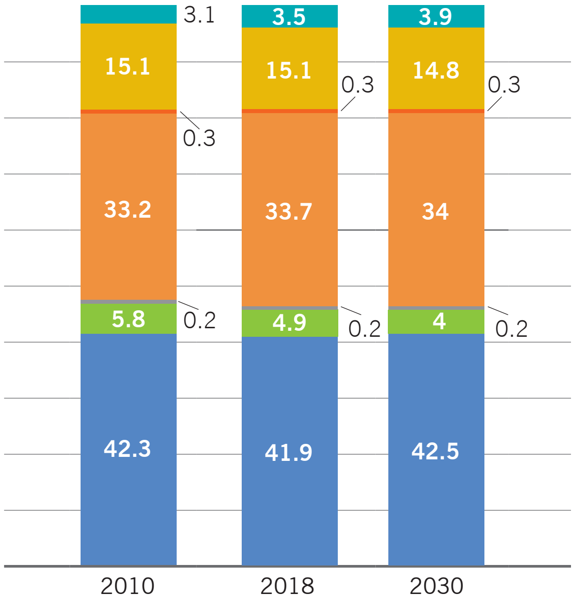

Multi-ethnic, Latinx, Pacific Islander, Asian, Native American, Black/African American, White.

sfhip

San Francisco Neighborhoods and Zip Codes, 2014

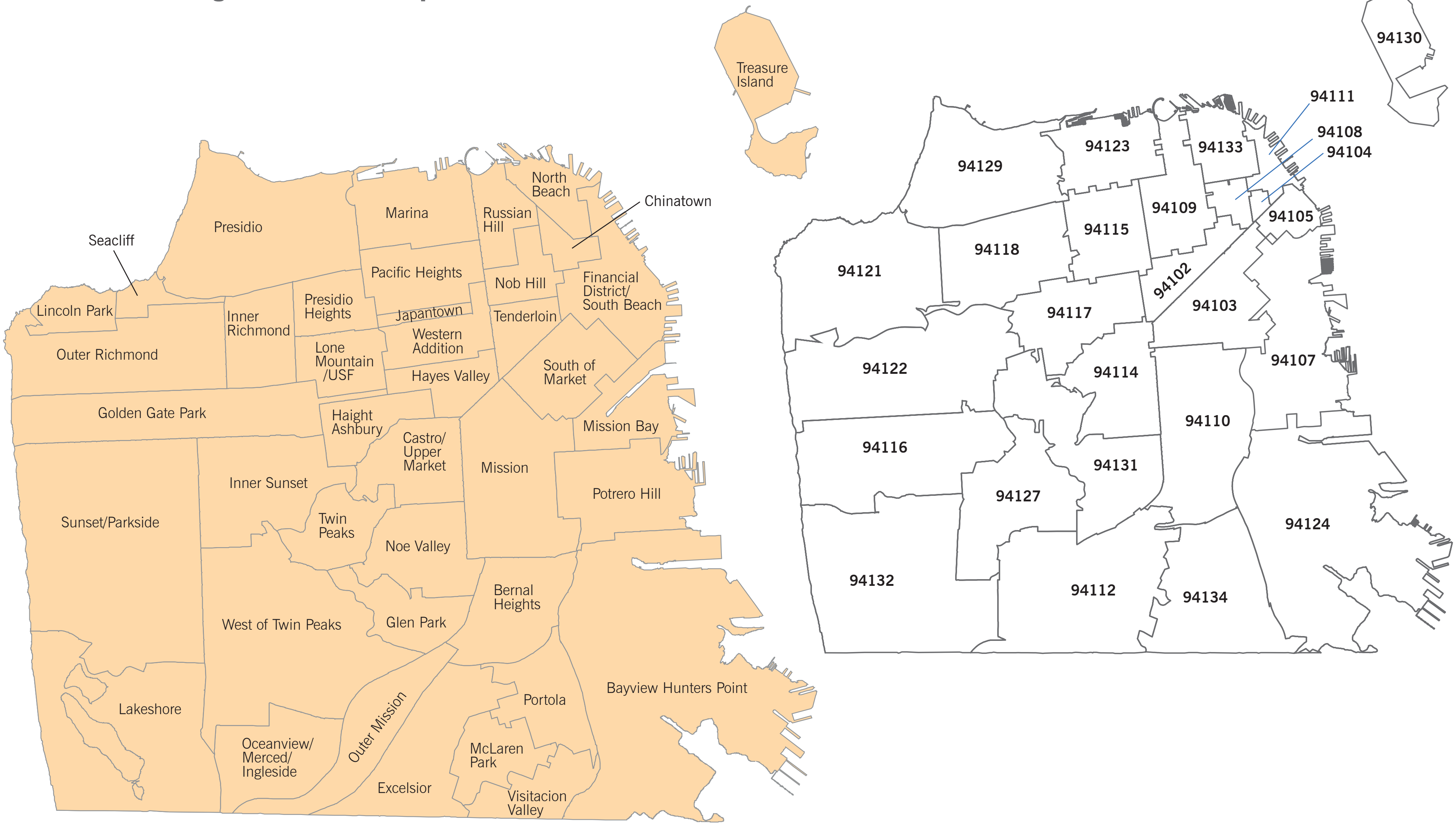

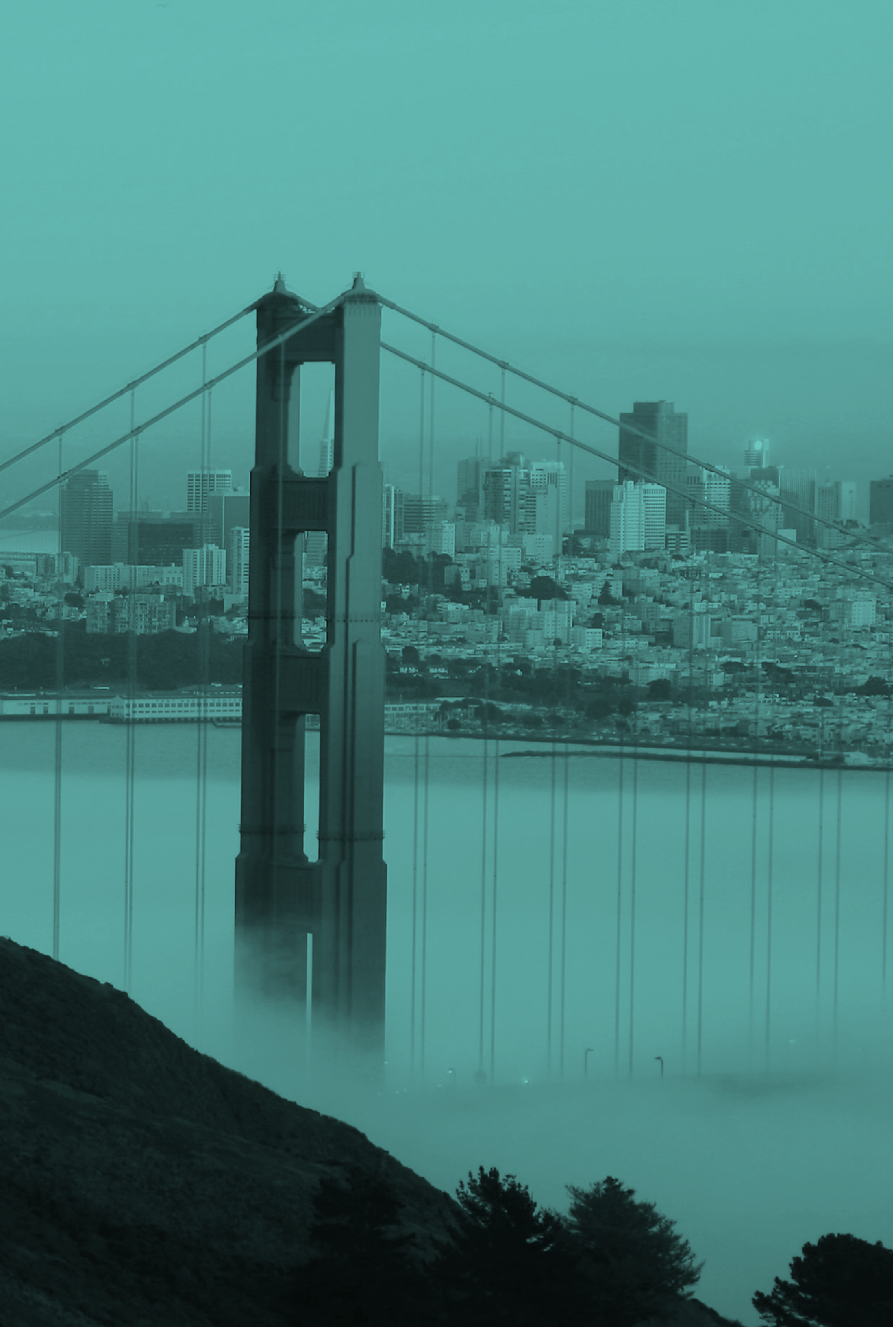

Major Findings

The 2019 Community Health Needs Assessment identified two foundational issues and five health needs.

The following infographics highlight aspects of each issue and need.

Foundational Issues

Poverty 16

Racial Health Inequities 17

Health Needs

Access to Coordinated, Culturally, and Linguistically Appropriate Care and Services.................................. 20

Food Security, Healthy Eating, and Active Living 21

Housing Security and an End to Homelessness 24

Safety from Violence and Trauma 26

Social, Emotional, and Behavioral Health 30

Poverty

Income generally confers access to resources that promote health—like good schools, health care, healthy food, safe neighborhoods, and time for self care—and the ability to avoid health hazards—like air pollution and poor quality housing.

Low income groups are at greater risk of a wide range of health conditions than higher income groups, and have a shorter life expectancy.[1]

People who live in communities with higher income disparity are more likely to die before the age of 75 than people in more equal communities.[2]

More than half of new jobs in San Francisco are expected to be low wage (<$54,000/year), service sector jobs.[3-4]

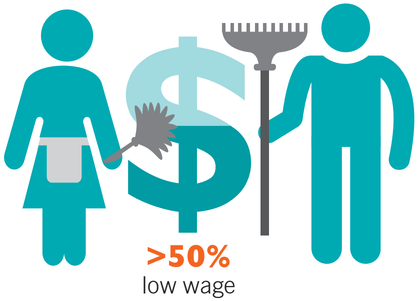

>50%
low wage

Household Income

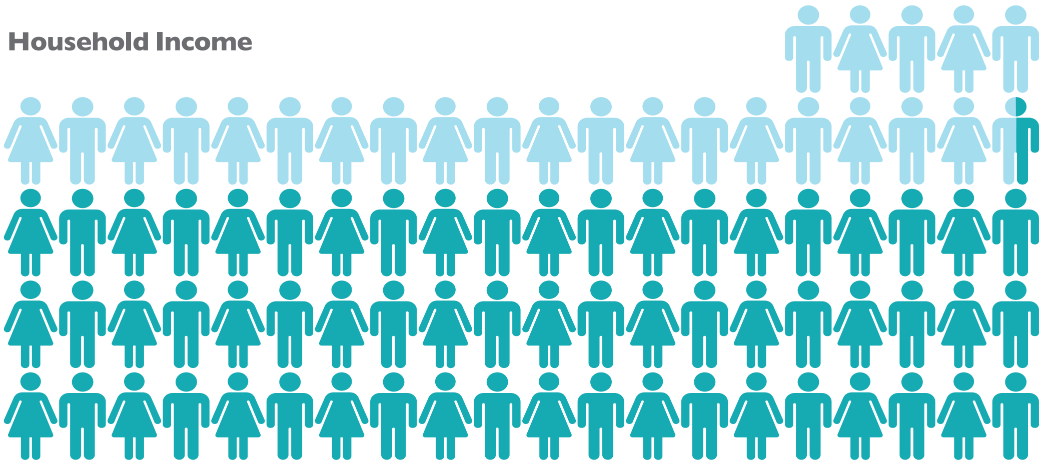

Almost 1 in 4 (22%) San Franciscans live below 200% of the federal poverty level.[3]

For a family of four, 200% of the Federal Poverty Level is $50,200.[4]

A family of four in San Francisco, requires an income of greater than $120,000 to meet all of their needs.[5]

40% of new jobs in San Francisco are expected to be low wage (<$54,000/ year) jobs.[6,7]

18% of children under 6 years of age in San Francisco live in poverty (<200% FPL).[8]

Employment Disparities

San Francisco has significant disparities in employment rates between Whites and Black/African Americans.[3]

96% of White San Franciscans are employed.

Only 83% of Black/African Americans are employed; Black/African American males have the lowest employment rate in San Francisco (81%).

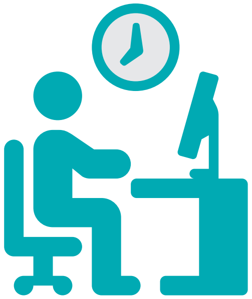

Black/African Americans are a third as likely as Whites to have a Bachelor's degree or higher and 5 times more likely to have less than a high school education.[3]

Median Income

In San Francisco, there is significant inequality in household income between races.[3]

White household median income is over **$111k**

Black/African American household median income is **$28k**

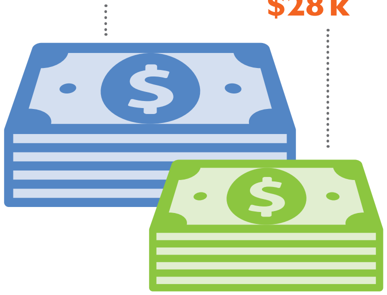

Income Inequality and Health

San Francisco has the highest income inequality in California.

The wealthiest 5% of households in SF earn 16 times more than the poorest 20% of households.[9]

Low income impacts lifetime health, beginning with pregnancy and birth.

Lower-income children in San Francisco experience higher rates of asthma, hospitalization, obesity, and dental caries.[10-12]

Low-birth weight is highest among low-income mothers.[13]

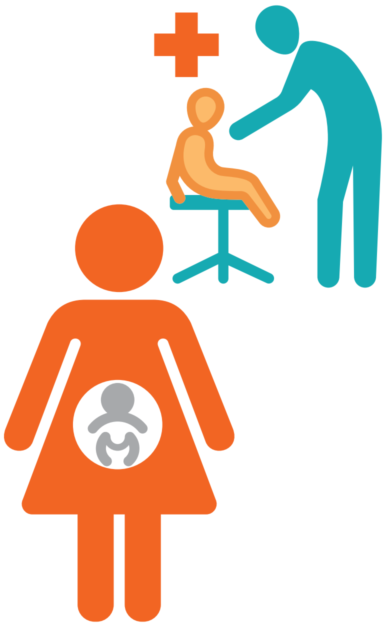

Major Findings
Foundational Issues

Racial Health Inequities

Two types of racialized social interaction, interpersonal and structural racism, play a role the racial health disparities seen in San Francisco.

Racial discrimination in interpersonal behavior, often called everyday racism or bias, sets the kind of experiences that make up the social lives of people of color. The accumulation of those experiences has been associated with increased hypertension, preterm birth and other conditions mediated by stress.

Long-standing social and institutional rules, both historic and current, determine which spaces and resources are available to marginalized groups. The disparate treatment of children based on race in schools and courts is an example of these forces. So are the historic differences in family wealth that stem from government housing policy and private banking rules. These forces are often intertwined and reinforcing as they occur over the life-course.

Racial inequities are not just a matter of unfortunate history, but of on-going, correctable injustice.

Improvements

For Black/African Americans improvements are seen in some social determinants and some health conditions. However, the improvements do not always impact the inequity as other groups may experience greater gains.

Indicator	Who Better for...
Teen Birth	Between 2007 and 2016 the teen birth rate for first time moms decreased from 34% to 10% among Black/African American women in San Francisco.[2] In that same time, the proportion of mothers who had a college education when they delivered their first baby increased by 16 percentage points.[2]
Mortality	Mortality rates decreased for all in San Francisco. However, rates decreased the most for Black/African Americans (15%) (vs. 11% for Pacific Islanders, 12% for Whites, 14% for Asians and Latinx). Decreased rates among Black/African Americans were primarily due to decreases in ischemic heart disease, lung cancer, assault, and HIV.[17]<br>Life expectancy also grew for all San Francisco with the largest gains seen by Black/African Americans. (+3 years between 2005–2007 and 2015–2017 vs +2 years for others).
High School Graduation	Graduation rates increased for all between 2012 and 2017. The biggest gains were seen among Black/African Americans (8%), and Pacific Islanders (12%) while rates for Latinx (4%), Whites (3%) and Asians (4%) were more modest.[3]
Childhood Caries	Between 2007–2012 and 2012–2017, rates of untreated tooth decay among kindergarteners decreased the most for Black/African Americans (26% to 19%).[8]

Population Loss

Between 1990 and 2005, the Black/African American population **decreased by 41%** from almost 79,000 to less than 47,000.

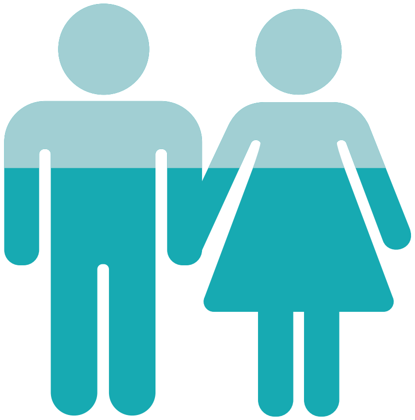

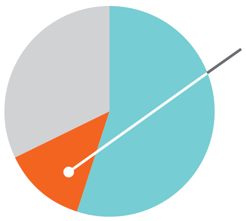

Between 1990 and 2005, the proportion of very low income households increased from 55% to **68%**.[18]

The strong association between poverty and health would suggest that the poorer remaining Black/African American population is more likely to have poor health than the previous more mixed-income population.

Racial Health Inequities

Basic Requirements for a healthy life span	Prebirth/Infancy	Childhood	Adolescence
	Healthy diet<br>Prenatal care	Adequate income, Engaged with school,<br>Social network, Adequate housing,<br>Healthy diet, Safety	Mistakes corrected<br>Schools well-resourced<br>School success

Children 0–18 Living in Poverty[3]

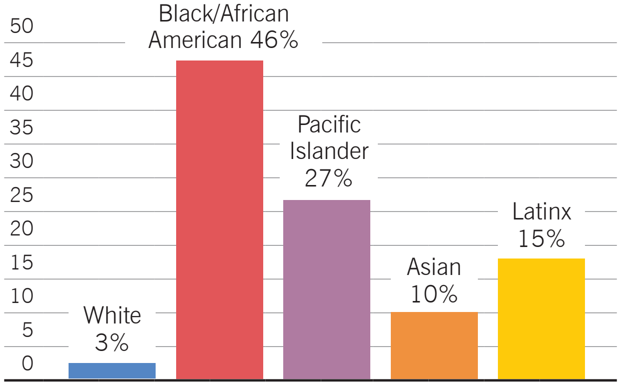

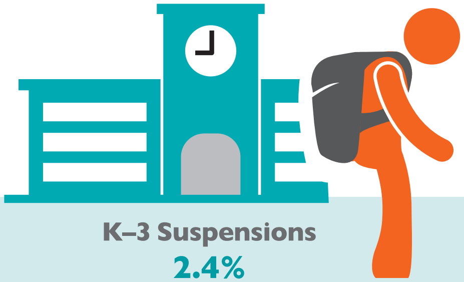

K–3 Suspensions
2.4%
suspension rate for Black/African Americans
vs 0.1%
for White SFUSD Students [4]

Student Proficiency

Black/African American Students
13% are proficient or above in mathematics, 19% in English language arts.[5]

Latinx students
22% of are proficient in mathematics, 28% in English language arts.

Pacific Islander Students
19% of are proficient in mathematics, 25% in English language arts.

White Students
70% are proficient in mathematics, 77% in English language arts.

Hurdles to a healthy life start early in San Francisco

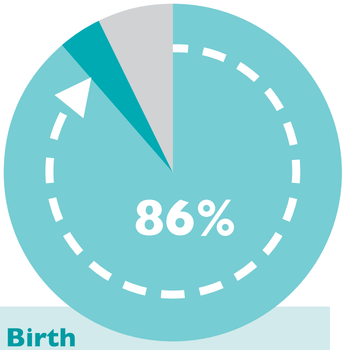

Full-Term Birth
Full term birth more likely for Whites (**93%**) than Black/African Americans (**86%**).[2]

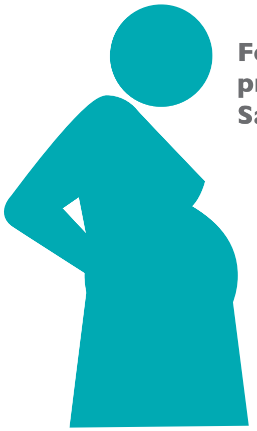

Food insecurity among pregnant women in San Francisco[1]

26.5% among Latinx women
19.5% among Black/African American women
6.6% among Asian and Pacific Islander women

Almost no White women in San Francisco report food insecurity during pregnancy.

Nutrition

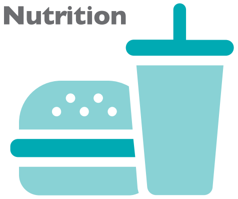

Black/African American and Latinx SFUSD students are 2–3 times more likely to consume fast food (**64%**, **73%**), or soda (**44%**, **36%**) at least weekly, as compared to White students (fast food (**35%**) and soda (**17%**). [6]

5th Grade Obesity[4]

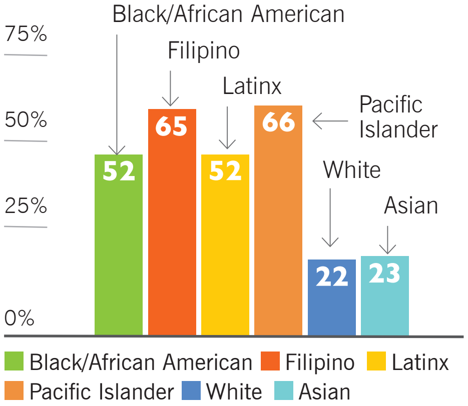

Racial Health Inequities

Basic Requirements for a healthy life span	Adolescence	Adulthood	Old Age
	Mistakes corrected<br>Schools well-resourced<br>School success	Employment, Stable housing<br>Active, Healthy childbearing<br>Freedom	Active lifestyle<br>Independence<br>Long life

Juvenile Detentions

Black/African American youth make up over **57%** of bookings at juvenile hall even though they make up only 6% of the population.[9]

Together Black/African American and Latinx youth comprise **86%** of all juvenile bookings. Samoan youth are also over-represented and make up **3%** of the bookings, but only account for less than 1% of the youth population.

Unduplicated Account of Juvenile Hall Bookings —Criminal Offenses by Ethnicity, 2017

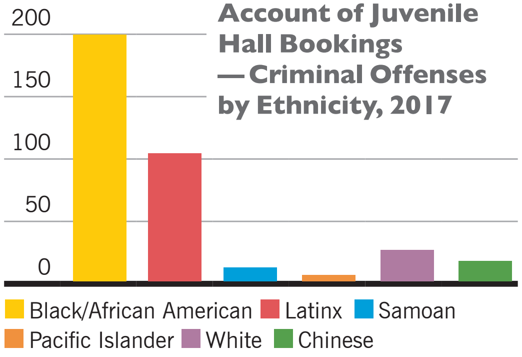

Educational Attainment 2012–2016[3]

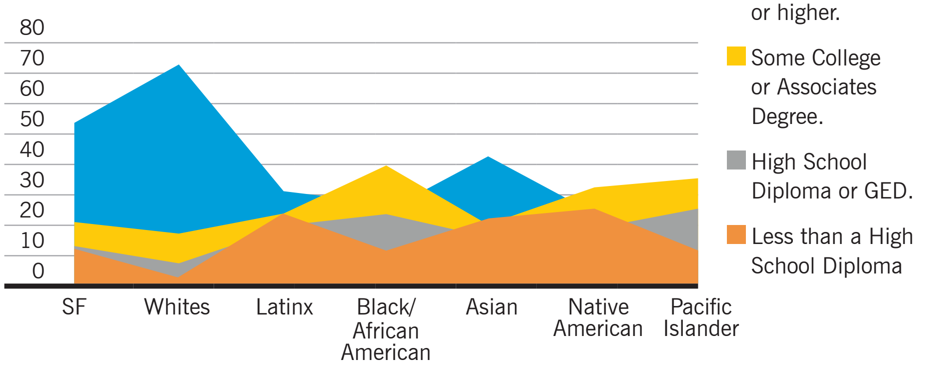

The starkest inequities are seen between Black/African American residents and all other groups, and occur across the lifespan.

Median Household Income

The median income in San Francisco varies greatly by race/ethnicity. Typically, Whites earn 4x more than Black/African Americans in San Francisco.[3]

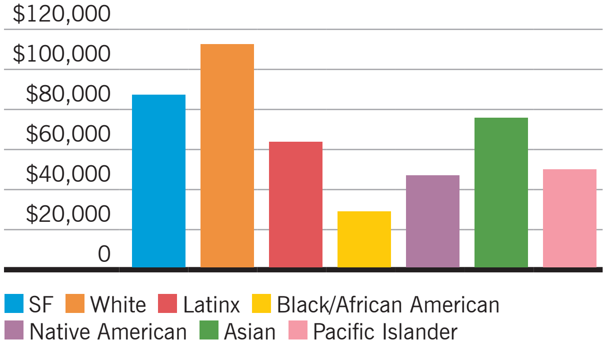

Homelessness

Black/African Americans are over-represented among the homeless in San Francisco.

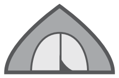

35%
of the homeless persons are Black/ African American

22%
are Latinx compared to

5% and **15%**,
respectively,
of the city overall.[3,10]

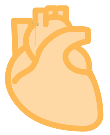

Heart Disease

Heart Disease impacts Black/African Americans at younger ages. Rates of heart disease related hospitalizations among Black/African Americans in their 40s and 50s **are comparable to those seen in other races/ ethnicities over 75 years of age.**[7]

	2005-2007			2015-2017		
	All			All		
All	80.8	84.0	77.6	83.1	86.1	80.3
Asian	85.1	87.5	82.4	87.0	89.6	83.9
B/AA	68.5	73.7	64.2	72.1	76.5	68.3
Latinx	82.7	85.8	79.4	85.1	87.9	82.5
PI	73.4	77.0		76.0	76.8	75.5
White	79.7	83.1	76.9	81.7	84.2	79.6

Major Findings
Health Needs

Access to Coordinated, Culturally and Linguistically Appropriate Care and Services

sfhip

Healthy People 2020 defines access to health care as "the timely use of personal health services to achieve the best possible health outcomes."[1]

Access is influenced by availability of providers, location, affordability, hours, and cultural and linguistic appropriateness of health care services. Accessible health care can prevent disease and disability, detect and treat illnesses, maintain quality of life, and extend life expectancy.[2]

From a population health perspective, regular access to quality health care and primary care services also reduces the number of unnecessary emergency room visits and hospitalizations and can save public and private dollars.

While access to health care in San Francisco is better than many other places, significant disparities exist by race, age, and income.

Many San Franciscans do not access health care

San Francisco's population now numbers **over 880,000 people**.

Fewer Uninsured

Over 10,000 fewer San Franciscans were uninsured in 2017 compared to 2015. However, 2% of San Franciscans, 16,000, still lack insurance or health care access via Healthy San Francisco or Healthy Kids.[3-4]

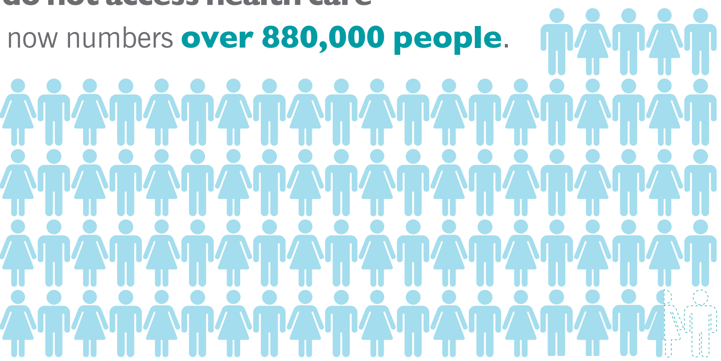

8% do not have a usual place to go for medical care.[5]

24% of adults have not had a routine check-up in the past year.[5]

51% have not had a flu shot in the past year.[5]

54% of women ages 18–44 have not received counseling or information about birth control from a doctor or medical provider in the past year.[5]

15% of women with public safety net insurance do not receive timely prenatal care.[6]

27% of adults have not seen a dentist in the past year.[5]

82% of Denti-Cal eligible infants aged 2 years or less do not access dental care.[7]

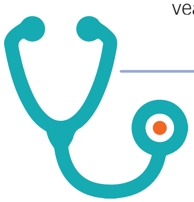

Young adults are at risk. Young adults 18 to 34 years of age and people of color are less likely to be covered by insurance.[4]

Language barriers and cultural competency of services are serious barriers to receiving quality care.

Increased cultural competence requires structural and systemic improvements, and can be linked to improvements in healthcare access, participation, and patient satisfaction.[10-11]

From the community we heard...

"Cultural competency doesn't happen with just a class or a one-day training."

"Healthcare professionals need to be from the community and actually know the culture of the community."

"Community-based organizations serve a critical role in small, datasparce cohorts, by informing public health efforts and bringing resources to multicultural communities."

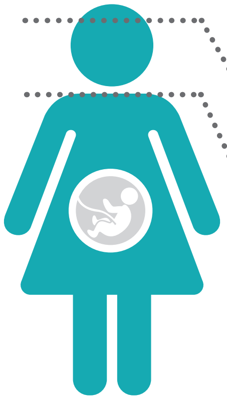

Different Levels of Prenatal Care

In 2013-15, **≥99%** of mothers with private insurance received prenatal care in the first trimester.[6]

Only **86%** of those with Medi-Cal received early prenatal care.[6]

Residents covered by public safety net insurance do not receive preventative care at the same rate as those with private insurance.

Preventable Hospitalizations and Emergency Room Visits

While preventable hospitalizations for most causes have decreased over time, preventable hospitalizations for hypertension and diabetes have respectively increased **45%** and **50%** between 2011 and 2016— potentially indicating these conditions are not being well managed at the population level.[8]

Preventable hospitalizations and ER visits are significantly higher among Black/African Americans and Pacific Islanders compared to all other ethnicities in San Francisco.[9]

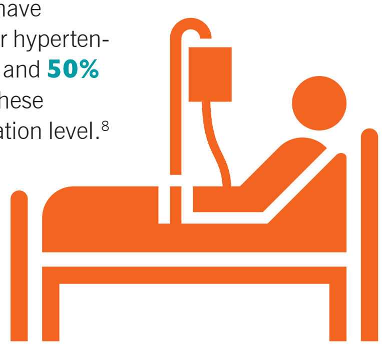

Food Insecurity, Healthy Eating, and Active Living

sfhip

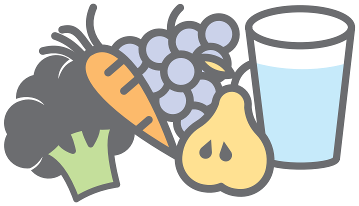

Good nutrition means getting the right amount of nutrients from healthy foods and drinks. Good nutrition is essential from infancy to old age.

The USDA's MyPlate.org recommends that fruits and vegetables make up at least half of our plate, or approximately five servings a day.[1]

Leading medical and health associations recommend drinking water instead of sugary drinks.[2] The institute of Medicine recommends 13 cups of liquids per for men and 9 cups for women who live in temperate climates.[3]

A healthy diet promotes health and reduces chronic disease risk. It is critical for growth, development, physical and cognitive function, reproduction, mental health, immunity, stamina, and long-term good health.[4]

Many in San Francisco are food insecure

50% of low income residents surveyed in SF report food insecurity.[6]

20–30% of Black/African American and Latinx pregnant women are food insecure.[5]

50% of SFUSD students qualify for free or reduced-price meals.[9]

Over **100,000** food insecure adults and seniors are eligible to receive meals, groceries or eating vouchers.

Services to ameliorate food insecurity are not meeting need

70% Percentage of eligible students not participating in the Summer Lunch Program.

-7% Decrease in the number of food vendors authorized to acccept food stamps.[14]

1,969 The number of meals denied Seniors and persons with disabilities at congregate meal sites.[6]

21 days/187 days The number of days seniors/persons with disabilities must wait to start getting home delivered meals.[6]

616 The number of persons waiting for enrollment at a food pantry.[33]

The USDA has designated the Oceanview, Merced, Ingleside, Bayview Hunters Point, Visitation Valley and Treasure Island neighborhoods as areas of low food access.[10]

Many in San Francisco do not eat and drink healthily

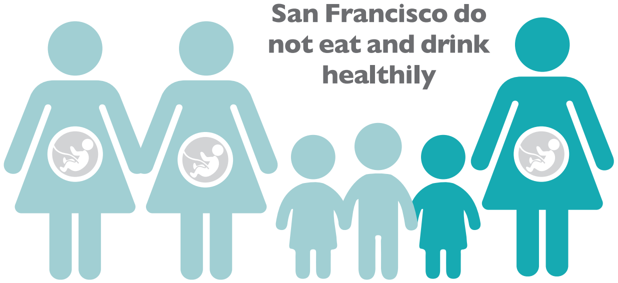

2 out of 3 pregnant women in the WIC Eat SF program and **2 out of 3 youth** do not eat 5 or more servings of fruits or vegetables daily.[5]

Some San Franciscans do not drink enough water

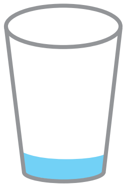

614 people were hospitalized for "potentially preventable" dehydration in 2016.[7]

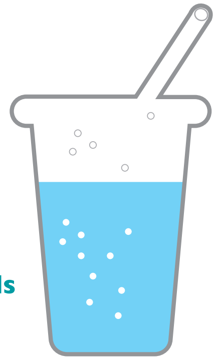

Many do drink sugary drinks. **Two thirds** of high school students and **one third** of young adults regularly consume soda.[8]

Facilities necessary to eat and drink healthily are not available for all

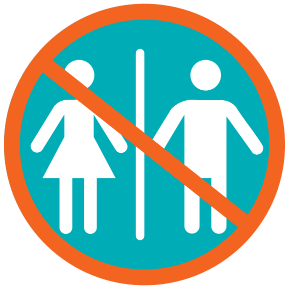

Barriers to drinking enough water include limited access to bathroom facilities to go to the bathroom.[31-32] **San Francisco operates 28 public restrooms that are open all day, which amounts to 3.3 restrooms per 100,000 residents.**[13]

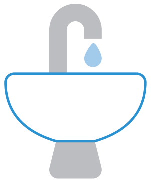

The Mission, Bayview Hunters Point and Treasure Island districts **each have only one public access drinking water fountain.**[12]

Not all have a kitchen to cook in. Over 21,000 occupied housing units in San Francisco do not have complete kitchen facilities.

Food Insecurity, Healthy Eating, and Active Living

Regular exercise extends lives.

The World Health Organization (WHO) recommends that children and adolescents, age 5 to 17 years, should do at least one hour of moderate-to-vigorous physical activity daily, while adults, age 18 years and above, should do at least 150 minutes of moderate-intensity physical activity, 75 minutes of vigorous-intensity physical activity, or an equivalent combination of moderate and vigorous activity throughout the week.[15]

Just 2.5 hours of moderate intensity aerobic physical activity each week is associated with a gain of approximately three years of life.[16]

Walking is a simple, affordable way for people to get around. A walkable city provides a free and easy way for people to incorporate physical activity into their daily lives as they walk to work, to school, to the market, to transit or other nearby services, or just for fun.[17]

Many San Franciscans don't spend the recommended amount of time doing physical activity

1 out of 2
(56%) adults does not walk at least 150 min per week for transportation or leisure.[18]

1 out of 2
(47%) children ages 3–5 years in child care centers are not physically active for 90 min per school day.[19]

2 out of 3
(67%) middle schoolers do not spend 60 min per day each day of the week doing physical activity.[20]

4 out of 5
(83%) high schoolers do not spend 60 min per day each day of the week doing physical activity.[20]

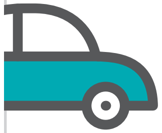

Each day,
4.5 million
transportation trips are made in San Francisco.

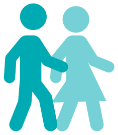

Of these, only about **37%** are walking trips or public transit trips which include walking.[21]

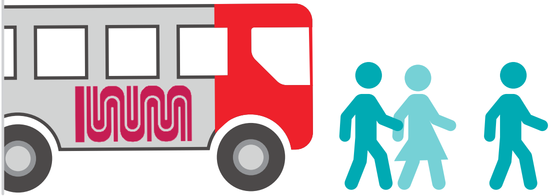

Many San Franciscans don't meet activity standards

In San Francisco about **30%** of 5th and 7th graders and **40%** of high school students do not meet the Fitnessgram standard for aerobic capacity, which is ability to run one mile or pass a PACER test.

60 percent of Black/African American and Latinx 9th graders, do not meet the fitness standards, compared to 30% of White and Asian students. [27]

Aerobic fitness is 10 percentage points lower for economically disadvantaged students [27]

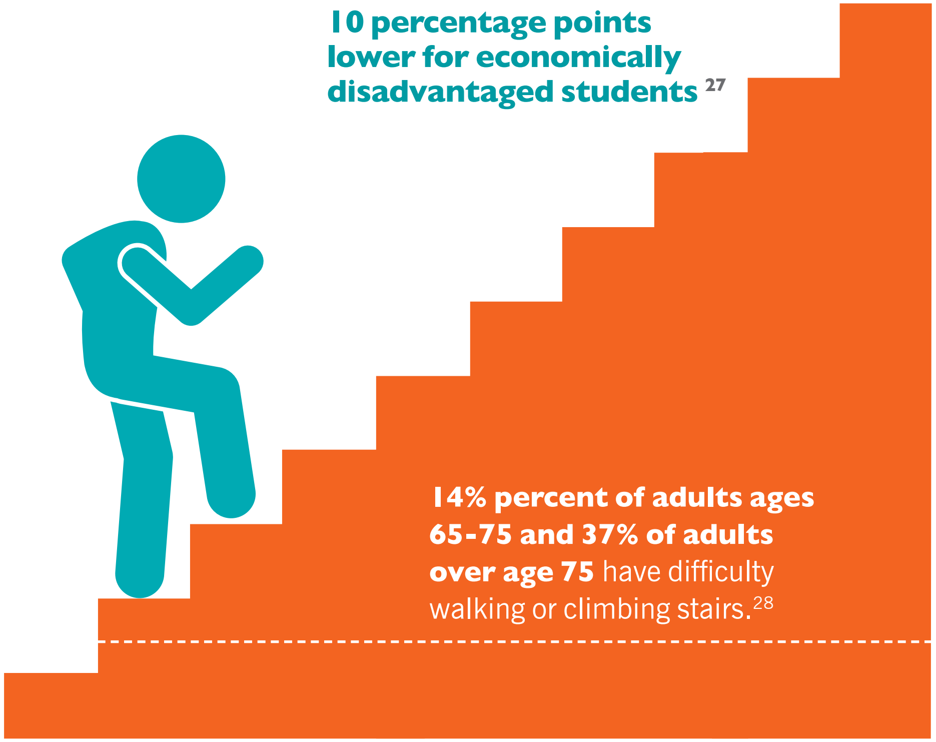

14% percent of adults ages 65-75 and 37% of adults over age 75 have difficulty walking or climbing stairs.[28]

Major Findings
Health Needs

Food Insecurity, Healthy Eating, and Active Living

Safety, and a lack of resources and other supports are barriers to physical activity in San Francisco

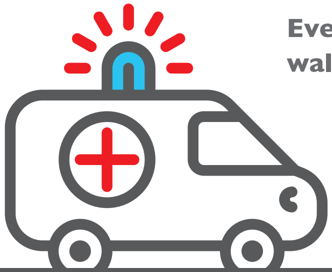

Every day, on average 2 people walking are hit by cars

Cars violating a pedestrian's right-of-way is the top risk factor for injuries to people walking. **In 2018, there were 15 pedestrian deaths and 3 cyclist deaths.**[22-23]

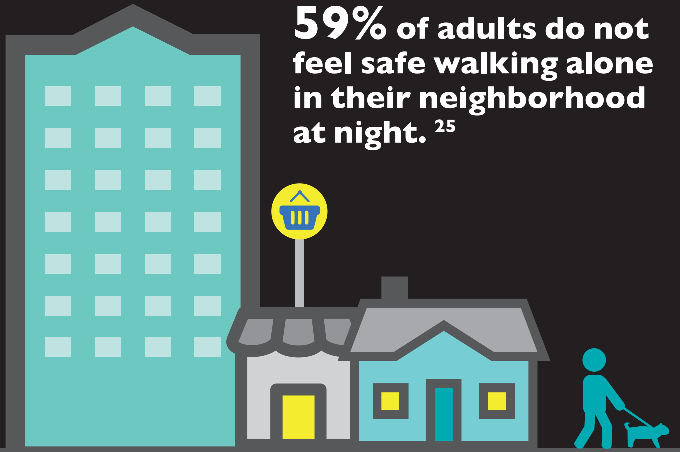

59% of adults do not feel safe walking alone in their neighborhood at night.[25]

Vision Zero High Injury Network 2017 Update San Francisco California[21]

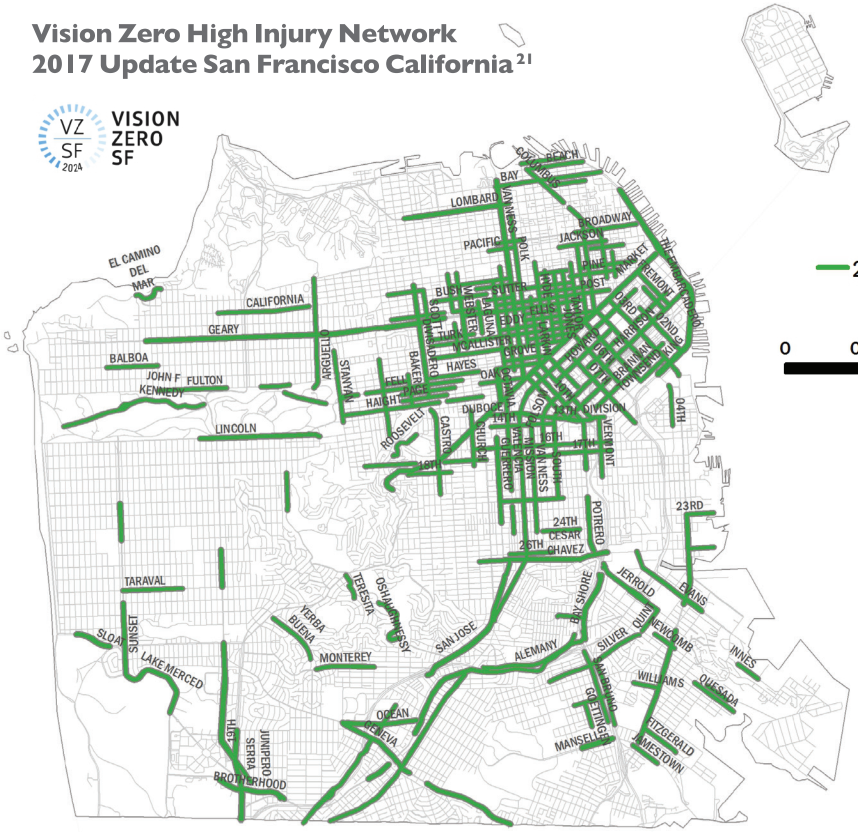

There are gaps in neighborhood resources for physical activity

Sidewalk networks support walkers to varying degrees. Downtown and in Chinatown, the blocks are short and provide many pedestrian connections. In other neighborhoods, pedestrians have to walk further to make less direct connections.[34]

35% of San Francisco playgrounds do not score an A or B for infrastructure quality, cleanliness and upkeep.[26]

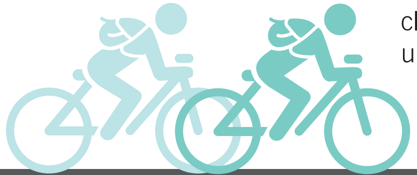

There are gaps in school and workplace supports for physical activity

2 out of 3 (67%) child care centers do not use physical activity curriculum.[29]

> *All of our students, regardless of which neighborhood they live in or which school they attend, should be able to safely walk or bike to school. We are adding crossing guards across the City and I am pushing the SFMTA to expedite Vision Zero projects because we do not have time to waste. We need safer, more livable streets now."*
>
> — MAYOR LONDON BREED[23]

Although each April, more than 10,000 people participate in Walk to Work Day, including San Francisco's Mayor and Supervisors, **over 200,000 workers drive to work on a daily basis.**[30]

SF has 0.18 miles of bike lane for every 1 mile of streets.[24]

Housing Security and an End to Homelessness

sfhip

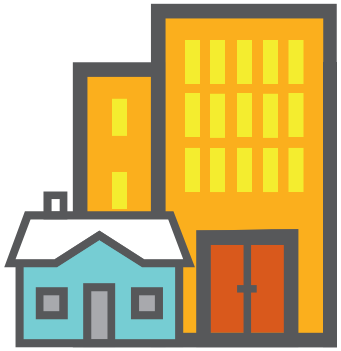

Shelter is a basic human need

Housing is foundational to meeting people's most basic needs. Quality housing provides a place to prepare and store food, access to water and sanitation facilities, protection from the elements, and a safe place to rest. Stable/ permanent housing can also provide individuals with a sense of security. Unfortunately, California, and especially the Bay Area, suffers from an acute housing shortage which has been driving housing costs to unaffordable levels, leading an increasing number of residents to become homeless.[1]

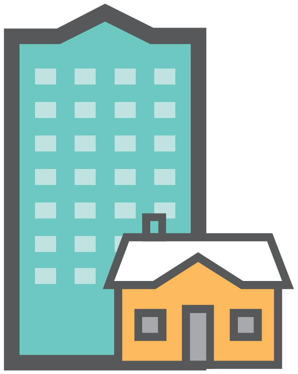

Housing production has declined in the Bay Area

Between 2011 and 2015, the Bay Area added 501,000 new jobs — but only 65,000 new homes.[2]

Housing Production Decline in the Bay Area, 1970–2015

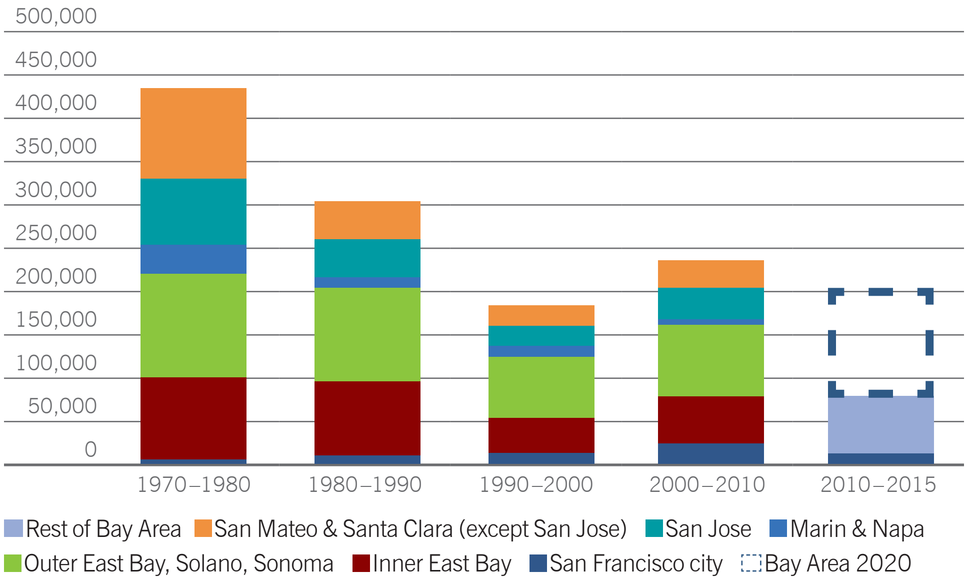

Source: *SF Planning Analysis of US Census and ACS Data*

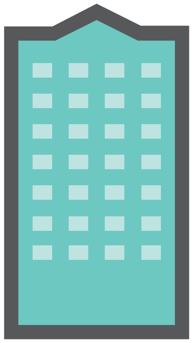

San Francisco usually exceeds requirements for development of above moderate-income housing (120% AMI), but builds less than a third of the units allocated for moderate and low-income residents.[3]

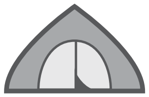

Homelessness

In 2017, about 7,500 homeless persons were counted in San Francisco.[7] Despite making up only 6 percent of the general population, **35% of the homeless persons counted were Black/African American**.

Among the many challenges homeless persons face, including those in temporary housing, are: [8-9]

- Safely storing medications
- Eating healthfully
- Finding a job
- Maintaining relationships
- Going to the doctor

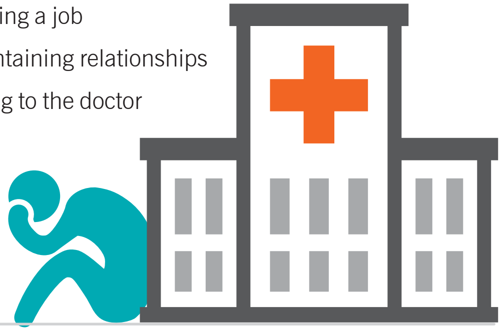

Overcrowding

An estimated 24,000 people in San Francisco live in crowded conditions.[4]

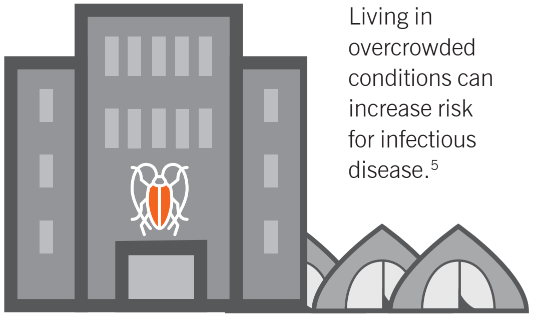

Living in overcrowded conditions can increase risk for infectious disease.[5]

Housing Security and an End to Homelessness

Housing Affordability

Between 2010 and 2018, the median market rate rent for a 2–bedroom unit **increased 48%** to $4,725.[10]

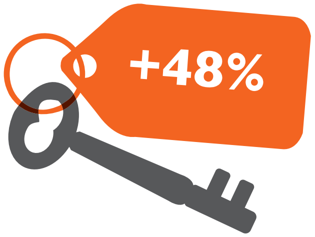

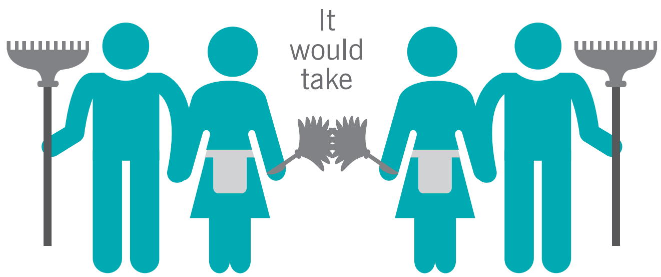

4 full-time minimum wage jobs to afford a "fair market rate" ($3,121) 2–bedroom unit [11]

6 full-time minimum wage jobs to afford a "median market rate" ($4,725) 2–bedroom unit [10]

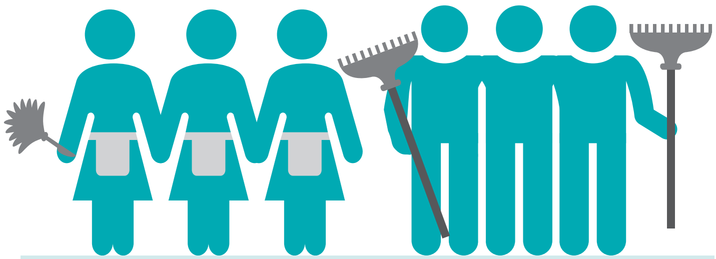

The median percent of income paid to gross rent in San Francisco was **30%** in 2017.
17% of renter households spend **50%** or more of their income on rent.[4]

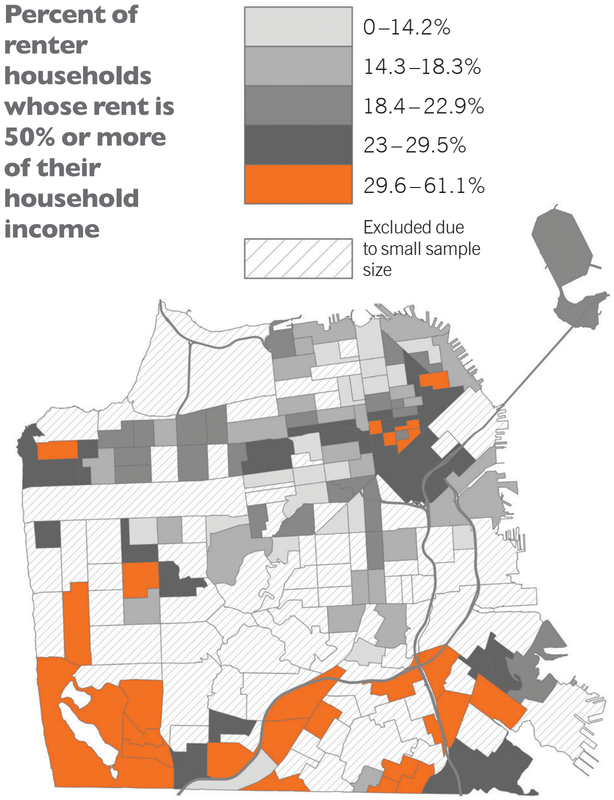

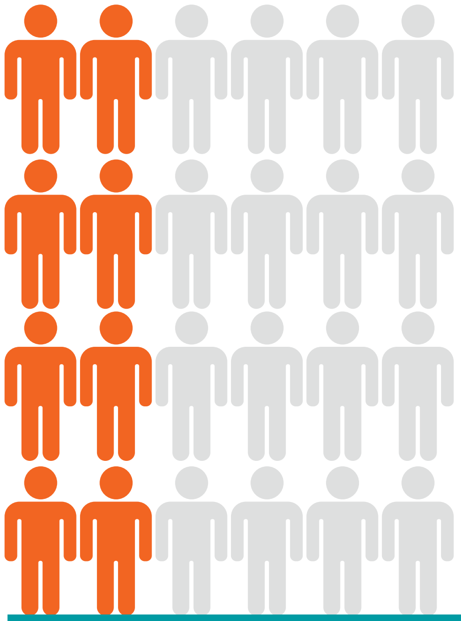

Nearly one-third of Chinatown residents live in overcrowded conditions.[12]

Evictions

There had been a steady increase in the number of all-cause eviction notices between 2011–2016; however, **in 2017 there was a 27% decrease in the number of eviction notices filed**.[6] This rapid change may be attributable to the implementation of Eviction Protection 2.0 in November 2015, as well as economic shifts and other factors.

Moving can result in: [5]

- Loss of employment
- Difficult school transitions
- Increased transportation costs
- Loss of health protective social networks

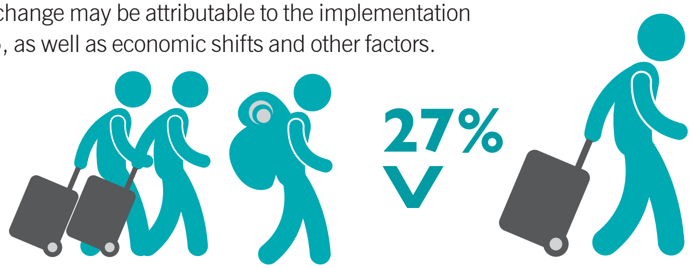

Major Findings
Health Needs

Safety from Violence and Trauma

Violence not only leads to serious mental, physical and emotional injuries and, potentially, death for the victim, but also negatively impacts the family and friends of the victim and their community.

Community violence decreases the real and perceived safety of a neighborhood disrupting social networks by inhibiting social interactions, causing chronic stress among residents who are worried about their safety, and acting as a disincentive to engage in physical activity outdoors.[5-8]

Children are particularly vulnerable. Witnessing and experiencing violence disrupts early brain development and causes longer term behavioral, physical, and emotional problems.[1-4]

Violence is rarely caused by a single risk factor but instead by the presence of multiple risk factors. Some risk factors for violence are: poverty, poor housing, illiteracy, alcohol and other drugs, mental illness, community deterioration, discrimination and oppression, and experiencing and witnessing violence.[9-11]

Violent Crime is a Concern in San Francisco.

Violent crime rates in San Francisco are high (712/100,000) and exceed California rates (452/100,000).[12]

Violent Crime Rates

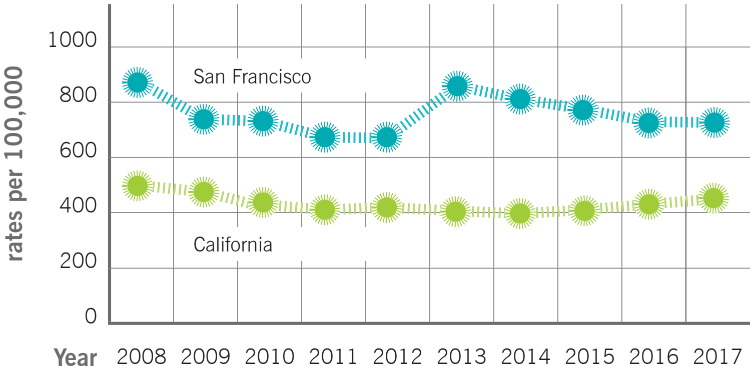

Crime	SF*	CA*
Homicide	6	5
Rape	41	37
Robbery	364	143
Aggravated assault	301	267

*Number of crimes per 100,000 residents.

Violent crimes rates, by analysis neighborhood, 2017[14]

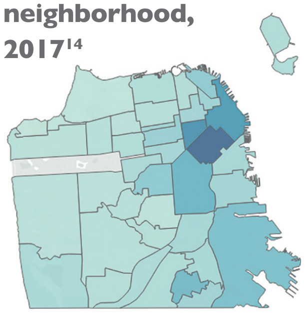

Crime rate (per 1,000)
0.00 — 91.82%

Young men, people of color, and residents of the Eastern neighborhoods are most likely to be victims of violence or to witness violence.

Violent Crime Rate

Violent crime rates and rates of emergency room visits due to assault are highest in the Eastern half of the City. Residents are also less likely to feel safe in these neighborhoods.[13-15]

122 males died violent deaths between 2015 and 2017.

Violence is the **5th** leading cause of death among Black/African American men and the **8th** cause among Latinx men.

Violence kills men in their prime years. **37** was the average age at death for men who died violently.[16]

89 of the **134 assault deaths** (66%)resulted from use of a firearm.

Emergency room visit rates[13]

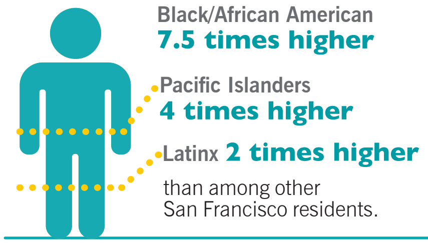

Emergency Room Visit Rates for Assault, 2012–16[13]

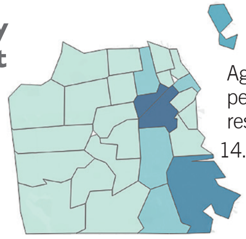

Age-adjusted rate per 10,000 residents.
14.1 — 203.7

Perceived Safety in San Francisco

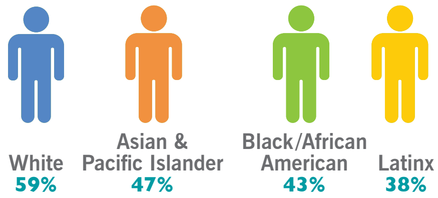

Residents perceived safety during the day, 2017[15]

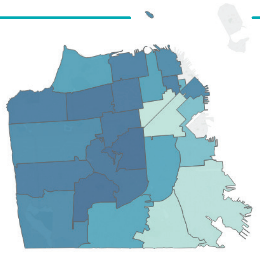

56.74% — 97.92%

Safety from Violence and Trauma

sfhip

Cases of child abuse have decreased in San Francisco since 2009.

However, in 2017 there were 509 cases of substantiated child maltreatment in San Francisco. The majority of child abuse cases are due to neglect.[17]

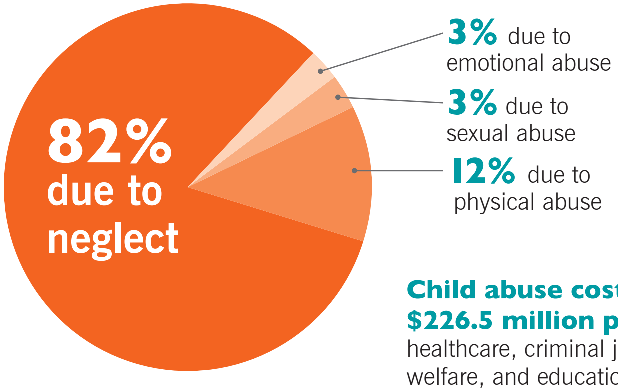

Child abuse costs the city $226.5 million per year in healthcare, criminal justice, child welfare, and education costs and lost lifetime productivity.[18]

Substantiated cases of child maltreatment per 1,000 children in San Francisco 2007–2017

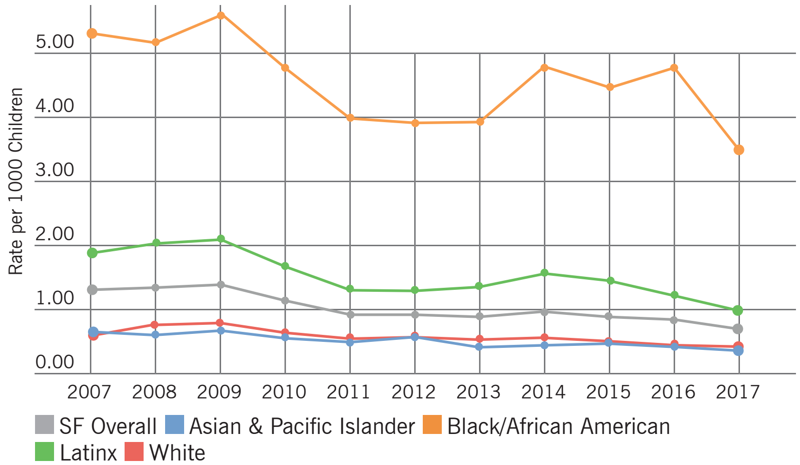

The rate of substantiated maltreatment among Black/African Americans is significantly higher suggesting a need for greater support.

The FBI has identified SF as one of the worst areas in the country for the commercial exploitation of children.

673 survivors of human trafficking were identified in SF in 2017.[39]

33% of persons trafficked in commercial sex were minors

71% of those who are trafficked are women, cigender or transgender people.

33% of victims were born in the Bay Area.

70% of survivors were people of color with the largest groups being Black/African Americans and Latinx.

In addition to a history of violence in family and community, maltreatment arises from the confluence of other preventable risk factors including:[18]

- **High Unemployment and Poverty**
  19% of Black/African American children in San Francisco live in poverty (<100% FPL); 7% of Latinx, 4% of Asian and 1% of White.[19]
- **Social and Social Economic Status Inequality**
  San Francisco has the 6th highest income disparities in the US.[20]
- **Low Levels of Education**
  Only 24–26% of Black/African American, Pacific Islander and Asian residents have a bachelors degree or higher. 32% of Latinx, 43% of Asian and 74% of White residents.[19]
- **Parenting Stress**
  28% of Latinx births in San Francisco are unintended, 24% of Black/African American, 20% of Asian, and 12% of White.[22] 27% of Latinx new mothers in San Francisco experience prenatal depression, 21% of Black/African American, 12% of Asian,and 10% of White.[22]
- **High Residential Instability**
  According to 2016 data, 2,512 or 4% of SFUSD students are homeless.[21] Less than 25% of Black/African American, Latinx, and Native American residents own their homes.[23]
- **Social Isolation and Lack of Social Support**
  In San Francisco 18% of Households have minors compared to 36% in California.[19]
- **Substance Abuse or Mental Health Issues**
  27–30% of Latinx, Black/African American and White residents report needing help with mental health or Drug Use Problems. 11 % of Asian reported needing help.[24]

Safety from Violence and Trauma

In San Francisco, steps have been taken to combat the school-to-prison pipeline.[35-37] However, Black/African American, and Latinx students are still more likely to be suspended or expelled and, with Samoan youth, are more likely to be arrested.

During the 2016–17 school year nearly 40% of all SFUSD students who received at least one suspension were Black/African American, despite making up only 11% of the student population.

Suspension rates for Black/African American and Pacific Islander students are 5x higher than those of Asian students.

Measure of School Discipline: SFUSD K–12 Suspension Rate, 2012–17

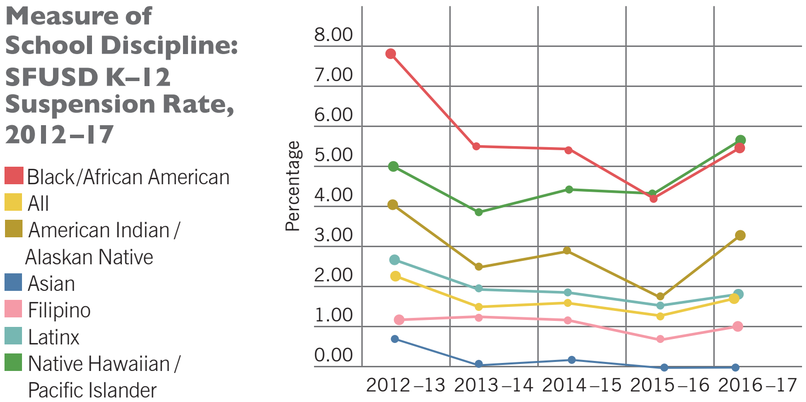

Unduplicated Count of Juvenile Hall Bookings/Criminal Offenses, by Zip Code, 2017

Count of Bookings
0 – 77

Source: San Francisco Juvenile Probation Department, 2017 Statistical Report.

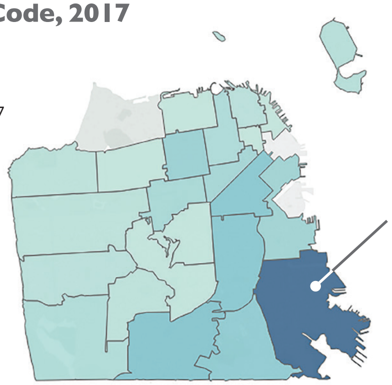

Zip code 94124, which roughly covers the Bayview neighborhood, was home to nearly 22% of all of the youth booked at Juvenile Hall in 2017.

Contributors to the school-to-prison pipeline include:

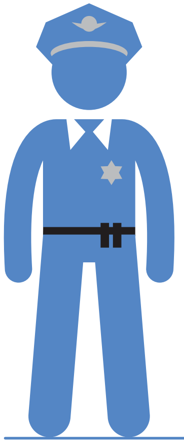

Inadequate resources (e.g. overcrowded classes, lack of counselors, special education services)

Police presence at schools

Harsh punishments that result in suspensions and out of class time.[33]

An arrest, a court appearance, and even brief detention, especially for minor infractions, increase a minor's risk of dropping out and getting into more serious crime.[34]

Once a student enters the juvenile justice system they face barriers to re-entry into traditional schools and many never graduate from school.[33]

Unduplicated Count of Juvenile Hall Bookings/Criminal Offenses, by Ethnicity, 2017

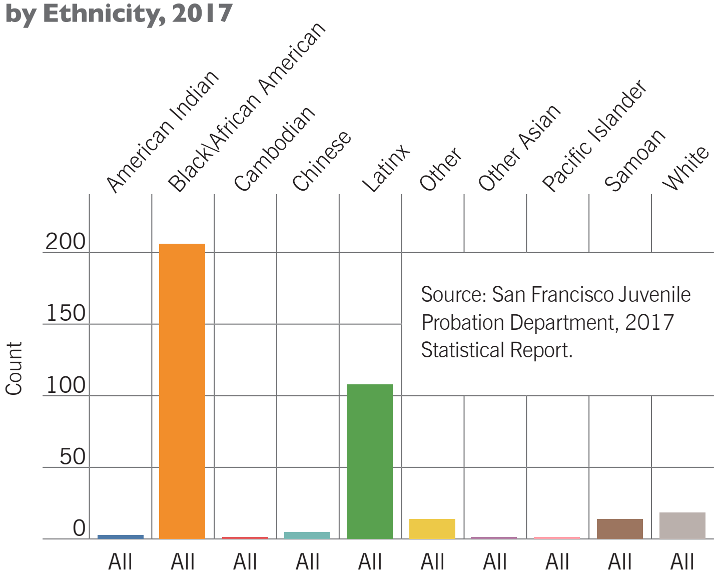

86% of Juvenile Hall Bookings are among Black/African American and Latinx youth.[38]

Samoan youth make up 3% of the bookings, but only account for less than 1% of the youth population.

Black/African American and Latinx persons are disproportionately detained, searched and arrested by the police in San Francisco.[25-28]

Incarceration harms the mental and physical health of the incarcerated and that of non-incarcerated partners and children. Mass incarceration also compromises the community health and contributes to racial health inequities.[29] At the population level, inequalities in incarceration impact employment and health which themselves further influence incarceration.[30]

Black/African American defendants experience delays in the criminal adjudication process, are convicted of more serious crimes and receive longer sentences than White defendants.[32]

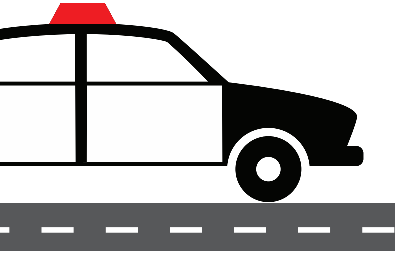

Officer Initiated Detentions, 2017

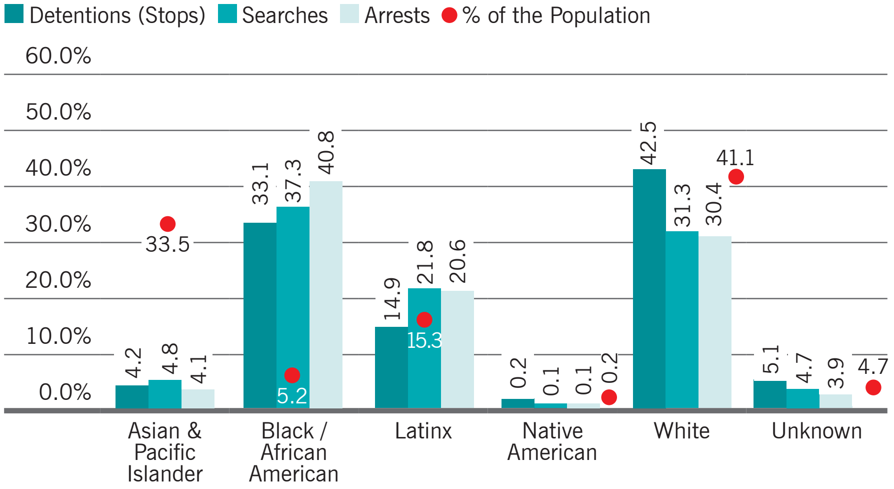

Officer Initiated Traffic Stops, 2017

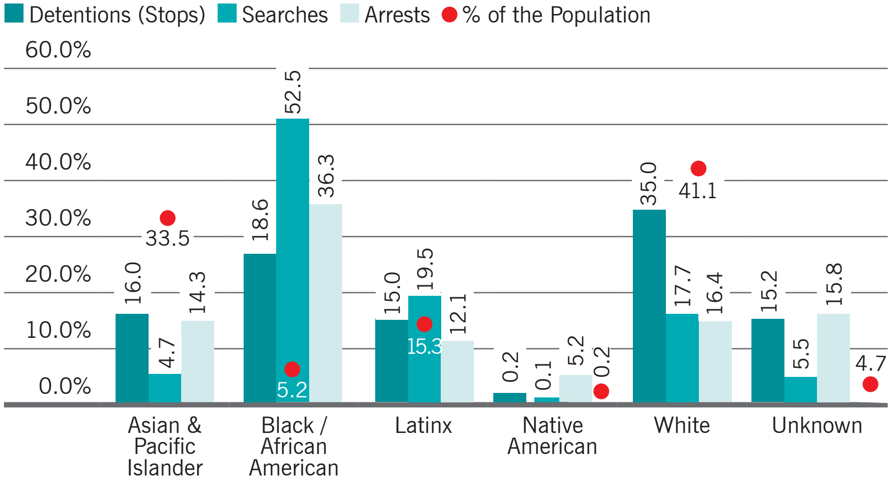

Detentions, searches, arrests and % of population each sum to 100%

Criminal History has a "ripple effect"

Differences in the severity of charges at booking and the number of times that people of color were previously arrested, convicted, and incarcerated explain almost all of the difference in conviction rates.

Pretrial Custody Black/African American defendants are held in pretrial custody **62% longer** than whites

Adjucation Process Time Cases involving Black/African American defendants take **90 days** for Black/African Americans, but only **77.5 days** for Whites.

Conviction Defendants of color are convicted of more serious crimes. Black/African American defendants are convicted of **60% more** felonies and **10% fewer** misdemeanors. Latinx defendants are convicted of similar number of felonies but 10% more misdemeanors.

Length of Sentence Black/African American defendants receive sentences which are **28% longer** than for whites. Latinx defendants received probations which were **55% longer.**

Non-consensual Searches Data from 2015 suggest that SFPD performs non-consensual searches among them with lower levels of evidence than for other racial and ethnic groups.[31]

While Black/African Americans make up 5% of the population in San Francisco, in 2017 they accounted for **33% of officer initiated (non-dispatched) detentions** and **19% of officer initiated traffic stops**.

Social, Emotional, and Behavioral Health

Mental health and well-being are crucial to supporting, maintaining, and optimizing quality of life.[4]

The presence of mental illness can adversely impact the ability to function at work, at home, and in social settings and impacts individuals as well as their respective families and communities.[1-3]

Mental disorders include:

- Depression
- Schizophrenia
- Anxiety
- Injuries to the brain
- Dementias
- Intellectual disabilities
- Developmental disorders (e.g. autism)
- Substance abuse.[1]

Social isolation can be a precipitating factor for suicidal behavior.

Individuals who experience isolation in their lives are more vulnerable to suicide than those who have strong social ties with others.[8]

- Impaired quality of life
- Disability
- Hospitalization
- Institutionalization
- Incarceration
- Suicide, self-injury, and/or death.[1]

People with lower education, income, and/or social status, and those who experience discrimination on the basis of race, gender, social class, or other characteristics are at a particularly high risk of mental illness.

23.3%
of adults reported needing help for mental health or substance use issues in 2011–2016.[6]

7%
of adults experienced serious psychological distress in 2014–2016.[6]

$
Lower income residents
are almost 3 times more likely to experience serious psychological distress than higher income residents (15.19% compared to 5.31%).[6]

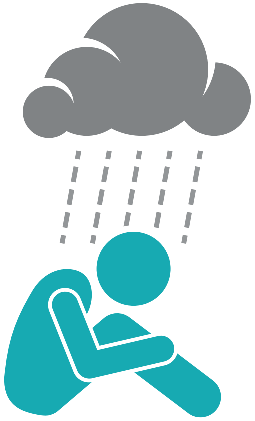

Depression is the most common mental illness.[3]

Depressive symptoms are common among San Francisco school-aged youth.5

High School depression 26% of SFUSD high school students reported prolonged sad or hopeless feelings in 2017.

Considering suicide Almost 13% of SFUSD high school students and 20% of middle school students had considered attempting suicide in 2017.

Sexual identification and depression Bisexual and gay or lesbian high school students are more likely to report prolonged sadness or hopelessness (45%-62%) and suicidal thoughts (32-40%) than heterosexual students (22% and 10%, respectively).

Between 2013 and 2015, 14.4% of pregnant women reported prenatal depressive symptoms in San Francisco.[4]

Prenatal depression greatly affects the quality of care given to the infant. **14.4% of pregnant women reported prenatal depressive symptoms** in 2013-2015.[11]

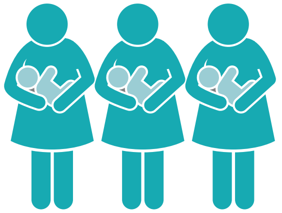

Women with less than high school education are more than 3 times more likely to report prenatal depressive symptoms than women with a college degree (37.6% vs 9.0%).

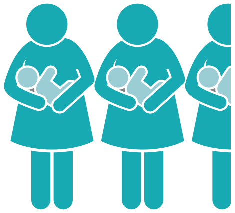

Women with Medi-Cal insurance are more than 2.5 times more likely than women with private insurance to report prenatal depressive symptoms (24.1% vs 8.9%).

Hispanic and Black/African American women are more likely to report prenatal depressive symptoms than White or Asian women.

Hospitalizations in San Francisco to treat major depression among adults occurred **2,631 times** during the three years between 2014 and 2016.[7]

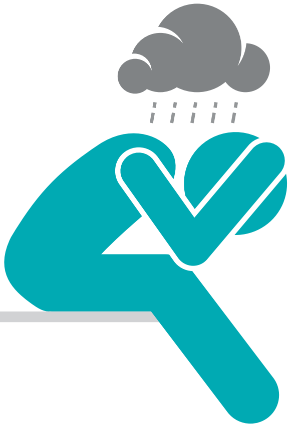

The number of hospitalizations for depressions exceeded that for hypertension (2296), asthma (1017).[7]

Adults aged 18-24 years are the most likely to be hospitalized due to major depression followed by 45-54 years. [7]

Age-adjusted rate of hospitalizations due to major depression among Black/African Americans is almost 5 times higher than among Asian & Pacific Islanders who have the lowest rate (23.79 vs 4.93 per 10,000 residents). [7]

Age-adjusted Mortality Rates due to Suicide by Race/Ethnicity in San Francisco, 2015–2017 [8]

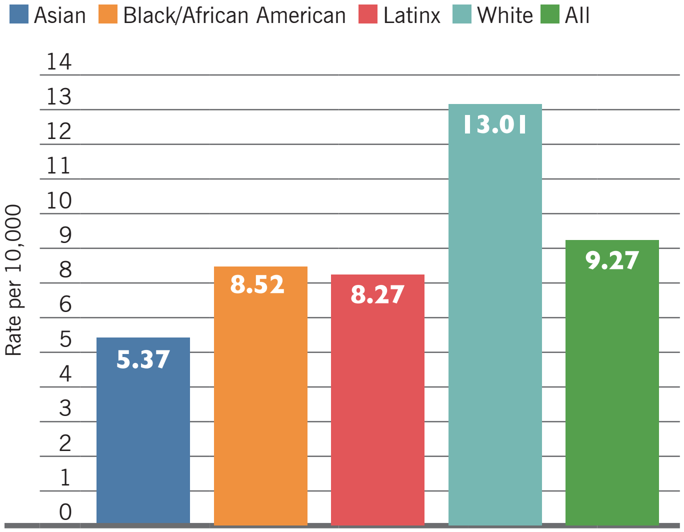

Age-adjusted Rates of Hospitalization* due to Depression by Race/Ethnicity in San Francisco, 2014-2016 [7]

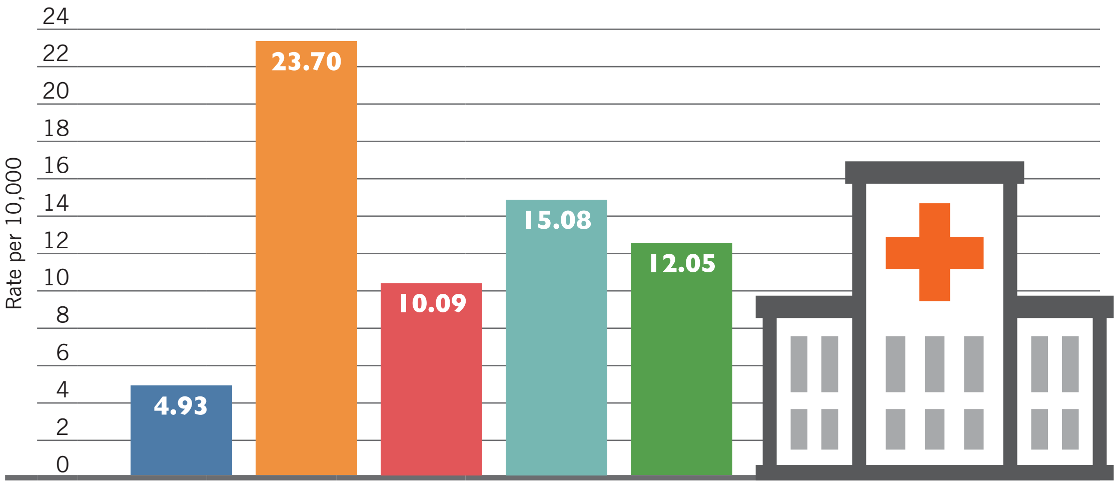

* Hospitalization rates are not deduplicated (i.e. one person could be hospitalizated many times. High rates of hospitalizations among Black/African American likely result from inadequate access to medical care.

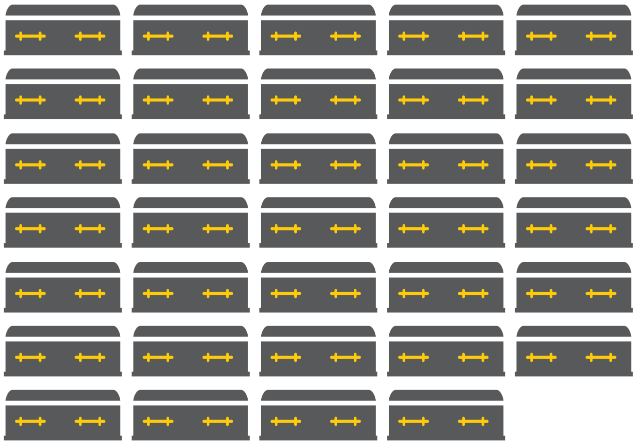

Suicide is the 12th leading cause of death in San Francisco.[8]

114 San Franciscans committed suicide between 2015-2017.

50.96 years is the average age of death for those who complete suicide.

Suicide completion is **3 times more common** among men than women (14.22 vs 4.95 per 100,000 population).

The suicide rate is the highest in the **Castro Neighborhood**.

Social, Emotional, and Behavioral Health

Alcohol abuse is common in San Francisco

2 out of 5 (40%) adults reported binge drinking in 2014–2015.[13]

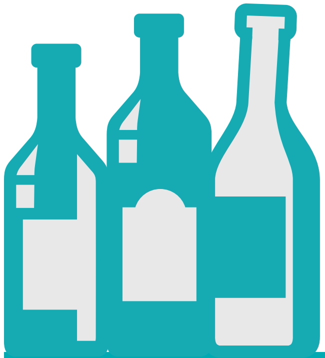

Over half (53%) of men and **24%** of women over 18 binge drink.

8.37% of SFUSD high school students reported binge drinking in 2013–2017.[12]

1 out of 4 (25%) white students binge drink, which is 2–12 times higher than other race/ethnicities.

3 out of 5 (61%) young adults 18–24 years binge drink.[6]

Binge drinking is defined as consuming 5 or more alcoholic drinks for men and 4 or more for women on at least one occasion.

Percentage of SFUSD HS Students Who Reported Binge Drinking in the Past 30 Days by Race/Ethnicity, 2013-2017[5]

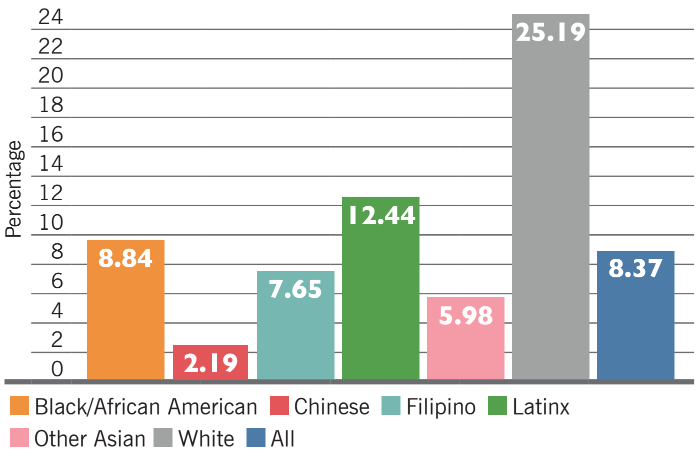

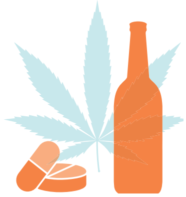

Many factors determine whether someone will start to use or become dependent on drugs or alcohol

Risk factors for use among children and adolescents include:

- Unstable family relationships
- Exposure to physical, mental, and sexual abuse
- Mental illness
- Early aggressive behavior
- Poor social skills
- Poor academic performance
- Substance use among peers and family members
- Involvement with the juvenile justice system
- Poverty[16,17]

The effects of drug and alcohol use are cumulative, and significantly contribute to costly social, physical, mental, and public health problems. These problems include:

- Poor academic performance
- Cognitive functioning deficits
- Unintended pregnancy
- HIV and other sexually transmitted diseases
- Hepatitis C
- Motor vehicle crashes
- Violence
- Child abuse
- Crime, homicide
- Chronic diseases including liver disease and certain cancers (e.g. colorectal, liver, breast, prostate)
- Mental and behavioral disorders (unipolar depressive disorders, epilepsy, suicide)[11]

Youth in San Francisco are at risk of substance abuse[5]

27%
of SFUSD high school students and 6% of middle school students have smoked marijuana.

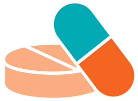

12%
of SFUSD high school students and 3% of middle school students have abused prescription drugs.

8% of SFUSD high school students / **6%** of middle school students > have used methamphetamines, inhalants, ecstasy or cocaine.

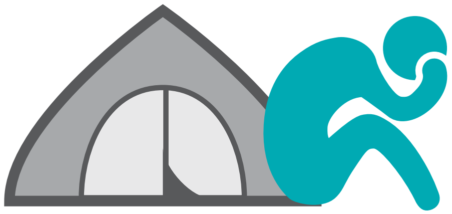

Drug and alcohol abuse contribute to homelessness in San Francisco

15%
of homeless persons reported drug and alcohol use as their primary cause of homelessness in 2017.[13]

65%
of chronically homeless persons reported alcohol or substance use.

Social, Emotional, and Behavioral Health

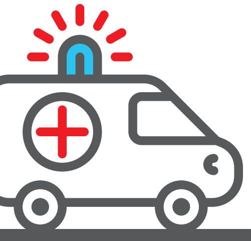

Between 2014 and 2016, 8,552 emergency room visits resulted from alcohol abuse and 8,245 from drugs.[7]

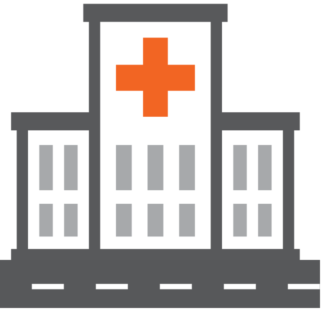

Rates of Emergency Room Visits by Ethnicity and Age, 2012-2016[7]

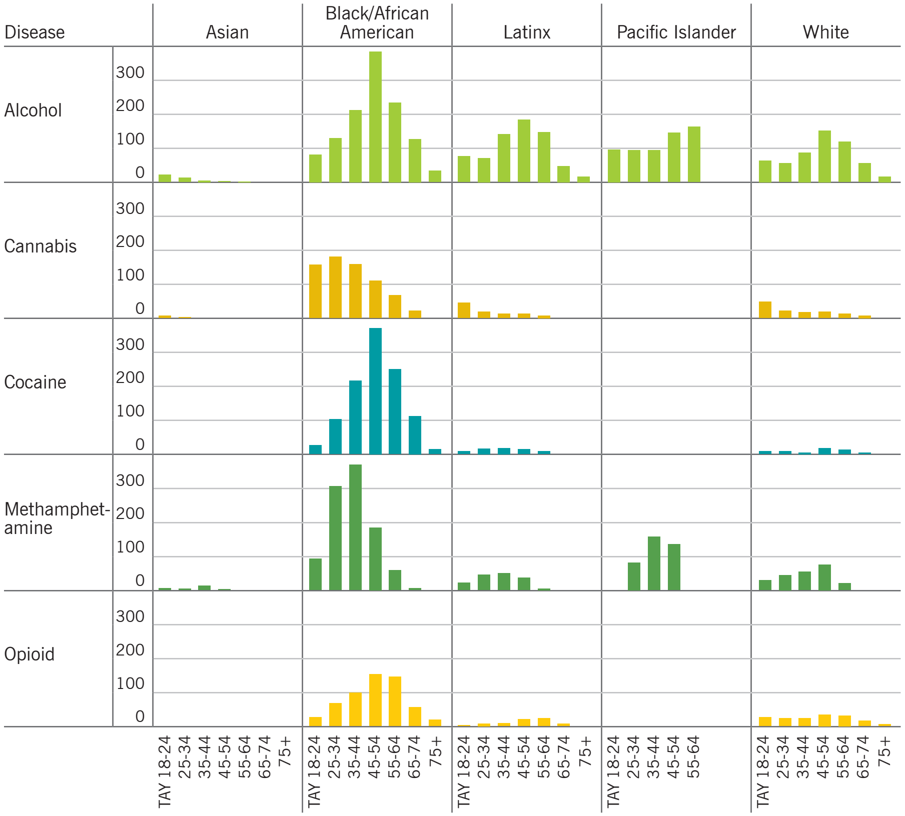

Data represent primary, contributing, and co-morbid causes of emergency room visits

Age-adjusted Rates of ER Visits due to Alcohol Abuse by Zip Code, 2012–2016, and off-site alcohol permits in San Francisco.[7,12]

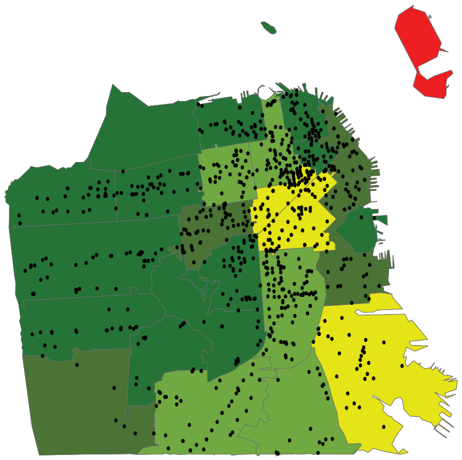

Neighborhoods with higher density of off-sale alcohol outlets coincide with those with higher rates of emergency room visits due to alcohol abuse.

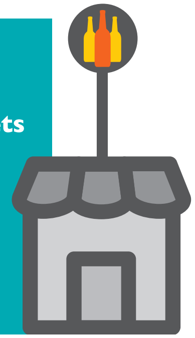

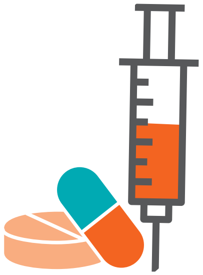

Neighborhoods with larger Black/African American populations like Tenderloin and South of Market also have much higher emergency room visit and death rates due to drug abuse.[8]

Age-adjusted Mortality Rates due to Drug Use Disorders by Race/Ethnicity in San Francisco, 2015–2017[8]

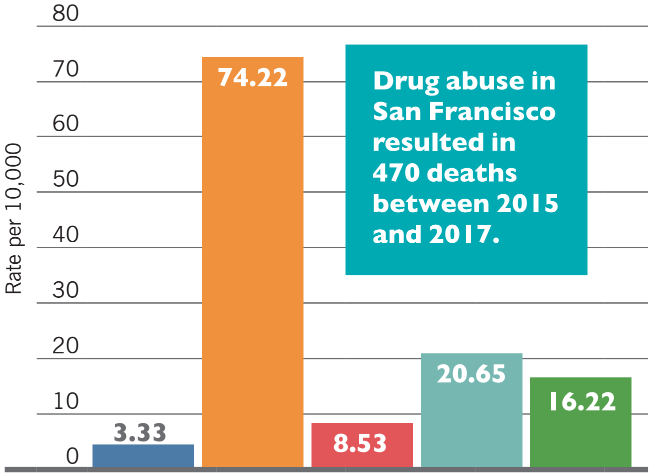

Drug abuse in San Francisco resulted in 470 deaths between 2015 and 2017.

Social, Emotional, and Behavioral Health

sfhip

San Francisco spends nearly $400 million a year on tobacco-related costs, including medical expenses, loss of productivity, and secondhand smoke exposure.[14]

Significant gains against smoking have been made, but not everybody has benefited from tobacco control policies and education campaigns.

In 2015-2016, **11%** of adults in San Francisco reported they were current cigarette smokers. Young adults and low income earners residents are disproportion-ately affected by tobacco.[13]

17% vs 9% Residents who live under 200% federal poverty level are twice more likely to smoke than those live above 200% federal poverty level.

15% vs 5%
Men are 3 times more likely to smoke than women.

16% vs 10%
18 to 24 years are more likely to smoke than those 25 and older.

Percent of High School Students Who Smoked Cigarettes in the Past 30 Days by Race/Ethnicity in San Francisco, 2013–2017[5]

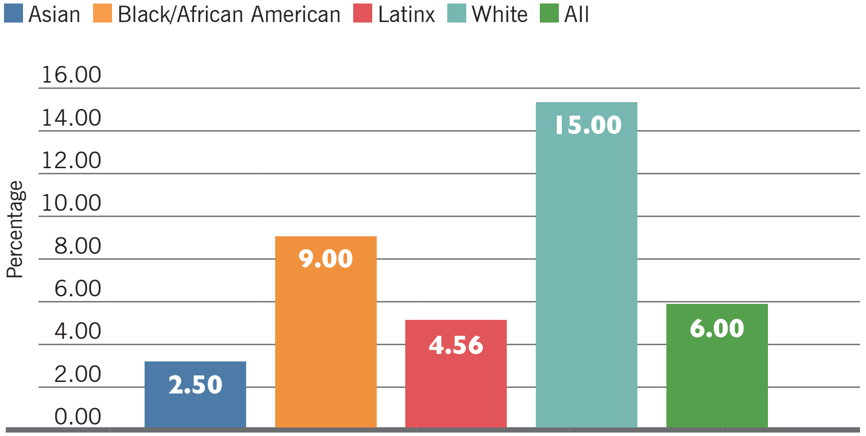

Number of Cigarette Packs Sold by Zip Code, 2016

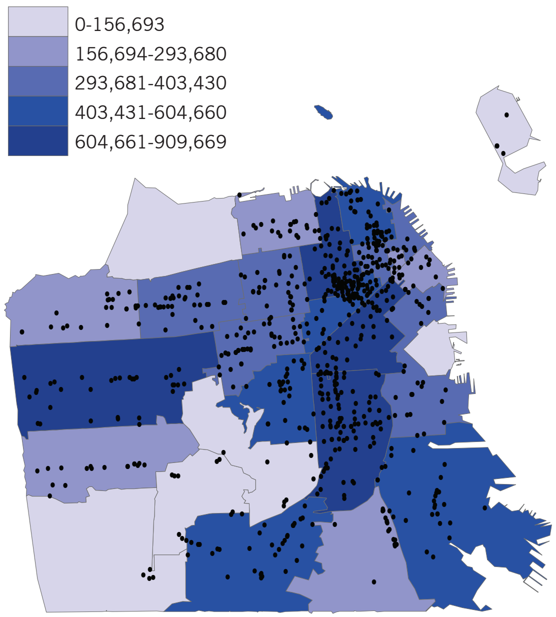

Since adoption of the Tobacco Permit Density Reduction Ordinance in 2014, the number of tobacco retailers has declined by 18%.

The reduction was 26% in the Tenderloin and SOMA districts which had the highest density of retailers.[14]

From 2015 to 2016, the number of packs of cigarettes sold in San Francisco fell by 10%.[14]

E-cigarette use

In 2017, while 4% of SFUSD high school students reported smoking cigarettes, 7% reported using e-cigaretes or other electronic smoking devices in the last 30 days.[5]

25% of SFUSD high school students reported ever using e-cigarretes or other electronic smoking devices.[5]

"Vaping" is on the rise, especially among young people, which caused the US Surgeon General to call for aggressive steps to curb the epidemic of teen nicotine use in 2018.[15]

To limit e-cigarette use among youth in San Francisco the following laws have been passed:

2014: prohibition of the use of electronic cigarettes wherever smoking of tobacco products is prohibited.

2016: raised the minimum age to purchase tobacco products from 18 to 21.

2018: banned flavored tobacco products sales including flavored electronic tobacco pods.

7% vs 1%
of Black/African American women are 7x more likely to smoke before or during pregnancy.[4]

References

Executive Summary

1. "Housing And Health: An Overview Of The Literature," Health Affairs Health Policy Brief, June 7, 2018. DOI: 10.1377/ hpb20180313.396577

Approach

1. Health Resources & Services Administration Maternal & Child Health. Life course approach in mch. https://mchb.hrsa.gov/ training/lifecourse.asp, November 2017.
2. World Health Organization et al. Preamble to the constitution of the world health organization, as adopted by the international health conference, new york, pp. 19-22 (june 1946): signed on 22 july 1946 by the representatives of 61 states,(official records of the world health organization, no. 2 p. 100) and enterd into force on 7 april 1948. http://www. who. int/abouwho/en/ definition. html, 1948.
3. Centers for Disease Control and Prevention. Nchhstp social determinants of health. definitions. https://www.cdc.gov/nchhstp/ socialdeterminants/definitions.html, March 2014.
4. California Planning Roundtable. The social determinants of health for planners: Live, work, plan, learn! https://cproundtable.org/ static/media/uploads/publications/sdoh/cpr_sdoh_final_1-26-16. pdf, 2016.
5. HealthyPeople.gov. Social determinants of health. http://www. healthypeople.gov/2020/topics-objectives/topic/social-determi-nants-health, 2017.

San Francisco Snapshot

1. US Census Bureau. American Community Survey. https://www. census.gov/programs-surveys/acs/, 2012-16.
2. State of California Department of Finance. Report p-2: County Population Projections (2010-2060) by Age. Sacramento, California, 2018.
3. State of California Department of Finance. Report p-2: County Population Projections (2010-2060) by Race/Ethnicity. Sacramento, California, 2018.
4. US Census Bureau. American Community Survey. https://www. census.gov/programs-surveys/acs/, 2017.
5. US Census Bureau. American Community Survey. https://www. census.gov/programs-surveys/acs/, 2013-2017.

Poverty

1. Paula Braveman, Susan Egerter, and Colleen Barclay. Exploring the Social Determinants of Health: Income, Wealth and Health. Technical Report, Robert Wood Johnson Foundation, 2011.
2. www.countyhealthrankings.org. County Health Rankings & Roadmaps. http://www.countyhealthrankings.org/, 2018.
3. US Census Bureau. American Community Survey. https://www. census.gov/programs-surveys/acs/, 2012-16.
4. HealthCare.gov. Federal Poverty Level (FPL). https://www. healthcare.gov/glossary/federal-poverty-level-fpl/, Accessed 12/3/18.
5. Insight Center for Community Economic Development. The Self-Sufficiency Standard for California. http://www.selfsufficien-cystandard.org/california, 2018.
6. State of California Employment Development Department. Labor Market Information. http://www.labormarketinfo.edd.ca.gov/, Accessed 2018.
7. We Defined "Middle Income" Jobs as between 80-120% AMI (per Brookings Institute). In 2014 the 80% AMI for 1 person was 54,350.
8. US Census Bureau. American community survey. https://www. census.gov/programs-surveys/acs/, 2017.
9. Alan Berube. City and Metropolitan Income Inequality Data Reveal Ups and Downs through 2016. https://www.brookings. edu/research/city-and-metropolitan-income-inequality-data-revea l-ups-and-downs-through-2016/, February 2018.
10. Office of Statewide Health Planning and Development. Patient Discharge Dataset. http://www.oshpd.ca.gov.
11. California Department of Education. Fitnessgram®, 2012-2017.
12. San Francisco Unified School District (SFUSD)-San Francisco Department of Public Health (SFDPH) Dental Services. Kindergarten oral health screening program.
13. Vital Statistics California Department of Public Health. Births statistical master file.

Racial Health Inequities

1. California Department of Public Health. Maternal infant health assessment survey. https://www.cdph.ca.gov/Programs/CFH/ DMCAH/MIHA/Pages/default.aspx.
2. California Department of Public Health. Birth statistical master file.
3. U.S. Census Bureau. American community survey, 2012-2016.
4. California Department of Education. Dataquest. https://www.cde. ca.gov/ds/sd/cb/dataquest.asp.
5. San Francisco United School District. Facts at a glance, 2018.
6. Centers for Disease Control and Prevention (CDC). Youth risk behavior surveillance system. https://www.cdc.gov/healthyyouth/ data/yrbs/index.htm.
7. California's Office of Statewide Health Planning and Development. Patient discharge data and emergency department data. https:// oshpd.ca.gov/.
8. San Francisco Unified School District (SFUSD)-San Francisco Department of Public Health (SFDPH) Dental Services. Kindergar-ten oral health screening program.
9. San Francisco Juvenile Probation Department. 2017 statistical report.
10. San Francisco Department of Homelessness and Supportive Housing. Homeless point in time count. http://hsh.sfgov.org/ research-reports/san-francisco-homeless-point-in-time-count-re-ports/, 2017.
11. City and County of San Francisco. Report of the blue ribbon panel on transparency, accountability, and fairness in law enforcement. www.SFBlueRibbonPanel.com, 2016.
12. City and County of San Francisco Police Department. SFPD second quarter 2017 report in compliance with administrative code 96a. https://sanfranciscopolice.org/2017-admin-code-96a-reports, 2017.
13. City and County of San Francisco Police Department. SFPD third quarter 2017 report in compliance with administrative code 96a. https://sanfranciscopolice.org/2017-admin-code-96a-reports, 2017.

sfhip

14. City and Police Department County of San Francisco. SFPD first quarter 2017 report in compliance with administrative code 96a. https://sanfranciscopolice.org/2017-admin-code-96a-reports, 2017.

15. City and County of San Francisco Police Department. SFPD fourth quarter 2017 report in compliance with administrative code 96a. https://sanfranciscopolice.org/2017-admin-code-96a-reports, 2018.

16. U.S. Census Bureau. American community survey, 2013-2017.

17. California Department of Public Health. VRBIS death statistical master file plus 2006-2017, January 31, 2018.

18. San Francisco Mayor's Task Force on African American Out-Migration. Report of the San Francisco Mayor's Task Force on African-American Out-Migration 2009. http://sf-hrc.org/sites/default/fi les/Documents/Policy_Division/African_American_Leadership_Council/African_American_Out_Migration_2009.

Access to Coordinated, Culturally and Linguistically Appropriate Services Across the Continuum

1. Healthy People 2020, "Access to Health Services." http://www.healthypeople.gov/2020/topics-objectives/topic/Access-to-Health-Services

2. San Francisco Department of Public Health and the San Francisco Planning Department,"San Francisco Health Care Services Master Plan. October 2013." https://www.sfdph.org/dph/

3. Covered California; SF Human Services Agency, and San Francisco Department of Public Health, 2014 Enrollment for Region 4.

4. American Communities Survey. 2017

5. UCLA Center for Health Policy Research. California Health Interview Survey. 2016-17.

6.California Department of Public Health. Maternal and infant Health Assessment. 2013-15.

7. SFDPHSFUSD-SFDS Kindergarten Oral Health Screening Program. 2015.

8. Office of Statewide Health Planning and Development, Patient Discharge Dataset, 2016.

9. Office of Statewide Health Planning and Development, Emergency Department Dataset, 2016.

10. Mancoske, R; Lewis, M; Bowers-Stephens, C; et al. (2012) "Cultural Competence and Children's Mental Health Service Outcomes." Journal of Ethnic & Cultural Diversity in Social Work. 21(3): 195-211. 1

11.Mazor, S; Hampers, L; Chande, V; et al. (2002) "Teaching Spanish to Pediatric Emergency Physicians: Effects on Patient Satisfaction." 156(7): 693-5.

Food Insecurity, Healthy Eating, and Active Living

1. United States. Department of Agriculture. Myplate. http://www.choosemyplate.gov/MyPlate

2. The United States Department of Agriculture, American Medical Association, Americans with Disabilities Associations, American Heart Association, and the American Academy of Pediatrics.

3. Institute of Medicine. (2004) Dietary Reference Intakes: Water, Potassium, Sodium, Chloride, and Sulfate. https://iom.nationalacademies.org/Reports/2004/Dietary-Reference-Intakes-Water-Potassium-Sodium-Chloride-and-Sulfate.aspx

4. Centers for Disease Control and Prevention. Behavoiral Risk factors Surveillance System. 2013

5. WIC Program Eat SF WIC participant survey, 2017.

6. Food Security Task Force. Assessment of Food Security. 2018.

7. OSHPD. Rates of Preventable Hospitalizations for Selected Medical Conditions by County (LGHC Indicator). https://data.chhs.ca.gov/dataset/rates-of-preventable-hospitalizations-for-selected-medical-conditions-by-county8. YRBS Youth Risk Behavioral Surveillance System. http://www.cdc.gov/healthyyouth/data/yrbs/index.htm

9. SFDPH Child Care Health Program. 2016-2017 Change in Fruit & Vegetable. Intake https://www.sfdph.org/dph/files/MCHdocs/Epi/Data-Table-Early-Childhood-Change-in-Fruit-and-Vegetable-Intake-2016-2017.pdf

10. San Francisco Food Security Task Force FY 2017-18 & FY 2019-20 Funding Request. https://www.sfdph.org/dph/files/mtgsGrps/FoodSecTaskFrc/docs/FSTF-Budget-Request-FY-18-19.pdf Accessed 8-24-2018.

11. USDA Economic Research Service

12. https://waterfill.me/

13. San Francisco Chronical."Let's ban 'for customers only' policies". 2018. https://www.sfchronicle.com/opinion/openforum/article/Let-s-ban-for-customers-only-policies-12865050.php homeless individuals.

14. San Francisco Department of Public Health. San Francisco Indicator Project.

15. World Health Organization (WHO), "Global Strategy on Diet, Physical Activity and Health. "http://www.who.int/dietphysicalactivity/factsheet_young_people/en/

16. Moore SC, et al. "Leisure Time Physical Activity of Moderate to Vigorous Intensity and Mortality: A Large Pooled Cohort Analysis." PLoS Medicine. November 6, 2012. doi: 10.1371/journal.pmed.1001335

17. Centers for Disease Control and Prevention, Physical Activity and Health. 2011. Available at: http://www.cdc.gov/physicalactivity/everyone/health/

18. CHIS California Health Interview Survey. http://askchisne.ucla.edu

19. SFDPH Childcare Health Program. https://www.sfdph.org/dph/comupg/oprograms/MCH/Epi.asp

20. YRBS Youth Risk Behavioral Surveillance System. http://www.cdc.gov/healthyyouth/data/yrbs/index.htm

21. Better Streets Plan San Francisco. Chapter 2.9. https://www.sf-planning.org/ftp/BetterStreets/docs/FINAL_2_Context.pdf

22. City and County of San Francisco. City Performance Scorecards. https://sfgov.org/scorecards/transportation/traffic-fatalities

23. Walk San Francisco. https://walksf.org/get-involved/take-action/

24. San Francisco Indicator Project. Bike Lanes and Paths. https://www.sfindicatorproject.org/indicators/view/188

25. SFC San Francisco Controller's Office, "San Francisco City Survey," http://sfcitysurvey.weebly.com/

26. San Francisco Parks Alliance. https://www.sfparksalliance.org/

27. CDE The California Department of Education, FitnessGram® physical fitness test. http://dq.cde.ca.gov/dataquest/

sfhip

28. American Community Survey. 2012-16

29. The San Francisco Department of Public Health's Child Care Health Program

30. American Communities Survey, 2013-2017

32. Surrey, Kent. Academic Health Science Network, Wessex Academic Health Science Network, and NE Hants and Farnham CCG. The hydrate toolkit. 2016. https://wessexahsn.org.uk/img/projects/Hydration%20toolkit%2016.pdf

33. San Francisco Marin Food Bank.

34. Vision Zero SF. https://visionzerosf.org/

Housing Security and an end to Homelessness

1. J. Woetzel, J. Mischke, S. Peloquin, and D. Weisfield, "A Tool Kit to Close California's Housing Gap: 3.5 Million Homes by 2025," McKinsey Global Institute, Oct. 2016.

2. Metropolitan Transportation Commission, "Plan Bay Area 2040," 2017.

3. "Regional Housing Need Allocation," Association of Bay Area Governments. [Online]. Available: https://abag.ca.gov/planning/housingneeds/. [Accessed: 24-Aug-2018].

4. US Census Bureau, "American Community Survey (ACS) 1-year Estimates," 2017. [Online]. Available: https://factfinder.census.gov/faces/nav/jsf/pages/index.xhtml.

5. P. Braveman, M. Dekker, S. Egerter, T. Sadegh-Nobari, and C. Pollac, "Exploring the social Determinants of Health: Housing and Health." Robert Wood Johnson Foundation, May-2011.

6. DataSF, "Total eviction notices by year since 1997," Sf Housing Data Hub, 2018. .

7. Applied Survey Research, "2017 San Francisco Homeless Count & Survey Comprehensive Report," San Francisco, CA, 2017.

8. R. Henderson, "Homelessness is a public health crisis," The Guardian, 22-Jul-2014. [Online]. Available: https://www.theguardian.com/society-professionals/2014/jul/22/homelessness-public-health-crisis. [Accessed: 05-Sep-2018].

9. American Public Health Association (APHA), "Housing and Homelessness as a Public Health Issue," American Public Health Association, 07-Nov-2017. [Online]. Available: https://www.apha.org/policies-and-advocacy/public-health-policy-statements/policy-database/2018/01/18/housing-and-homelessness-as-a-public-health-issue.

10. Zillow, "Data: Median Rent List Price ($), 2-Bedroom," Zillow Research, 2018. [Online]. Available: https://www.zillow.com/research/data/. [Accessed: 30-Nov-2018].

11. National Low Income Housing Coalition, "Out of Reach 2018: California," National Low Income Housing Coalition, 2018. [Online]. Available: https://nlihc.org/oor/california. [Accessed: 30-Nov-2018].

12. US Census Bureau, "American Community Survey (ACS) 5-year Estimates," 2017-2013. [Online]. Available: https://factfinder.census.gov/faces/nav/jsf/pages/index.xhtml.

Safety from Violence and Trauma

1 Perez-Smith AM, Albus KE, Weist MD. 2001. "Exposure to violence and neighborhood affiliation among inner-city youth." Journal of Clinical Child & Adolescent Psychology, 30(4):464–72.

2. Ozer EJ, McDonald KL. 2006. "Exposure to violence and mental health among Chinese American urban adolescents." Journal of Adolescent Health, 39(1):73–9.

3. Ackard DM, Neumark-Sztainer D. "Date violence and date rape among adolescents: associations with disordered eating behaviors and psychological health." Child Abuse & Neglect, 2002;26:455 – 473. 10.

4. Howard DE, Wang MQ. "Psychosocial correlates of U.S. adolescents who report a history of forced sexual intercourse." Journal of Adolescent Health, 2005;36:372–379.

5. Fullilove MT, Heon V, Jimenez W, Parsons C, Green LL, Fullilove RE. 1998. "Injury and anomie: effects of violence on an inner-city community." American Journal of Public Health, 88(6):924

6. Sampson RJ, Raudenbush SW, Earls F. 1997. "Neighborhoods and violent crime: a multilevel study of collective efficacy." Science, 277:918–924.

7. Putnam R. 2000. "Bowling Alone: The Collapse and Revival of American Community." New York, NY: Simon & Schuster.

8. Kennedy BP, Kawachi I, Prothrow-Stith D, Lochner K, Gupta V. 1998. "Social capital, income inequality, and firearm violent crime." Social Science & Medicine, 47:7–17.

9. Prevention Institute. 2005. "A Lifetime Commitment to Violence Prevention: The Alameda County Blueprint." http://www.preventioninstitute.org/alameda.html. Retrieved 7/6/2006

10. PolicyLink. 2002. "Reducing health disparities through a focus on communities." Oakland, CA: A PolicyLink Report.

11. Geronimus A. 2001. "Understanding and eliminating racial inequalities in women's health in the United States: the role of the weathering conceptual framework." JAMWA 56(4):133–136.

12. California Department of Justice, Crimes and Clearances. https://openjustice.doj.ca.gov/crime-statistics/crimes-clearances, Accessed November 2018.

13. Office of Statewide Health Planning and Development. Emergency department data. http://www.oshpd.ca.gov.

14. San Francisco Police Department. https://data.sfgov.org/public-safety/police-department-incident-reports-historical-2003/tmnf-yvry.

15. San Francisco Controller's Office. San Francisco city survey. http://sfcitysurvey.weebly.com/.

16. California Department of Public Health. VRBIS death statistical master file plus 2006-2017, January 31, 2018.

17. University of California at Berkeley. California child welfare indicators project. http://cssr.berkeley.edu/ucb_childwelfare/allegations.aspx.

18.Safe and Sounce. The economics of child abuse.https://safeandsound.org/economics-child-abuse-study-san-francisco/19. U.S. Census Bureau. American community survey. https://www.census.gov/programs-surveys/acs.

19. U.S. Census Bureau. American community survey. 2012–2016. https://www.census.gov/programs-surveys/acs/.

20. Brookings. City and metropolitan income inequality data reveal ups and downs through 2016. https://www.brookings.edu/research/city-and-metropolitan-income-inequality-data-reveal-ups-and-downs-through-2016/, 2018.

21. California Dept. of Education. Coordinated school health and safety office custom tabulation & California basic educational data system. www.kidsdata.org: https://www.kidsdata.org/topic/230/homeless-students/table#fmt=355&loc=265&tf=88&sortColumnId=0&sortType=asc, May 2017.

22. California Department of Public Health. Maternal infant health assessment survey. https://www.cdph.ca.gov/Programs/CFH/DMCAH/MIHA/Pages/default.aspx.

23. U.S. Census Bureau. American community survey. 2013-2017. https://www.census.gov/programs-surveys/acs/.

24. UCLA Center for Health Policy Research. California health interview survey. http://healthpolicy.ucla.edu/chis/Pages/default.aspx..

25. City and County of San Francisco Police Department. SFPD second quarter 2017 report in compliance with administrative code 96a. https://sanfranciscopolice.org/2017-admin-code-96a-reports, 2017.

26. City and County of San Francisco Police Department. SFPD third quarter 2017 report in compliance with administrative code 96a. https://sanfranciscopolice.org/2017-admin-code-96a-reports, 2017.

27. City and Police Department County of San Francisco. SFPD first quarter 2017 report in compliance with administrative code 96a. https://sanfranciscopolice.org/2017-admin-code-96a-reports, 2017.

28. City and County of San Francisco Police Department. SFPD fourth quarter 2017 report in compliance with administrative code 96a. https://sanfranciscopolice.org/2017-admin-code-96a-reports, 2018.

29. Christopher Wildeman and Emily A Wang. Mass incarceration, public health, and widening inequality in the USA. Lancet (London, England), 389:1464–1474, April 2017.

30. Zinzi D Bailey, Nancy Krieger, Madina Agénor, Jasmine Graves, Natalia Linos, and Mary T Bassett. Structural racism and health inequities in the USA: evidence and interventions. Lancet (London, England), 389:1453–1463, April 2017.

31. City and County of San Francisco. Report of the blue ribbon panel on transparency, accountability, and fairness in law enforcement. www.SFBlueRibbonePanel.com, 2016.

32. Emily Owens; Erin Kerrison; and Bernardo Santos De Silveira. Examining racial disparities in criminal case outcomes among indigent defendants in sf. https://www.law.upenn.edu/live/files/6793-examining-racial-disparities-may-2017-full, 2017.

33. ACLU. School-to-prison pipeline, Accessed November 2018.

34. Susan Ferriss. An epidemic of questionable arrests by school officials. https://www.huffingtonpost.com/entry/san-bernadino-arrests_us_5669b21ce4b009377b24119e, 2015.

35. The San Francisco Police Department and The San Francisco Unified School District. Memorandum of understanding between the San Francisco police department and the San Francisco unified school district. http://www.ncjfcj.org/sites/default/files/Memorandum%20of%20Understanding%20San%20Francisco%20Police%20and%20School%20Dis.pdf, 2014.

36. San Francisco Board of Education. Establishment of safe and supportive school policy in the San Francisco unified school district. http://www.sfusd.edu/en/assets/sfusd-staff/about-SFUSD/files/resolutions/HANEY%20SAFE%20SUPPORTIVE%20POLICY%20FINAL%20FOR%20POSTING%202%2025%2014.pdf, 2014.

37. SFUSD San Francisco Unified School District. 2016-2017 district data summary newsletters. http://web.sfusd.edu/Services/research_public/201617%20District%20Data%20Summary%20Newsletters/Forms/AllItems.aspx.

38. San Francisco Juvenile Probation Department. 2017 statistical report.

39. San Francisco Mayor's Task Force on Anti-Human Trafficking. Human Trafficking in San Francisco. 2017. https://safeandsound.org/wp-content/uploads/2018/12/MTF-Human-Trafficking-2017-Data-Report-112618.pdf

Social, Emotional, and Behavioral Health

1. World Health Organization. Mental disorders: fact sheet. Media centre: Mental disorders – Fact sheet No. 396 (October 2014), 2017. Accessed 10.23.17.

2. World Health Organization. Mental health: Strengthening our response. http://www.who.int/mediacentre/factsheets/fs220/en/, 2016. Accessed 10.23.17.

3. Centers for Disease Control and P. (CDC). Mental health basics. http://www.cdc.gov/mentalhealth/basics.htm, 2013. Accessed 10/23/2017.

4. California Department of Public Health. Maternal infant health assessment survey. https://www.cdph.ca.gov/Programs/CFH/DMCAH/MIHA/Pages/default.aspx.

5. Centers for Disease Control and Prevention (CDC). Youth risk behavior surveillance system. https://www.cdc.gov/healthyyouth/data/yrbs/index.htm.

6. UCLA Center for Health Policy Research. California health interview survey. http://healthpolicy.ucla.edu/chis/Pages/default.aspx.14. California's Office of Statewide Health Planning and Development. Patient discharge data and emergency department data. https://oshpd.ca.gov/.

7. California Department of Public Health. Vrbis death statistical master file plus 2006-2017, January 31, 2018.

8.National Institute on Drug Abuse. Preventing drug use among children and adolescents. http://www.drugabuse.gov/publications/preventing-drug-abuse-among-children-adolescents/chapter-1-risk-factors-protective-factors/what-are-risk-factors, 2003.

9. National Institute on Drug Abuse. Drugs, brains, and behavior: The science of addiction. http://www.drugabuse.gov/publications/drugs-brains-behavior-science-addiction/drug-abuse-addiction, 2014.

10. Centers for Disease Control and Prevention. Alcohol and public health: Fact sheets- alcohol and your health. https://www.cdc.gov/alcohol/fact-sheets/alcohol-use.htm, 2016.

11. San Francisco Indicator Project. Alcohol outlet density. www.sfindicatorproject.org, 2014.

12. San Francisco Department of Homelessness and Supportive Housing. Homeless point in time count. http://hsh.sfgov.org/research-reports/san-francisco-homeless-point-in-time-count-reports/, 2017.

13. San Francisco Tax Collector. Packs of cigarettes sold in san francisco, 2012-2016. https://sftreasurer.org/.

14. Richard Miech, Lloyd Johnston, Patrick M. O'Malley, Jerald G. Bachman, and Megan E. Patrick. Adolescent vaping and nicotine use in 2017–2018 — u.s. national estimates. The New England Journal of Medicine, 2018.

San Francisco Community Health Needs Assessment 2019

POPULATION HEALTH DIVISION
SAN FRANCISCO DEPARTMENT OF PUBLIC HEALTH

Prepared by the San Francisco Department of Public Health, Population Health Division

For inquiries contact Michelle.Kirian@sfdph.org

CHINESE HOSPITAL
Caring for the Community for the Next 100 Years

Dignity Health.
Saint Francis Memorial Hospital

Dignity Health.
St. Mary's Medical Center

Hospital Council
of Northern & Central California
Excellence Through Leadership & Collaboration

JEWISH HOME
Jewish Senior Living Group

KAISER PERMANENTE®

Sutter Health
We Plus You

UCSF Health
